# The PFAS roadmap–Navigating a path together to improved management

**DOI:** 10.1088/2977-3504/ae286f

**Published:** 2026-02-16

**Authors:** Lokesh P Padhye, Melanie Kah, Erin M Leitao, Karl Bowles, Paul Nathanail, Ian T Cousins, Romain Figuière, Bradley O Clarke, Jordan M Partington, Wejdan Alghamdi, Elsie M Sunderland, Bridger J Ruyle, Satoshi Endo, Trevor N Brown, Zhengyang Wang, Size Zheng, Joseph J Pignatello, Sanne J Smith, Marcel Riegel, Hans Peter H Arp, Jens Blotevogel, Robert J Giraud, Anthony K Rappé, Erlend Sørmo, Gerard Cornelissen, Marc A Deshusses, Igor V Novosselov, P Lee Ferguson, Brian R Pinkard, Timothy J Strathmann, Kapish Gobindlal, Jonathan Sperry, Elisabeth Cuervo Lumbaque, Nick Duinslaeger, Jelena Radjenovic, James Hatton

**Affiliations:** 1 The New York State Center for Clean Water Technology, Stony Brook University, Stony Brook, NY 11794, United States of America; 2 School of Marine and Atmospheric Sciences, Stony Brook University, Stony Brook, NY 11794, United States of America; 3 Department of Civil Engineering, Stony Brook University, Stony Brook, NY 11794, United States of America; 4 School of Environment, The University of Auckland, 23 Symonds Street, 1010 Auckland, New Zealand; 5 School of Chemical Sciences, The University of Auckland, 23 Symonds Street, 1010 Auckland, New Zealand; 6 The MacDiarmid Institute for Advanced Materials and Nanotechnology, Victoria University of Wellington, Wellington, New Zealand; 7 Jacobs Group, North Sydney 2060, Australia; 8 LQM, Nottingham NG7 2TU, United Kingdom; 9 Department of Environmental Science, Stockholm University, Stockholm, Sweden; 10 Australian Laboratory for Emerging Contaminants, School of Chemistry, University of Melbourne, Victoria 3010, Australia; 11 Department of Chemistry, College of Science, University of Bisha, PO Box 551, Bisha 61922, Saudi Arabia; 12 Harvard John A. Paulson School of Engineering and Applied Sciences, Cambridge, MA 02138, United States of America; 13 Tandon School of Engineering, New York University, Brooklyn, NY 11201, United States of America; 14 Health and Environmental Risk Division, National Institute for Environmental Studies (NIES), Onogawa 16-2, 305-8506 Tsukuba, Ibaraki, Japan; 15 ARC Arnot Research & Consulting, Toronto, Ontario, Canada; 16 Department of Chemistry, Stony Brook University, Stony Brook, NY 11794, United States of America; 17 Department of Environmental Science and Forestry, The Connecticut Agricultural Experiment Station, New Haven, CT 06511, United States of America; 18 Department of Water Management, Delft University of Technology, Stevinweg 1, 2628 CN Delft, The Netherlands; 19 TZW: DVGW-Technologiezentrum Wasser, Karlsruher Str. 84, 76139 Karlsruhe, Germany; 20 Environmental Technology, Norwegian Geotechnical Institute (NGI), Sandakerveien 140, 0484 Oslo, Norway; 21 Department of Chemistry, Norwegian University of Science and Technology (NTNU), Høgskoleringen 5, 7491 Trondheim, Norway; 22 Commonwealth Scientific and Industrial Research Organisation (Waite Campus), Urrbrae, SA 5064, Australia; 23 Department of Chemical and Biomolecular Engineering, University of Delware, Newark, DE 19716, United States of America; 24 Department of Chemistry, Colorado State University, Fort Collins, CO 80523, United States of America; 25 Faculty of Environmental Sciences and Natural Resource Management (MINA), Norwegian University of Life Sciences (NMBU), Elizabeth Stephansensvei 31, 1433 Ås, Norway; 26 Norwegian Institute for Bioeconomy Research (NIBIO), Oluf Thesens vei 43, 1433 Ås, Norway; 27 Department of Civil and Environmental Engineering, Box 90287, Duke University, Durham, NC 27708, United States of America; 28 374Water Inc., 701 W. Main Street, Suite 410, Durham, NC 27701, United States of America; 29 Mechanical Engineering Department, University of Washington, Seattle, WA 98195, United States of America; 30 Aquagga, Inc., 748 Market St, Tacoma, WA 98402, United States of America; 31 Civil and Environmental Engineering Department, Colorado School of Mines, Golden, CO 80401, United States of America; 32 Environmental Decontamination (NZ) Limited. 1/22 Highgate Parkway, Auckland 0932, New Zealand; 33 Centre for Green Chemical Science, University of Auckland. 23 Symonds Street, Auckland 1010, New Zealand; 34 Catalan Institute for Water Research (ICRA), Emili Grahit 101, 17003 Girona, Spain; 35 Catalan Institution for Research and Advanced Studies (ICREA), Passeig Lluís Companys 23, 08010 Barcelona, Spain; 36 Jacobs, Greenwood Village, CO 80111, United States of America; 37 Department of Civil and Environmental Engineering, The University of Auckland, Auckland 1010, New Zealand; 38 Guest editors of the Roadmap

**Keywords:** PFAS, PFAS treatment, PFAS alternatives, PFAS monitoring, PFAS analysis, PFAS fate, PFAS management

## Abstract

Per- and polyfluoroalkyl substances (PFAS) represent a large—and structurally diverse—group of contaminants that have become ubiquitous in our environment. PFAS are all extremely persistent while some are also bioaccumulative, mobile and/or toxic, which gives rise to significant environmental and health concerns. Despite more than a decade of intensive research, the management of PFAS is still associated with considerable challenges. It is evident that a holistic approach is required to address the challenging global problem of PFAS. This roadmap features expert perspectives from world-renowned leading researchers and practitioners on how best to manage PFAS. The 15 topics cover different facets of the complex PFAS issue, providing a multidisciplinary and multisectoral overview. For each topic, we reflect on the current status of knowledge and offer recommendations on science and technology advances that will help meet current and future challenges. Taken together, the 15 topics cover the entire life cycle of PFAS—from their sources to their destruction. Important themes such as monitoring and analysis, understanding and predicting fate, source controls (regulation and replacement), and existing and emerging strategies for remediation (capture and destroy) are highlighted throughout the roadmap. Overall, there are many recent scientific and technological advancements that show promise for the management of PFAS. However, it is also clear that there is no ‘silver bullet’ and multifaceted solutions will be needed. Long-term success hinges on sustained collaboration among researchers, policymakers, industries, and communities, which we hope this roadmap will help to catalyze.

## 1. Introduction

### Lokesh P Padhye^1,2,3,4^, *Melanie Kah*^5^ and Erin M Leitao^6,7^

^1^ The New York State Center for Clean Water Technology, Stony Brook University, Stony Brook, NY 11794, United States of America

^2^ School of Marine and Atmospheric Sciences, Stony Brook University, Stony Brook, NY 11794, United States of America

^3^ Department of Civil Engineering, Stony Brook University, Stony Brook, NY 11794, United States of America

^4^ Department of Civil and Environmental Engineering, The University of Auckland, Auckland 1010, New Zealand

^5^ School of Environment, The University of Auckland, 23 Symonds Street, 1010 Auckland, New Zealand

^6^ School of Chemical Sciences, The University of Auckland, 23 Symonds Street, 1010 Auckland, New Zealand

^7^ The MacDiarmid Institute for Advanced Materials and Nanotechnology, Victoria University of Wellington, Wellington, New Zealand

**Figure 1.**
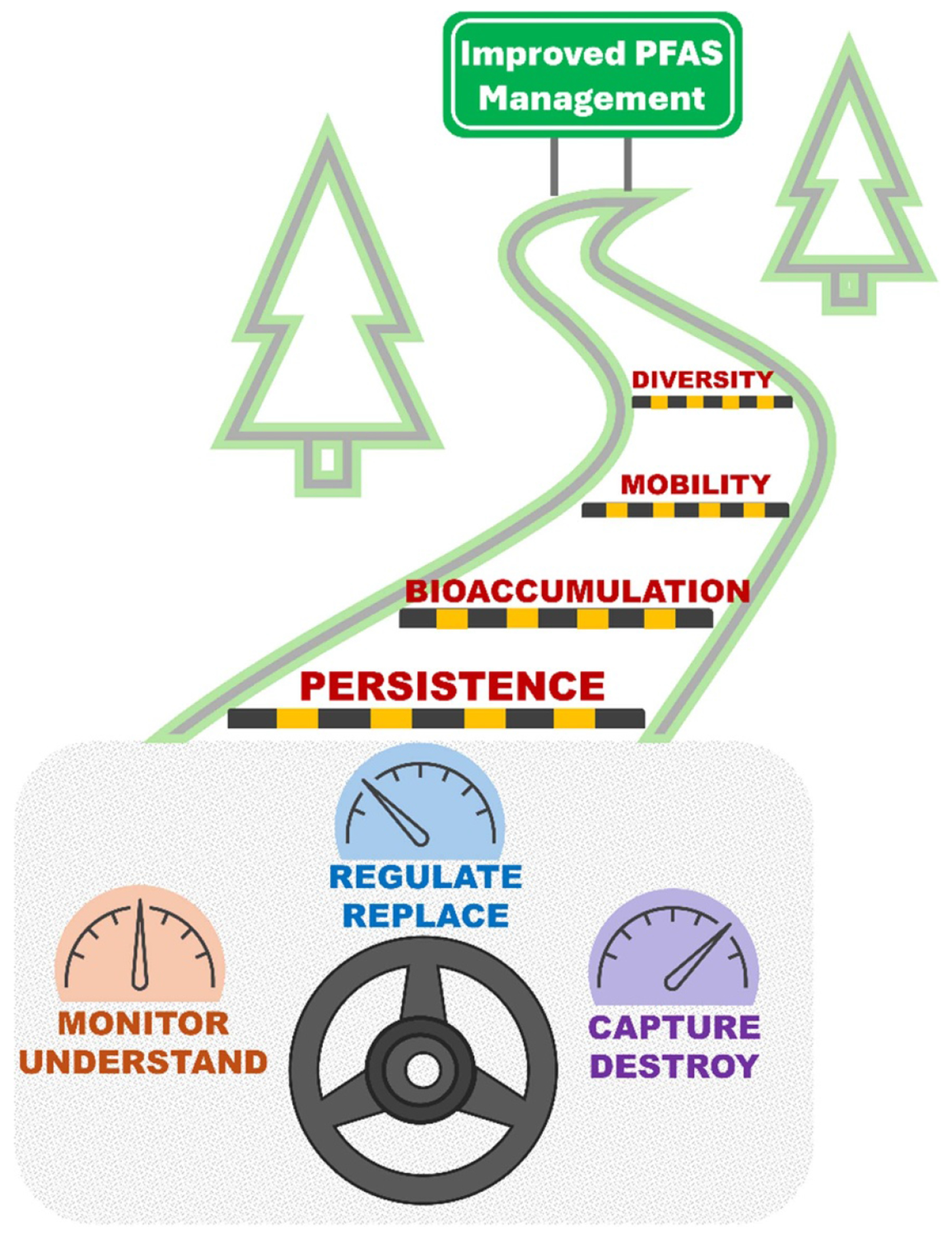
Towards improved PFAS management using the three essential pillars.

Per- and polyfluoroalkyl substances (PFAS), often called ‘forever chemicals’, represent one of the most pressing environmental challenges of our time. Many PFAS are persistent, widespread, and have harmful health impacts, which calls for innovative solutions and collaborative efforts across academia, industry, and government. This roadmap integrates expert perspectives from world-renowned leading researchers and practitioners to address and manage the risks posed by PFAS. The roadmap is divided into 15 sections, comprehensively covering the three pillars required for a holistic approach: Monitor and Understand, Regulate and Replace, and Capture and Destroy (figure 1).

### Monitor and understand

Effectively addressing PFAS contamination begins with robust monitoring and a comprehensive understanding of their fate as well as environmental and health impacts. The expanding catalog of PFAS and their diverse chemical properties pose significant analytical challenges. As highlighted in section 4, there is a need for the continued advancement of analytical methods to detect PFAS at the trace concentrations associated with regulatory standards as well as detect currently unknown PFAS. Cutting-edge technologies such as liquid chromatography-(LC) tandem mass spectrometry (MS) and high-resolution MS play pivotal roles in this quest, but there is also a need for real-time and *in situ* monitoring.

Environmental monitoring programs must also bridge existing data gaps to account for PFAS prevalence across different media (section 5). These programs should prioritize high-risk PFAS while adopting innovative techniques to understand PFAS partitioning. For instance, section 6 emphasizes the importance of studying fluorinated compounds that fall outside traditional PFAS definitions to grasp their environmental fate fully.

The scale of PFAS contamination necessitates research to deepen our understanding of their behavior. As highlighted in section 7, sorption dynamics significantly influence PFAS transport and accumulation in natural systems while simultaneously being essential to engineering strategies for removing PFAS. Informed by these insights, future research should incorporate interdisciplinary approaches spanning environmental chemistry, toxicology, and engineering to develop more effective monitoring strategies.

### Regulate and replace

The second pillar focuses on source control through regulatory frameworks and transitioning to safer and sustainable alternatives to PFAS. The establishment of regulatory approaches still faces hurdles due to the complexity and scale of PFAS contamination (section 2). Clear definitions and standardized groupings of PFAS are essential for establishing consistent, risk-based management practices across jurisdictions. However, low cleanup thresholds, differing stakeholder expectations, and disagreements over what constitutes ‘practicable’ remediation targets add layers of complexity to regulatory efforts.

Phasing out non-essential PFAS while avoiding regrettable substitution is a key strategy for reducing PFAS usage in industrial and consumer applications. section 3 underscores the importance of corporate investment and innovation in phasing out PFAS. Resources like an online substitution database offer a valuable starting point, but the industry must rigorously test alternatives to ensure their safety and effectiveness. Ideally, the move toward PFAS-free products is driven not only by regulatory compliance to address environmental concerns but also by the opportunity for industries to position themselves as forward-thinking leaders in sustainable manufacturing.

Adapting to a dynamic regulatory landscape requires proactive engagement from all stakeholders. Section 15 emphasizes the need for evolving health-based guidance and ecological protection values, which are challenging for the application of both established and emerging remediation technologies. In this context, collaborative efforts can harmonize regulatory standards and encourage the adoption of sustainable practices.

### Capture and destroy

Capturing and destroying PFAS is crucial to mitigating environmental and health impacts. Traditional remediation methods, such as sorption, are central to removing PFAS from contaminated water and soil. As highlighted in section 7, technologies like granular activated carbon (GAC) and anion exchange resins (AER) are widely used, but they generate significant waste that must be managed in the long term.

Section 8 outlines liquid-phase enrichment, membranes, foam fractionation, and regenerative IEX, that up-concentrate PFAS so downstream destruction units can be smaller. Membranes deliver high PFAS removal efficiency but face fouling and weaker short-chain rejection. Foam fractionation attains a high volume reduction factor (VRF) yet removes short-chains poorly unless additives are used and requires off-gas controls. Regenerative IEX offers fast PFAS uptake but has difficulty in regeneration. Hybrid trains (e.g. GAC upstream + weak-base resin) can boost short-chain capture and achieve very high VRFs.

Incineration of waste and spent sorbents, as discussed in section 9, remains a critical tool but still requires rigorous research to optimize its efficacy and safety. Emerging thermal technologies offer promising alternatives to incineration. Supercritical water oxidation (SCWO), described in section 11, demonstrates high efficacy in mineralizing PFAS and co-contaminants. Its scalability and sustainability make it a strong candidate for widespread adoption, provided further research optimizes its design and life-cycle assessments. Similarly, section 12 highlights the potential of hydrothermal alkaline treatment (HALT) for mineralizing PFAS in industrial wastewater, offering advantages such as low energy consumption, handling of waste with very high concentrations of PFAS, and integration with existing treatment systems.

Destructive technologies must also address diverse waste streams and environmental conditions. For example, section 13 outlines the potential of ball milling to degrade PFAS in solids, while section 10 explores pyrolysis as a dual solution for biochar production and PFAS immobilization in soils. Integrating these methods with existing waste management practices could yield cost-effective, large-scale solutions.

Section 14 sheds light on the transformative potential of electrochemical oxidation (EO) systems. These systems enable modular, multifunctional treatment trains that integrate adsorbents, membranes, and electrodes for PFAS removal. Advances in electrode design and reactor configuration hold promise for compact systems capable of treating diverse water types. The ability to completely destroy PFAS at the point of treatment is critical to addressing health and environmental risks.

Finally, *in situ* remediation, as discussed in section 15, represents a critical strategy for addressing PFAS contamination in soils and groundwater. Yet, its efficacy is constrained by the diversity of PFAS and evolving regulatory landscapes. Immobilization, while valuable, may be insufficient for long-term solutions. Shifting the focus toward removal and destruction while balancing sustainability remains a pressing need.

### The path forward

Tackling PFAS contamination requires an integrated strategy spanning monitoring, regulation, substitution, separation, and destruction. Each pillar of the PFAS Roadmap contributes to a cohesive framework for addressing the unique challenges posed by PFAS. While scientific and technological advancements offer hope, long-term success hinges on sustained collaboration among researchers, policymakers, industries, and communities.

Despite the advancements highlighted in each section of this roadmap, significant knowledge gaps remain. For instance, research on the partitioning behavior of PFAS and their interactions with co-contaminants (section 6) is essential to improve modeling and prediction. Likewise, the lack of full-scale data for promising technologies like ball milling (section 13) and pyrolysis (section 10) limits their immediate applicability.

The PFAS Roadmap demonstrates that a multifaceted approach is indispensable to managing this complex challenge. For example, addressing volatile PFAS analytical challenges, as highlighted in section 15, may introduce regulatory challenges for wastewater treatment plants and landfill management. Hence, holistic solutions must account for all pillars of effective PFAS management. This calls for investment in research, capacity building, and the development of standardized protocols to guide technology adoption.

As section 2 aptly concludes, achieving sustainable PFAS remediation requires reconciling technical feasibility with societal expectations. By embracing risk-based frameworks, investing in advanced technologies for analysis, monitoring, and remediation, and fostering cross-sectoral partnerships, we can move closer to a future where the environmental and health impacts of PFAS are effectively mitigated.

*Note: This Roadmap is not a comprehensive catalog of all PFAS technologies; it highlights a subset for which our contributor team could synthesize state-of-practice across the three pillars with sufficient field evidence and operational detail. Accordingly, some promising approaches, e.g. UV/solvated- or hydrated-electron reduction, non-thermal plasma, sonolysis, bioremediation, and photocatalysis, etc are not covered here.

## 2. Regulation of PFAS

### Karl Bowles^1^ and Paul Nathanail^2^

^1^ Jacobs Group, North Sydney, 2060, Australia

^2^ LQM, Nottingham NG7 2TU, United Kingdom

### Status

PFAS include millions of potential synthetic chemicals. PFAS frequently headline in both mainstream and social media, often under their colloquial, if not strictly accurate, moniker of ‘forever chemicals.’

Modern regulation of such manufactured chemicals includes controlling entry to the market, licensing authorized releases, managing unauthorized pollution events, and managing historical contamination. Managing contamination is done either through ensuring land and water is made safe and suitable for its next use through land use planning or by direct regulatory enforcement for high-risk situations. This paper primarily focuses on regulation to manage historical contamination, with relevance also to managing unauthorized pollution.

The need for public authorities to promulgate rules to protect human health and the environment from the hazards of substances used by society was crystallized in 1962 by Carson’s *Silent Spring* [1]. She warned of the dangers of what we now know as persistent organic pollutants (POPs) on humans and ecosystems.

Between Carson and other actions [2], wheels were set in motion that saw the establishment of the US Environmental Protection Agency (1970), UK Department of the Environment (1970) and Victoria Environmental Protection Agency (1971). Subsequently, individual countries and some regions have developed environmental frameworks, often in response to a major incident.

It was only in the 1990s, with the development of LC MS, that the environmental impacts of PFAS became more widely known. Since then, regulation has changed frequently, especially in last 10 years, due to rapidly emerging science and widespread societal concerns.

In 2001, almost 4 decades after *Silent Spring*, the Stockholm Convention for POPs was signed to globally manage the most hazardous organic chemicals. PFOS, its salts and perfluorooctane sulfonyl fluoride was listed in Annex B in 2009, restricting their use. PFOA and PFHxS, their salts and related compounds were listed in Annex A in 2019 and 2022, respectively, leading to stopping their use [3]. More recently the Conference of Parties to the Convention agreed to list the long chain perfluorocarboxylic acids (LC-PFCAs) as a group [4].

Another notable recent change in PFAS guidance are the listings of PFOA as carcinogen to humans (Group 1) and PFOS as possibly carcinogenic to humans (Class 2b) by IARC [5]. Such listings can have flow on effects, such as the USEPA setting a health based maximum contaminant level goal (MCLG) in drinking water of zero. The MCLG is a goal and not legally enforceable. In comparison, the MCLs are based on achievability (analytical and treatability) and are enforceable, following a phase in time frame. In April 2024 the USEPA released MCLs for PFOS PFOA, and four other PFAS under a hazard quotient approach [6]. In May 2025, following legal challenges, the USEPA announced that while the PFOS and PFOA values would be finalized, it was considering pulling back on MCLs for the other four PFAS [7]. This highlights the contentious nature of setting enforceable values at low concentrations (ng L^*−*1^) that may be technically achievable but impact on other societal pressures such as financial costs to communities.

**Figure 2.**
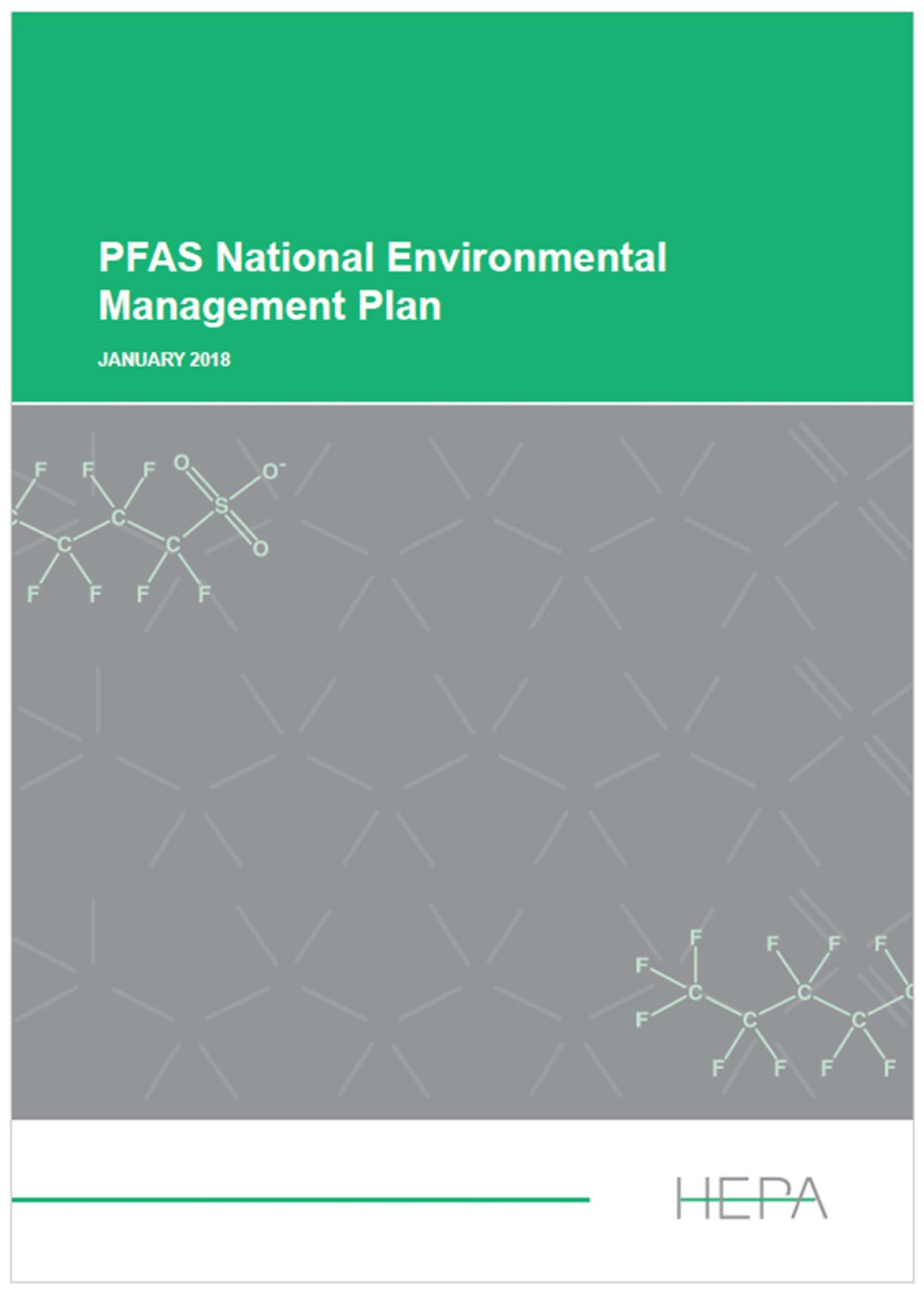
The PFAS National Environmental Management Plan v1.0 (HEPA 2018) was one of the first attempts globally at comprehensive guidance for managing environmental contamination of PFAS. Australia and New Zealand released v2.0 of the PFAS NEMP in 2020, at the time of writing v3.0 was forthcoming, (PFAS NEMP 1.0, Courtesy of Commonwealth of Australia).

Most frameworks for managing land and water contamination are risk-based (e.g. figure 2). That is, below certain identified thresholds of harm, the presence of a chemical does not require actions to clean up. The growing societal awareness of and concern about PFAS contamination of soil and water is in danger of driving regulatory action towards managing the presence of PFAS rather than the risks they pose. A strong scientific understanding of the complexities of PFAS is needed to help ensure management responses and costs to society are proportionate, while providing the necessary protections for human and the environment.

### Current and future challenges

**Figure 3.**
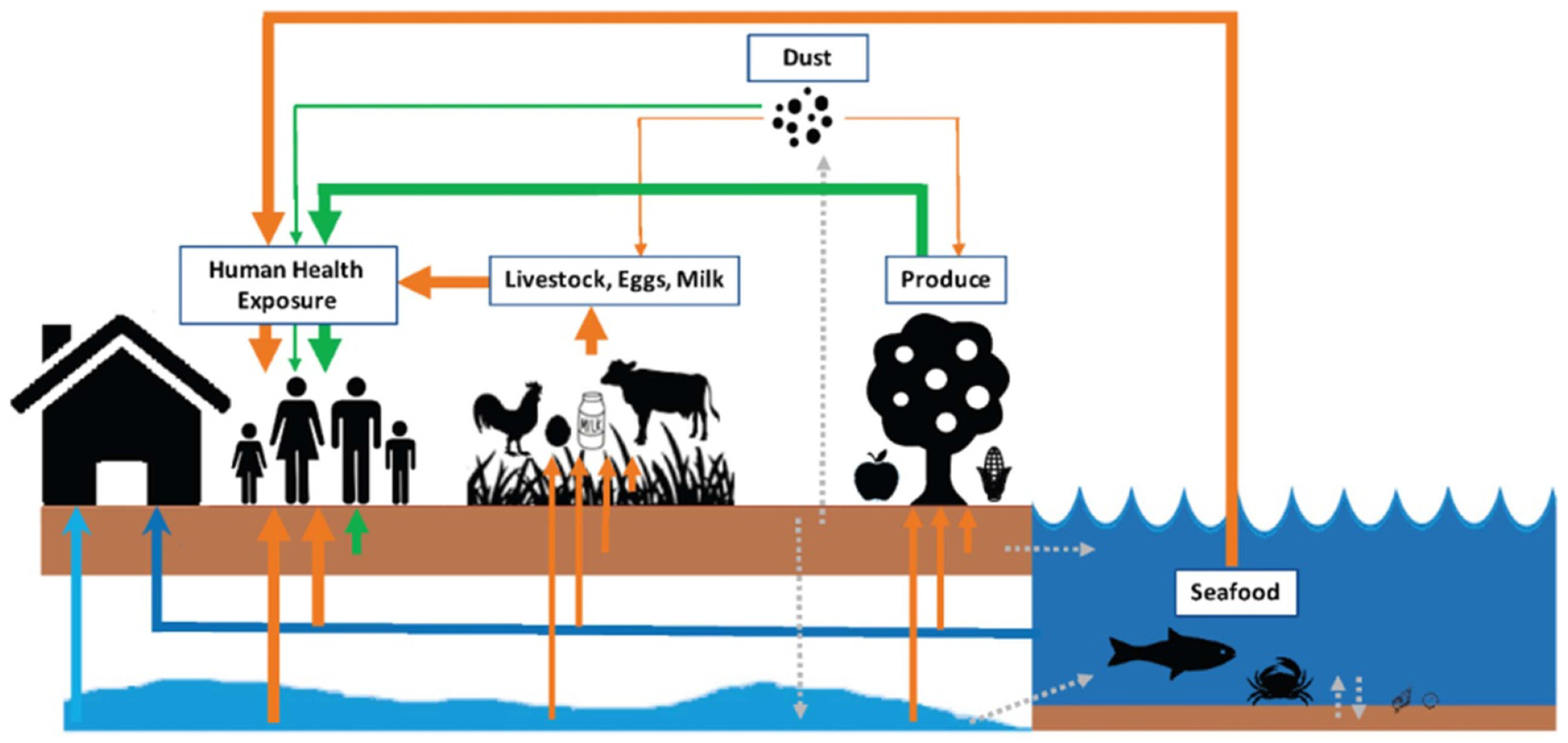
Generic conceptual model of PFAS transport leading to human exposures. The enormous diversity of PFAS physicochemical properties means a wide variety of transport pathways are relevant (PFAS NEMP 2.0, Courtesy of Commonwealth of Australia).

PFAS regulation is complicated by their often unusual and extremely wide range of physicochemical properties, transport pathways, and toxicity (figure 3). Some key examples are summarized below.

#### Definition

The OECD definition of PFAS spans organic chemicals with at least one saturated CF_2_ or CF_3_ moiety through to polymers [8]. The panel deriving the OECD definition foreshadowed the concern this may pose for regulation and recommended that working definitions may be needed for specific applications [8]. Some countries, struggling with the inherent difficulty of providing risk-based thresholds for individual PFAS, or developing risk-based groupings, have instituted non-risk based regulatory groupings of PFAS (e.g. [9]). Disagreements in the definition of PFAS have featured heavily in news media in the last year (i.e. 2025). These reflect discord between manufacturing sectors seeking to maintain certain areas of PFAS manufacture and largely academic sector advocates who point out that providing a comprehensive definition of PFAS does not preclude regulators focusing on subsets of the PFAS for specific purposes [10].

#### Complexity of chemical structures

Most well-known are the perfluoroalkyl acids (PFAAs). These have been widely used commercially and are terminal products of environmental degradation of precursor PFAS. Despite the emphasis of research on PFAAs, many other PFAS structures have been identified in products and the environment [11]. Precursor PFAS have non-fluorinated moieties that are amenable to biotic and abiotic degradation. Many pharmaceuticals and pesticides are also PFAS according to the OECD definition by virtue of often a single fully fluorinated carbon. For these, environmental hazards are driven by different mechanisms to PFAS developed for fluorosurfactant behavior or other properties, and very different regulatory frameworks are relevant. Some countries/blocs are managing (or are proposing to manage) all PFAS as a single class. For some purposes, this introduces challenges to risk-based regulation. The very different health-based thresholds for different PFAS mean that setting a single regulatory value for all PFAS must be inherently either over-conservative or under-conservative, depending on how it is framed [12].

#### Bioaccumulation

Bioaccumulation of long chain PFAAs occurs in people and animals is driven by protein-philicity. Bioaccumulation of short chain PFAAs in plants is by a different mechanism, largely driven by transpiration and related to solubility in water. Both mechanisms contrast with bioaccumulation of chlorinated POPs which is driven by lipophilicity. Bioaccumulation of PFAS other than PFAAs is not well understood.

#### Fate and transport in soils

Long chain PFAAs and many precursors accumulate and are retained in near surface soils with highly retarded passage to groundwater [13]. Evaporation can lead to wicking of some PFAS to the surface resulting in significant transport in surface runoff [14].

#### Uncertain and contested toxicology and epidemiology

Regulators globally have interpreted available, albeit contested, evidence very differently. Health-based thresholds are orders of magnitude lower where epidemiological evidence has been used, compared to countries relying on laboratory studies with animals [15]. Toxicology of PFAS other than a few PFAAs is not well understood.

#### Very low screening or intervention guidance values

Guidance values in the low ng L^*−*1^ for waters and *μ*g kg^*−*1^ for soils have consequences for regulation: (i) length of plumes are greater than other less persistent, less toxic contaminants [16], (ii) larger area of contaminated soil to reflect lower assessment criteria, (iii) engineering *ex situ* treatment technologies to achieve these levels is extremely difficult, hence effective regulation may need to be based on ‘So Far As Reasonably Practicable’ or ‘as low as reasonably practicable’ rather than risk-based concentration targets, (iv) remediation by *in situ* pathway interruption may provide long term compliance with these targets. The UK annual average environmental quality standard (EQS) for the protection of freshwater is a legally enforceable measure for licensed discharges to environment. For PFOS the AA EQS is 0.65 ng L^*−*1^ [17]. Treating stormwater to achieve compliance for this value at large, impacted sites such as commercial or military airports, is unachievable with available technologies. This represents an issue where a default approach based on more conventional contaminants is seriously challenged by the low health-based criteria relevant for some PFAS.

Ongoing scientific and technological developments are improving ability to manage some of the complexities listed above, but many challenges remain.

### Advances in science and technology to meet challenges

For regulation of PFAS contamination of land and water there are many areas where scientific advances are reducing uncertainty but further advances are needed. These include:

- Analytical chemistry
  - Reliable quantification of a more individual PFAS and availability of certified reference materials
  - Methods for quantifying aggregated groups of PFAS or organic fluorine at environmentally relevant limits of reporting
  - Faster and more mobile analysis amenable for onsite application and ultimately real-time measurements at relevant limits of reporting
- Fate and transport
  - High reliability modeling of PFAS transport in unsaturated soils and saturated aquifers
  - Prediction of PFAS bioaccumulation, especially in aquatic biota where mechanisms of bioaccumulation are currently poorly understood
  - Oral bioaccessibility tests for PFAS
- Toxicology
  - Toxicological understanding of a wider range of PFAS for humans and biota
  - Regulator consensus in interpretation of the relevance of epidemiological evidence and its benchmark dose modeling to define points of departure
  - Relative potency factors for more PFAS and multiple toxicological endpoints
- Treatment technologies
  - Methods for sustainable *in situ* destruction of PFAS are most desirable but currently least available
  - Lower cost, lower energy methods, including treatment trains, for PFAS destruction both in onsite and offsite facilities
  - Simple reliable methods for point of use removal of PFAS from drinking water.

Many of the above areas are discussed in other sections in this Roadmap and therefore not discussed in detail here.

### Concluding remarks

Despite ongoing scientific advances, some challenges to regulation are likely to remain for the foreseeable future. Fundamental to this is the scale of PFAS contamination in relation to low screening values. For example, irrespective of advances in treatment and characterization technologies, extensively impacted groundwater is unlikely to be able to be remediated, and abstracted water quality will continue to depend on approaches such as point of use treatment and administrative management controls.

Ongoing challenges in handling the complexity of the family of PFAS will also remain. Wider agreement on defining and grouping PFAS is needed to support effective risk-based management.

Difficulties in remediating to extremely low concentrations may push further discussion of paradigms such as clean up so far as reasonably practicable. However, this may not be accepted by citizens or policy makers, or at least involve disagreement in what constitutes ‘practicable’ and ‘acceptable’. While such concepts are already included in regulation in some jurisdictions, in others more rules-based approaches may continue to be challenged. A part of this discussion should be consideration of both what is achievable and what is sustainable.

*In situ* remediation generally has a lower greenhouse gas (GHG) emissions footprint than *ex situ* remediation. The low assessment criteria being set for PFAS will drive remediation efforts towards *in situ* pathway interruption or *ex situ* concentration followed by destruction technologies. The latter are much more energy intensive. To minimize emissions of carbon dioxide equivalent gases, renewable energy will need to be used to avoid large carbon footprints. This points to the need for regulatory change to consider other current governmental and societal priorities.

Collectively the challenges listed above have contributed to disparate approaches taken by various countries in regulating or providing regulatory guidance for PFAS. Nevertheless, the risk-based approach can demonstrably deliver safe land and can contain the dispersion of PFAS in the wider soil and water environments.

## Acknowledgements

This contribution represents the views of the authors based on our experiences, engagement with the literature and many conversations with colleagues, clients and contacts around the world. No funding was received for authoring this contribution.

## 3. Finding alternatives to the many uses of PFAS

### Ian T Cousins and Romain Figuière

Department of Environmental Science, Stockholm University, Stockholm, Sweden

### Status

Based on concerns regarding the high persistence of PFAS and other hazardous properties that trigger additional concerns, it has been argued that the ‘non-essential’ uses of PFAS should be phased out [18]. In Europe, such a broad phaseout of PFAS was taken a step towards reality when the national authorities of five European countries submitted a restriction proposal under the REACH Regulation to restrict the manufacture, placing on the market and use of PFAS [19].

It is clear, however, that an immediate ban of all uses of PFAS is impractical. The concept of ‘essential use,’ originally developed under the Montreal Protocol, has been suggested as a tool to guide the phaseout of PFAS [20]. In summary, certain uses of PFAS may be considered ‘essential’ to health, safety or the functioning of society and alternatives may not be currently available. Other uses of PFAS may be found to be ‘non-essential’ and could be eliminated without having to first find alternatives that provide adequate function and performance. For uses of PFAS to be substitutable, suitable alternatives need to be available on the market or made available in the near future. For the broad EU PFAS restriction proposal to be successful, alternatives would need to be available on the market within a period of 12 years (the longest proposed derogation) after the restriction comes into force. In this commentary, we explore the current availability of alternatives for uses of PFAS, determine what is known about the suitability of these alternatives and provide a roadmap for future work on alternatives.

In a recent commentary in Science by Ateia and Scheringer [21], it was recommended that an open data sharing platform on PFAS uses, the functions they deliver, and their potential alternatives should be provided to maximize the collective knowledge on PFAS-free alternatives and to accelerate the transition away from PFAS. We at Stockholm University have already developed such an online ‘alternatives database’ within the ZeroPM project [22] and it has been available online since September 2023 (https://zeropm.eu/alternative-assessment-database/).

### Current and future challenges

We based our alternatives database on the functional substitution approach developed by Tickner *et al* [23]. In the functional substitution approach, the assessor of alternatives should first define the ‘chemical functions’, ‘end-use functions’ and ‘service functions’ of the chemical in a specific use that is to be substituted. Such an approach enables the identification of a wider range of alternatives beyond only dropin chemical substitutes. The alternatives database [22] as well as being a resource for alternatives is a very useful database of the uses and functions of PFAS. The database currently lists 325 different applications (i.e. ‘uses’) of PFAS across 18 use categories (table 1) [22]. Most of the information on PFAS uses was taken from Annex A of the EU’s PFAS restriction proposal but was also complemented with information from other sources. In the ‘alternatives database’, we have currently listed 530 different alternatives, which break down into 162 alternative substances, 163 alternative materials, 128 alternative products, 37 alternative processes and 40 alternative technologies [22].

Another useful online platform known as MarketPlace (https://marketplace.chemsec.org/) is made available by ChemSec (the International Chemical Secretariat). Marketplace is a global business-to-business platform where companies can find safer alternatives to hazardous chemicals. The alternatives in MarketPlace are already on the market and supplier information is directly available. Currently, there are about 100 alternatives to PFAS uses provided in the MarketPlace database and there is an effort underway at ChemSec (as part of the ZeroPM project) to increase the number of available market-ready alternatives to PFAS included in the MarketPlace database. All of the alternatives in the MarketPlace database are also included in the alternatives database developed by Figuière *et al* [22]. In Sweden, we also have The Swedish Centre for Chemical Substitution (www.ri.se/en/centre-chemical-substitution) which provides a service to guide companies, organizations and the public sector to find alternatives to hazardous substances in products and processes. The Centre has developed competence in substituting PFAS in certain use categories in the last few years. There are already a number of success stories in Sweden and internationally where companies have substituted PFAS (in firefighting foams, clothing, cosmetics, non-stick cookware, etc), which can inspire other companies to do the same [20, 21]. For example, many companies have signed on to ChemSec’s PFAS Movement (108 companies at the time of writing) whereby they commit to phasing out from products and processes (https://chemsec.org/pfas/).

**Figure 4.**
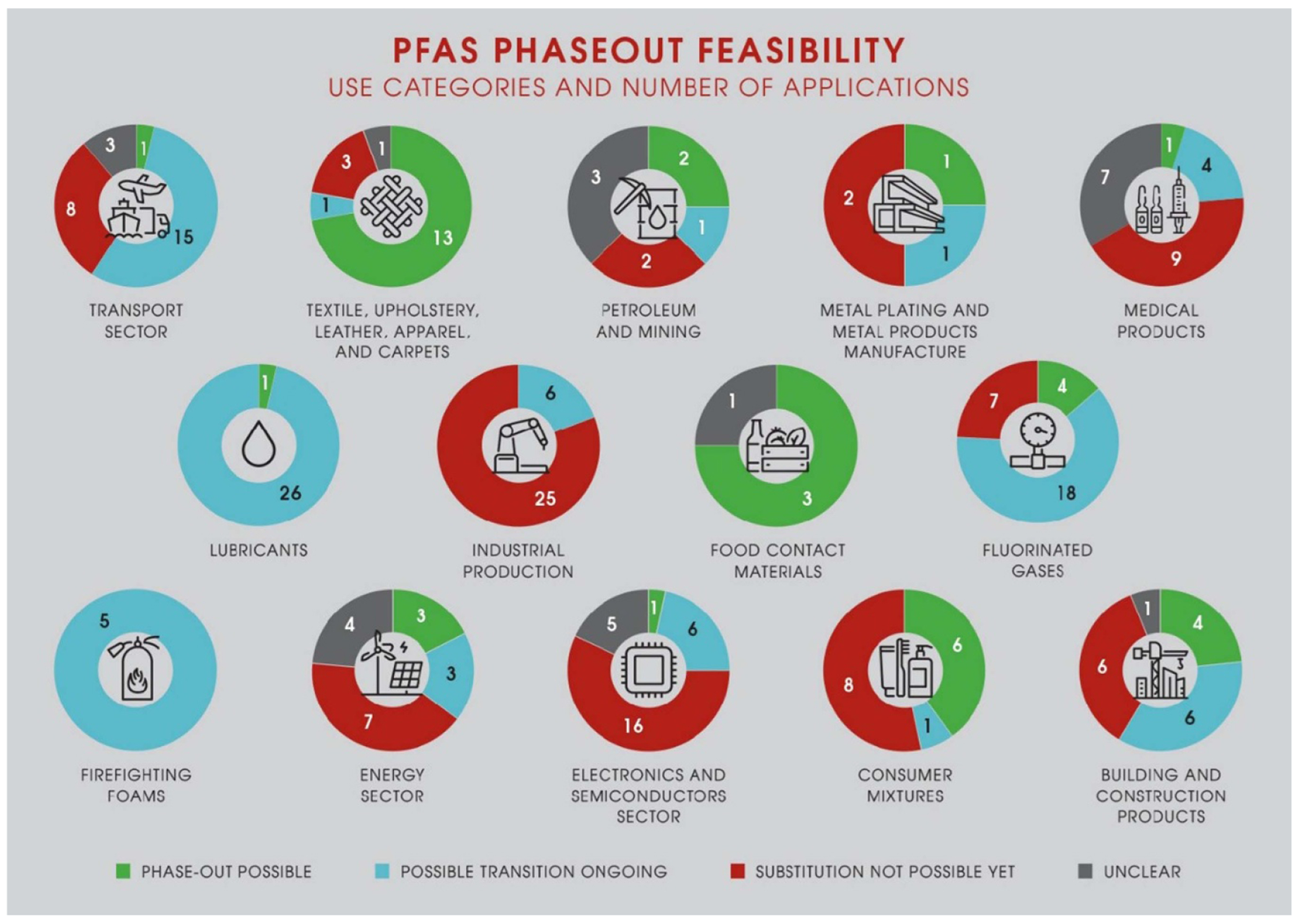
Substitution potential of PFAS for 14 of the 18 PFAS use categories in table 1. This figure is based on figure 3 in [22]. The figure above first appeared in an article in *The Chemical Engineer* Issue 1005 [26] issue 1005 and has been reproduced here with permission from the publisher (IChemE: the Institution of Chemical Engineers). Reproduced with permission from [26].

To successfully substitute, it is important that there is sufficient information provided on alternatives regarding performance, safety and sustainability to avoid regrettable substitutions. Ideally, an alternative to PFAS should have similar functionality, adequate (‘fit for purpose’) technical performance and have minimum environmental and human health impacts throughout its lifecycle. Alternatives assessment frameworks of varying complexity have been developed for comparing selected alternatives with chemicals of concern on the basis of their hazards, performance, and economic viability [24, 25]. However, in our experience, alternative providers do not make enough information available on the alternatives to conduct high-quality alternative assessments. For example, the abovementioned ChemSec MarketPlace does not provide the detailed information needed to conduct alternatives assessments, and there is a degree of trust required in the companies that are posting their PFAS-free products and processes there. In our alternatives database [22], we attempted to evaluate the potential for substituting PFAS in each use category based on available information for the identified alternatives (figure 4).

Of those applications assessed, we concluded that suitable alternatives to PFAS are available for 16% of applications. For 37% of applications, we concluded that alternatives are potentially suitable but require more time for a full transition. For another 37% of applications, there were either no alternatives available or the alternatives were still in the early testing phase or just patented. There are certainly some uses (particularly industrial rather than consumer uses), where the unique properties of PFAS make substitution very challenging (e.g. the substitution of PFAS in semiconductors and lithium ion batteries). For the remaining 10% of applications the information was too unclear to draw conclusions [22].

### Advances in science and technology to meet challenges

We recommend five advances in science and technology needed to address the challenges in developing safe and sustainable alternatives to PFAS:

1. **Material science innovations**. Advances in material science are required to develop alternatives to uses of PFAS, e.g. for industrial uses of fluoropolymers, medical products, and electronics. New materials must replicate PFAS’ unique properties while being safe and sustainable. Artificial intelligence (AI)-driven searches of patents and research can help identify and accelerate the development of these materials.
2. **Enhanced data sharing and transparency**. Whereas it is encouraging that online platforms are now available for sharing information about alternatives to PFAS, greater demands on alternative providers are needed to share more information on their alternatives. In alternatives assessments, it is often the case that we know more about the PFAS-containing products than we know about the alternative products. Improved information on performance and safety of alternatives in these databases would provide greater confidence for companies seeking alternatives.
3. **Understanding PFAS functions.** For some use cases, it requires a large amount of technical knowledge to understand why PFAS are used (i.e. what is the unique combination of functions and performance provided by PFAS?). Without this detailed technical information, it is impossible to determine if substitution is possible. We recommend increased dialogue between different stakeholders to better understand the feasibility of substitution. The POPFREE-project (www.ri.se/en/popfree) is a good example of where such a dialogue has been successful and led to the development of alternatives.
4. **Expanding alternatives assessment.** Continuing work on alternatives assessments [24, 25] is essential, but only if enough data on both PFAS and alternatives is available. Whereas substituting PFAS is important to reduce environmental and health impacts, we should not create new problems by substituting blindly. AI can also play a key role in analyzing large datasets to predict the environmental and performance impacts of potential substitutes, ensuring we avoid creating new problems.
5. **Balancing performance and safety in alternatives.** Compromises on performance may be necessary. PFAS-free products and processes should still be ‘fit for purpose’ and any loss of performance should be offset by the increased safety and sustainability of the alternatives.

### Concluding remarks

In conclusion, while online databases, substitution centers, and other resources serve as useful guides and starting points, moving away from PFAS will require significant effort, time, and investment. Companies must focus on acquiring and testing alternatives to ensure they meet performance standards while being safer and more sustainable. This process should prioritize material science innovations to replace PFAS in key applications. Additionally, enhancing data sharing for greater transparency, fostering deeper understanding of the unique roles PFAS play, and utilizing AI to search for and predict suitable alternatives will be crucial. As more consumers and companies demand the phasing out of PFAS, those that have already succeeded in this transition are setting a strong example, gaining the benefits of sustainability and leadership in innovation.

## Acknowledgements

This work was funded by the EU Horizon 2020 research and innovation programme (Project ZeroPM, Grant Agreement No 101036756).

## 4. PFAS analytical chemistry—current approaches and future trends

### Bradley O Clarke^1^, Jordan M Partington^1^ and Wejdan Alghamdi^1,2^

^1^ Australian Laboratory for Emerging Contaminants, School of Chemistry, University of Melbourne, Victoria 3010, Australia

^2^ Department of Chemistry, College of Science, University of Bisha, Bisha 61922 PO Box 551, Saudi Arabia

### Status

The characterization of PFAS in the environment remains a significant challenge for environmental analytical chemists. The most notorious PFAS are perfluorooctanesulfonic acid (PFOS; C_8_HF_17_O_3_S; CAS RN 1763-23-1) and perfluorooctanoic acid (PFOA; C_8_HF_15_O_2_; CAS RN 335-67-1) which were restricted for use in the early 2000s and subject to voluntary industry phase out in the USA. This led to geographical shifts in production combined with the rapid development of thousands of novel PFAS replacements [27], most without publicly available information on their production and use, that are now finding their way into our environment [28]. Current environmental regulations and analytical methods primarily focus on a limited subset of PFAS, typically 3–25 PFAAs. For example, the U.S. EPA has developed standardized analytical methods for PFAS, including Methods 533 and 537.1 for drinking water. More recently, Method 1633 has been validated, making a significant advancement toward standardized PFAS analysis by encompassing forty PFAS from multiple classes and applying to a broad range of matrices, including aqueous, soil, biosolids and biological tissues. However, with over seven million chemicals on PubChem classified as PFAS [29] it is impossible to monitor and detect all these substances using traditional analytical methodologies. Compounding this challenge is that most manufactured PFAS variants lack publicly available data regarding their production, usage, or impact on public health [28]. With emerging evidence of toxicity from legacy and replacement PFAS (i.e. GenX) at extremely low concentrations, the importance of high-quality analytical methods cannot be overemphasized.

**Figure 5.**
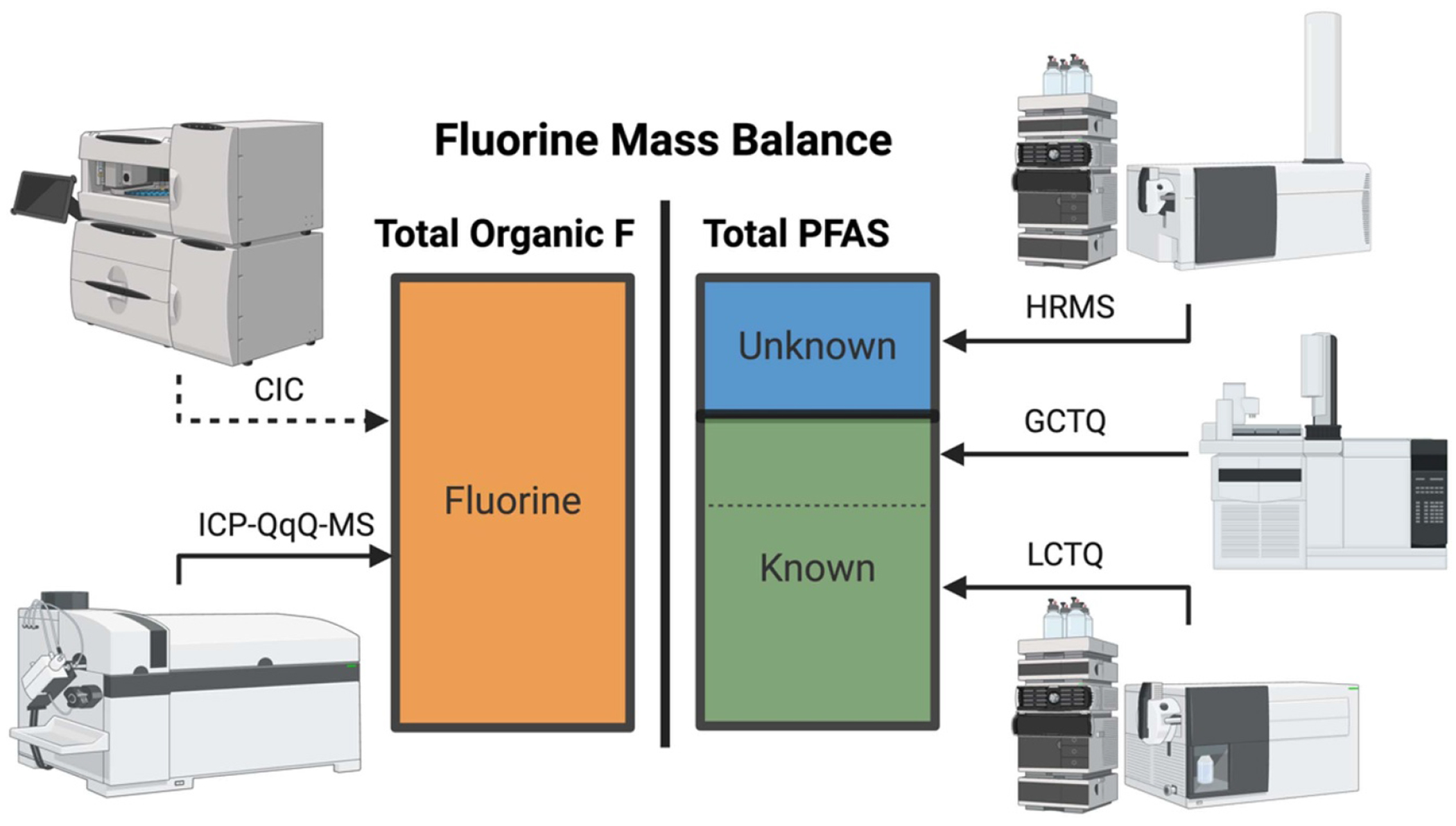
Comprehensive PFAS analysis requires a suite of analytical techniques.

Analytical chemistry underpins all scientific efforts to explain the environmental impacts of pollution. The four key elements involved in analytical chemistry are (1) sample collection, (2) sample extraction, (3) instrumental analysis and (4) data processing. Instrumental analysis typically requires high expertise for operation and large capital investment, while the other three components (collection, extraction, data processing) are frequently time consuming and rate limiting steps for quantitative and qualitative data generation and analysis. Key strategies in understanding and correlating chemical exposure with impact is built upon analytical chemistry for pollutant quantification and unknown substance identification. Typically, analytical measurement approaches are built in a hypothesis driven ‘targeted’ approach. However, monitoring small sets of target compounds can miss important site-specific and potentially toxicologically relevant compounds while typically costing hundreds to thousands of dollars per sample for quantitative measurements. Establishing simple, robust and validated monitoring methodologies will require a comprehensive suite of analytical approaches to fully characterize the PFAS burden in the environment (figure 5).

### Current and future challenges

The most common quantification method for PFAS measurement is called ‘isotope dilution’ and involves electrospray ionization (ESI) reversed-phase LC triple quadrupole MS (LC-TQ) using multiple reaction monitoring (MRM) [30]. Importantly, these methodologies are typically limited to a small subset of legacy anionic PFAS (ESI-mode) and do not measure cationic and zwitterionic (LCTQ; ESI+ mode) [31], ultrashort chain (SFCTQ or ion-exchange LCTQ; ESI-mode) [32] nor semi-volatile PFAS (gas chromatography TQ MS; GCTQ) [33]. TQ-MS is a low-resolution MS technique combining three quadrupoles, two acting as mass analyzers, and the central acting as a collision cell. This arrangement provides high degrees of selectivity and sensitivity, returning precursor and product ion nominal mass data [34]. TQ-MRM approaches require predetermined retention times and transitions based upon expensive authentic standards. Robust reporting requirements necessitates a high level of technical skill and attention to quality control (i.e. blanks, matrix spikes, laboratory control samples) to achieve but most importantly are necessary for the generation of high-quality data. PFAS MRM methods result in very low limits of quantification but typically detect fewer than 80 PFAS due to a combination of factors including instrument dwell time, expensive internal standard requirements, data processing, and extraction techniques that are not generally suited to multiple classes of PFAS [35].

High resolution MS (HRMS) non-targeted analysis (NTA) is a popular technique used to identify and estimate the number of PFAS precursors without reference standards [36]. Both time-of-flight and orbitrap mass analyzers, when coupled with quadrupole mass filters, allow precursor mass filtration, MS/MS fragmentation experiments and high-resolution mass analysis that are within a few parts per million. Control of precursor ions with the quadrupole mass filter allows for both target and non-target data acquisition approaches through data-dependent and data-independent acquisition methods for compound discovery and screening [37]. The integration of ion mobility spectrometry (IMS) into HRMS systems has allowed further resolution of PFAS isomers and differentiation from co-eluting compounds of similar mass via collision cross section (CCS) measurements [38]. While this area has made great advances, standardization of both analytical and data handling processes are an ongoing effort, which has led to questionable reproducibility [39]. Furthermore, interlaboratory comparisons of NTA workflows have demonstrated differences in identification capacity based upon instrument type and parameters, and data-processing techniques and software tools [40]. Despite the capacity for simultaneous target quantification and non-target screening [41], very few research groups quantify and identity PFAS simultaneously leading to orthodoxy difference emerging between quantitative and qualitative research areas. In time we need to see the rigor and quality control expected for quantification applied to the HRMS identification workflow.

### Advances in science and technology to meet challenges

While not as exciting for most researchers as instrumental analysis, extraction methods are arguably the most crucial aspect of PFAS analysis. Extraction has the following broad goals (1) separate PFAS from the media (2) minimize co-extracting materials that can interfere with instrumental methods and (3) allow for the concentration of analytes (viz., removal of solvent) to lower method reporting limits. The most conventional technique utilized to measure the concentration of PFAS in water samples is solid phase extraction (SPE) with weak anion exchange (WAX) sorbent. This utilizes hydrophobic binding mechanisms in conjunction with charged surfaces for PFAS retention. Conventional SPE can use large amounts of water sample (~50–250 ml). However, larger sample volumes increase extraction time and also increase the likelihood of losses or interferences from sample contact with various labware. Ideally, research focused on extraction techniques should be looking to combine extraction with clean-up approaches, reduce sample volume (or mass), coupled with instrument optimization. Extraction techniques should be optimized for the simultaneous quantification of legacy, non-polar volatile, zwitterionic and cationic PFAS using LCTQ/GCTQ and untargeted qualitative analysis with HRMS through the careful evaluation of solvents, extraction techniques and clean-up approaches as recommended approaches of elution with only basic methanol will miss a range of important PFAS impacting both quantitative and qualitative analytical techniques. In the future, fully automated extraction approaches for biological (blood, tissue) and environmental samples (soil, water) will improve sensitivity, reproducibility, accuracy and precision for the quantification and identification of the PFAS at trace levels.

Fluorine mass balance is a powerful approach for evaluating how much organofluorine in a sample can be identified and quantified. Researchers have measured total organofluorine concentration using combustion ion chromatography (CIC) as an indicator of the presence or absence of PFAS and correlate this measurement with targeted quantitative approaches expressed as a fluorine equivalent [42]. Inductively coupled plasma TQ MS (ICP-TQ-MS) is emerging as a tool for the quantification of total fluorine in samples as a more robust, sensitive and quick technique for total fluorine compared to CIC [43]. When hyphenated with GC or LC, it may provide an unprecedented ability to identify novel PFAS in samples. Many PFAS contain non-fluorinated portions or oxidizable headgroups. Total oxidizable precursor (TOP) assay is a technique used to determine concentrations of oxidizable PFAS precursors (often volatile) in various environmental matrices [44]. The TOP assay is currently the only mainstream quantitative approach to assess the abundance of precursors without reference standards using LC-MS/MS methods [45]. Very few laboratories are equipped with facilities, resources or personnel to support complete PFAS characterization in environmental samples due to the multiple analytical techniques required.

### Concluding remarks

Given the ever-expanding catalogue of PFAS and their pervasive, complex behavior, environmental analytical chemists are crucial in tackling this issue. With increasingly stringent regulatory standards due to PFAS’s potential risks even at low concentrations, there is an urgent need for ongoing development of extraction and analysis methods. Such methods must achieve very low detection limits through advancements in LC-TQ and HRMS optimization, as well as extraction techniques. The omnipresence of PFAS in our homes leads to widespread bioaccumulation in populations worldwide. Research links PFAS exposure to various health impacts, including cancers, organ dysfunction, and reduced fertility [28]. The financial burden on communities and governments is monumental, potentially surpassing the global fluoropolymer industry’s annual value, estimated at $2 billion USD [46]. As we strive to mitigate these costs, the future of PFAS analytical chemistry will undoubtedly require innovative, cross-disciplinary approaches that enhance detection, understanding, and removal of these persistent contaminants.

## Acknowledgements

We gratefully acknowledge the support and collaboration of Agilent Technologies and Eurofins Australia for their support of the Australian Laboratory of Emerging Contaminants (ALEC). We also extend our gratitude to the University of Melbourne for their support and the provision of PhD scholarships. WA acknowledges Bisha University and the Saudi Education Ministry for their scholarship support. Some visualizations were created with BioRender.com.

## 5. Environmental monitoring strategies for PFAS

### Elsie M Sunderland^1^ and Bridger J Ruyle^2^

^1^ Harvard John A. Paulson School of Engineering and Applied Sciences, Cambridge, MA 02138, United States of America

^2^ Tandon School of Engineering, New York University, Brooklyn, NY 11201, United States of America

### Status

**Figure 6.**
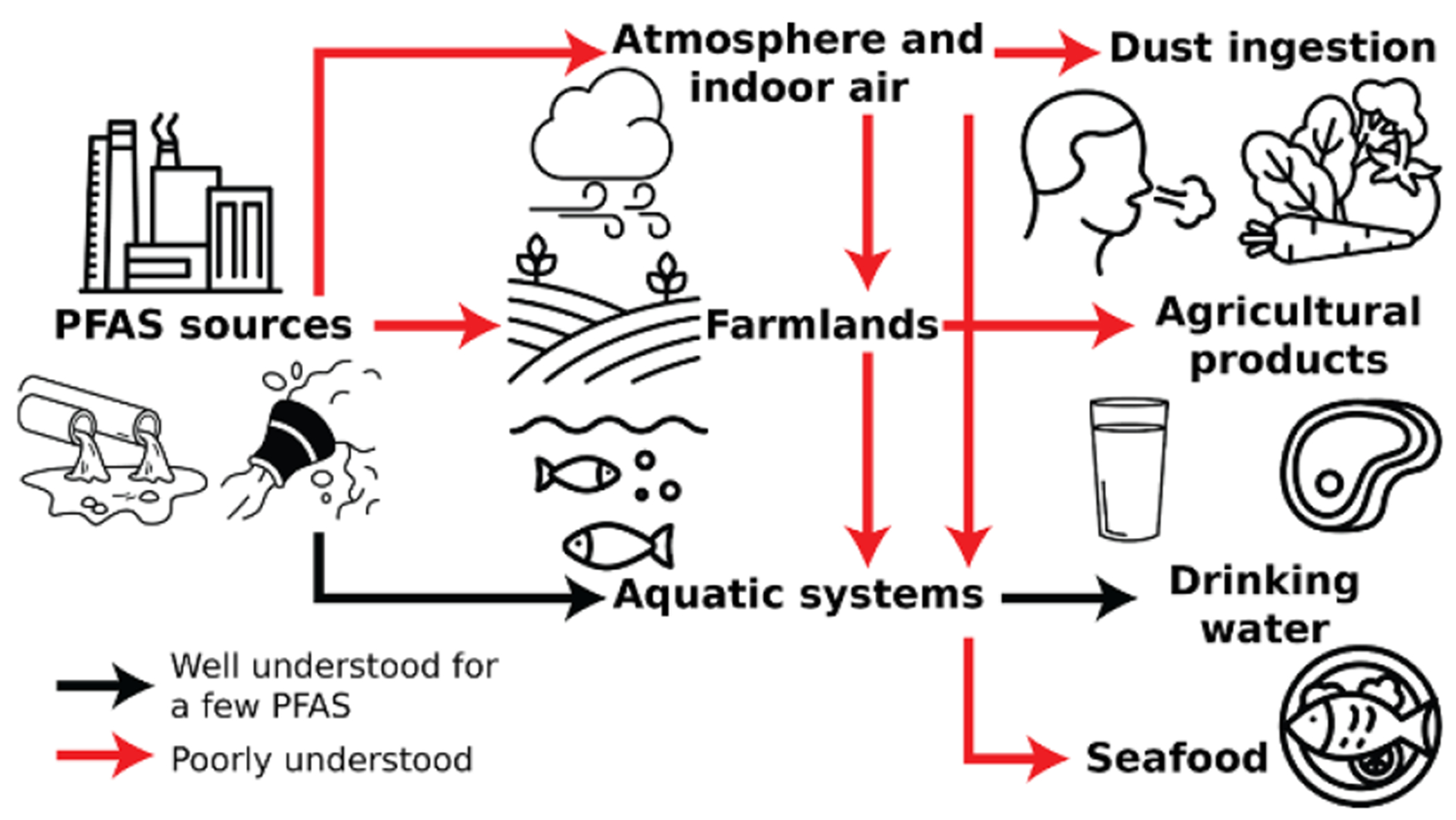
Schematic of human exposure sources coupled to environmental PFAS contamination. Data availability for each pathway is indicated by red arrows (poor data coverage) and black arrows (good data coverage). Specific data attributing the relative importance of each of these sources were reviewed in earlier work [51] but available data are not statistically representative of exposed populations and are extremely limited, emphasizing the importance of additional monitoring.

PFAS are a diverse class of highly fluorinated synthetic organic chemicals that are widely used in modern commerce and by industry [11, 47]. An alarming number of health effects have now been associated with exposures to some PFAS, including increased risks of certain cancers, metabolic and endocrine disruption, cardiovascular health risks, and immune dysfunction [48]. Ongoing animal studies (e.g. [49]) and toxicological assays (e.g. [50]) are elucidating mechanisms underlying some of these adverse outcomes, motivating the broad need to develop environmental monitoring programs that safeguard public health and wildlife from potentially adverse levels of PFAS exposures. However, both the breadth of PFAS exposure sources and range of individual analytes examined require expansion by future monitoring studies (figure 6).

Among environmental exposure sources, data on PFAS in surface and drinking water are most abundant, allowing important insights into population level exposure patterns [52, 53]. More limited data are available for assessing dietary PFAS exposure through agricultural products [54], seafood harvested from contaminated watersheds [55], and indoor environments [56], highlighting a need for expanded monitoring efforts. Research on PFAS fate and transport has largely overlooked atmospheric emissions and deposition as a major PFAS source [51]. New atmospheric monitoring efforts are therefore critical for understanding contributions of large atmospheric point sources to local deposition and global enrichment patterns resulting from atmospheric PFAS transport.

While thousands of PFAS are used in commerce [8], and hundreds have been detected at individual contaminated sites [36, 54], most monitoring programs have historically focused on the terminal PFAA with four to 11 perfluorinated carbons (C4-C11), especially perfluorooctane sulfonate (PFOS) and perfluorooctanoate (PFOA). Prioritizing other classes of compounds that share chemical properties, are especially abundant, and bioaccumulative would therefore be helpful. For example, perfluoroalkyl sulfonamido precursors, including the perfluoroalkyl sulfonamides (FASA), account for most of the PFAS burden in legacy aqueous film forming foams (AFFF) [57]. AFFF is the second largest source of drinking water contamination across the U.S. [58]. FASA are a concern in AFFF contaminated watersheds because they are semi-stable over long time periods and bioaccumulate by 1–3 orders of magnitude greater than PFOS, the main PFAS presently considered by fish advisory programs across the U.S. [59]. Prioritizing PFAS of concern is possible by identifying major contributors to the organofluorine mass budget using bulk organofluorine measurement techniques such as extractable organofluorine (EOF) [60], combined with targeted and non-targeted analyses, and the TOP assay [57, 59]. Such prioritization should consider available data on abundance, persistence, propensity for bioaccumulation and toxicity.

### Current and future challenges

Crafting effective risk mitigation advice for populations exposed to PFAS depends on an understanding of major exposure sources. Diverse PFAS exposure sources have been identified in the literature and the relative importance of each is expected to vary substantially depending on residential location, occupation, and lifestyle factors related to use of diverse products that contain PFAS [51]. However, quantification of the relative magnitudes of PFAS exposure sources for diverse populations is still nascent. Further, there is evidence that industrial PFAS sources are preferentially located in communities with higher proportions of people of color, exacerbating sociodemographic disparities in exposures [58].

Limitations in statistically representative data on PFAS exposure sources across multiple sociodemographic groups and geographic locations present a major challenge. Most PFAS exposure research to date has focused on drinking water, which accounts for the dominant fraction of exposure in contaminated communities, but typically <30% of total exposure in the general population with lower background levels of PFAS in drinking water [61]. Estimates of exposures to PFAS for different populations are often derived by highly uncertain ‘forward modeling’ of measured concentrations in each environmental media combined with an activity factor (e.g. PFAS concentration in seafood and amount of seafood consumed) rather than establishing constraints based on measured concentrations in human serum. Individual exposures to contaminated food from diverse sources are poorly understood, and only small ad hoc studies are available for relating the magnitudes of PFAS in indoor air and dust to serum PFAS concentrations [56].

Finally, our understanding of human exposure to different PFAS is limited by the suite of PFAS measured at a given time. Most consumer products and major sources like AFFF include diverse precursors to PFAA and/or other organofluorine compounds that cannot be detected using standardized targeted techniques [36, 54, 57, 60, 62] endorsed by government agencies (e.g. EPA Method 1633, table 2). Proprietary business information restrictions limit the availability of commercial standards needed to facilitate measurements. Overlooked PFAS such as FASA that are not included in routine monitoring programs [59] can sustain contamination of compounds of known health concern and may also convey direct exposure risks. Across a broad range of environmental media, PFAS mass budgets reveal that the concentrations of compounds found on typical analyte panels often fail to explain bulk organofluorine measurements such as EOF and ^19^F nuclear magnetic resonance (^19^F NMR) (table 2) [60].

### Advances in science and technology to meet challenges

Major advances in understanding PFAS exposure sources across diverse populations would be enabled by incorporating exposure assessments (both environmental and human measurements) into large scale epidemiological studies of PFAS health effects. The epidemiological literature on PFAS is rapidly expanding to include more diverse populations and regions [48]. Exposure research is interdisciplinary in nature and can effectively leverage basic health studies to include assessments that would better inform risk mitigation strategies essential for public health protection. Using PFAS composition in human serum to identify predominant exposure sources shows promise, especially if a total organofluorine measurement is available to derive quantitative source attribution estimates [63]. The detection limit for EOF samples varies across labs but is generally suitable for monitoring contaminated water, sediment, tissue, and human serum if a sufficient volume is available (typically at least 0.5 ml). For example, an interlaboratory comparison by Ruyle *et al* [64] reported a method detection limit between 0.68–2.18 *μ*g F L^−1^ for groundwater and 19–84 *μ*g F kg^−1^ for muscle tissue.

Elucidating the most abundant PFAS not presently included in routine measurements is essential for enhancing monitoring programs. Bulk organofluorine measurements such as EOF used in combination with HRMS, the TOP assay and targeted measurements can be useful for initial screening of the major groups of PFAS present and prioritization for future monitoring (table 2). However, such measurements are labor intensive and not currently suitable for routine monitoring programs. A promising next step in PFAS monitoring would be to develop characteristic PFAS mixtures associated with major source types that account for the majority of PFAS mass in exposure vectors. Such mixtures could be evaluated in toxicological assays and epidemiological health investigations to better link PFAS releases to impacts on organisms.

### Concluding remarks

Due to their persistence, PFAS are not known to fully degrade under normal environmental conditions. Therefore, the present-day burden of PFAS in the biosphere represents the cumulative sum of their production and use since their invention in the 1930s. Continued release of PFAS from legacy contamination sites and new production will further increase total exposures. Effective environmental monitoring programs that safeguard public health and wildlife from PFAS contamination must address the large data gaps on their prevalence across most environmental media and include a wider assessment of the most prevalent compounds [63].

## Acknowledgements

This work was supported by the National Institute of Environmental Health Sciences (NIEHS) under Award Number P42ES027706 and the U.S. National Science Foundation (Award No. 2108452).

## 6. Partitioning and fate

### Satoshi Endo^1^ and Trevor N Brown^2^

^1^ Health and Environmental Risk Division, National Institute for Environmental Studies (NIES), Onogawa 16–2, 305–8506 Tsukuba, Ibaraki, Japan

^2^ ARC Arnot Research & Consulting, Toronto, Ontario, Canada

### Status

**Figure 7.**
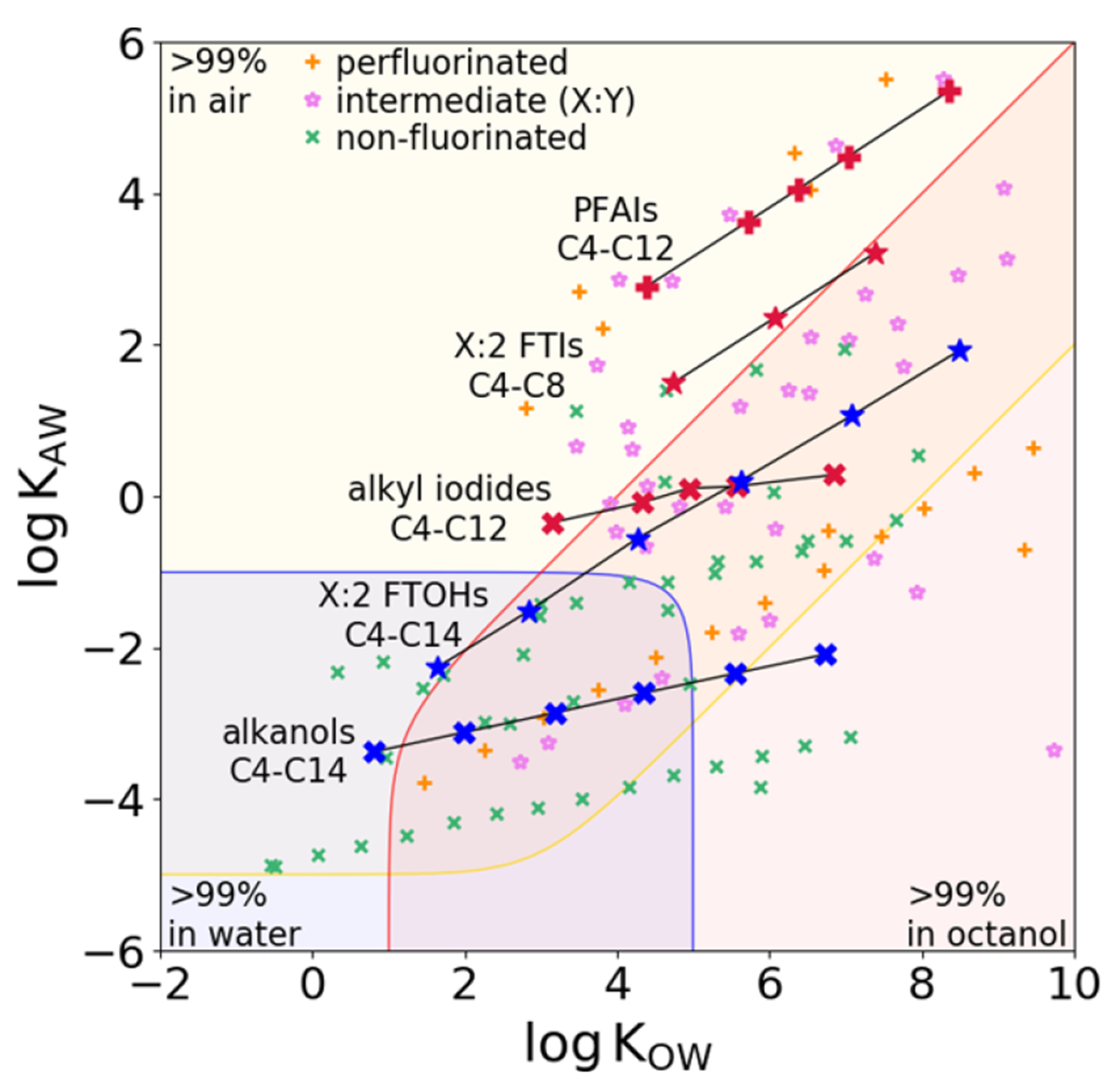
Chemical space plot of log *K*_AW_ vs. log *K*_OW_ of perfluorinated (orange crosses), partially fluorinated intermediate (purple stars) and non-fluorinated chemicals (green exes). Two chemical series are shown in more detail, alkanols (blue) and alkyl iodides (red) with and without fluorination. Partitioning data for PFAS are from [66] and for non-fluorinated chemicals the values are calculated by poly-parameter linear free energy relationships (PP-LFERs) using descriptors and equations from [67, 68].

Partition coefficients or ratios (*K*) between environmental phases such as air and water, natural organic matter and water, and biological lipids and water are key determinants of the fate and risk of environmental contaminants. PFAS, particularly, ‘highly fluorinated’ PFAS, e.g. with an F/C ratio >1.5 [65] or a perfluoroalkyl chain length of at least four [66] exhibit unique partition properties, distinctly different from nonfluorinated analogues. PFAS are generally more volatile and more hydrophobic than hydrocarbon analogues. Figure 7 shows the partitioning of neutral PFAS between air, water, and octanol, a common surrogate for organic phases, with a volume ratio of 10^6^:10^3^:1. Perfluorinated PFAS with F/C ratios typically ≥2 have higher log *K*_OW_ and much higher log *K*_AW_ values than their non-fluorinated analogues, with partially fluorinated PFAS in between. PFAS are not localized in a specific part of the chemical space like legacy contaminants such as PCBs, as fluorination only modifies the partitioning of the non-fluorinated analogues.

Partitioning of PFAS can be explained by two effects of the perfluoroalkyl group on the molecule. First, -CF_2_ - has a large volume compared to -CH_2_- but exerts only comparable van der Waals (vdW) interactions [65], meaning the vdW interactions of a perfluoroalkyl group are weaker than those of a nonfluorinated alkyl group of similar size. This large volume increases the cavity formation energy in any condensed phase, particularly in water, because water is a self-associating solvent and requires a high energy cost for cavity formation. This means that PFAS are more hydrophobic which increases the log *K*_AW_ considerably and the log *K*_OW_ to a lesser extent, as shown in figure 7, where increasing fluorination moves chemicals away from the aqueous portion of the chemical space. Second, the strong electron-withdrawing properties of the perfluoroalkyl group affect the polarity of the neighboring functional group [66]. For example, FTOHs shown in blue in figure 7 are stronger H-bond donors and weaker H-bond acceptors than their nonfluorinated counterparts. Since a higher H-bond acceptor property leads to a lower *K*_OW_, this also contributes to the increased hydrophobicity of neutral PFAS. Another prominent example of the electron-withdrawing effect is the low p*K*_a_ of perfluoroalkyl carboxylic acids (PFCAs), such as PFOA (p*K*_a_ ~ 0.5) [69], compared to alkyl carboxylic acids (p*K*_a_ ~ 5). The electron-withdrawing effect stabilizes the negative charge of the deprotonated carboxyl group and lowers the p*K*_a_ [70].

### Current and future challenges

While the mechanistic understanding has evolved as described above, data on *K*’s of PFAS are strikingly limited. Reliable experimental data for *K*_OW_ and *K*_AW_ were available only for a few neutral PFAS with 4 or more perfluorocarbon atoms until 10–20 new data were recently reported [66, 71]. One reason may be technical difficulties. Compounds with high volatility and low solubility are generally difficult to handle experimentally. Standard guidelines are often inadequate for high volatility, low solubility, and/or high surface activity. Increasing the number of replicates is insufficient because the error is systematic rather than random. Additionally, the prevalence of interfacial adsorption often confounds the measurement of bulk partition coefficients of C8 or longer PFAS [72, 73].

**Figure 8.**
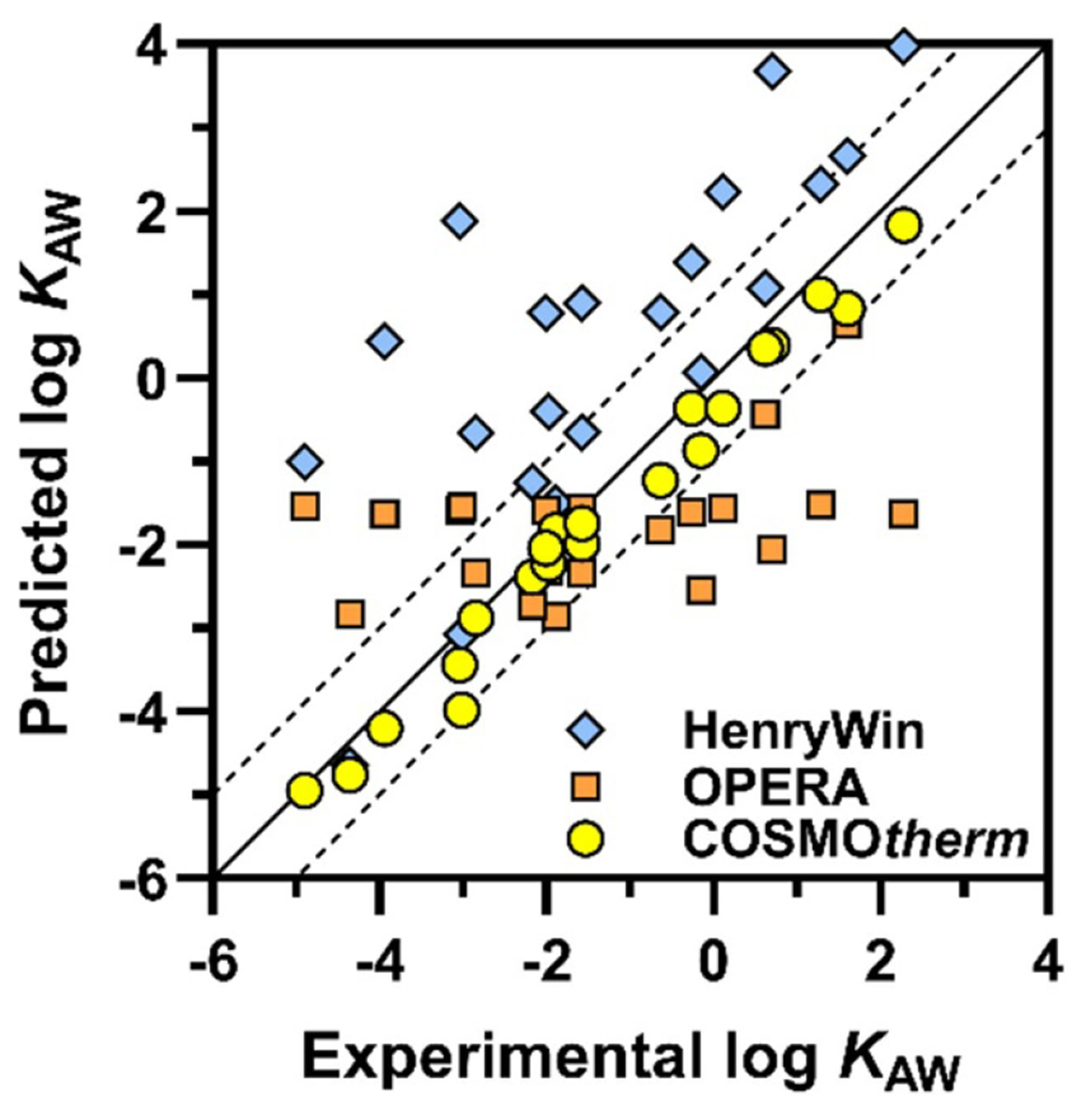
Prediction of log *K*_AW_ for neutral PFAS by HenryWin in EPISuite, OPERA, and COSMO*therm*. Data are from [71].

Quantitative structure property relationships (QSPRs) and empirical models such as PP-LFERs require reliable data for their calibration, and due to the lack of data PFAS are outside of the applicability domain (AD) of most calibrated models, e.g. see figure 8 and [68, 71]. The unique properties of PFAS, such as weak vdW relative to their size, make the AD even more problematic than it is for other data-poor chemical classes. For example, updating PP-LFERs to give accurate predictions for PFAS required not only the addition of solute descriptors but also a redefinition and recalibration of the equations, which worked for most other chemical classes [65, 66, 68]. Models that make predictions based on first principles, such as the quantum chemically based COSMO*therm*, have been shown to provide *K* values of PFAS with high accuracy, although they may not be freely available or may be difficult to use.

Understanding the partitioning of ionic PFAS is even more challenging. Ionic chemicals generally behave differently than neutral chemicals, being influenced more by pH and coexisting electrolytes in water and mineral components in soils and sediments. Nevertheless, a growing body of experimental data on relevant *K*’s for major ionic PFAS has been reported recently, including *K*’s from water to soils [74], the air/water interface [75], phospholipid membranes [76, 77], and serum proteins [50]. What is lacking is a general model for predicting these and other *K*’s for untested ionic PFAS and an understanding of the dependence of *K*’s on various environmental variables. For example, the quantum chemistry-based prediction algorithm COSMO*mic* can provide accurate predictions for nonfluorinated ionic compounds [78], but inconsistent predictions for PFAS [76], highlighting the need for further development.

### Advances in science and technology to meet challenges

Advances in measurement techniques and the generation of a reliable experimental data set of PFAS properties are the basis for an improved understanding of their partitioning and fate. Methods have been emerging to address known experimental artifacts, such as adsorption loss [50, 71], and must be further adapted for varying structure of neutral and ionic PFAS. High-throughput techniques are preferred, but accuracy should not be compromised.

Further experimental work on the partitioning of neutral PFAS will advance the development of calibrated prediction models such as QSPRs and PP-LFERs by providing more data to expand the AD and improve mechanistic understanding, such as the effects of branching and ether linkages on partitioning [68]. However, some classes of PFAS will remain experimentally inaccessible because of their ionic or surfactant properties, and a good combination of theory and data may be needed to extend the model AD while maintaining prediction accuracy. For example, combining experimental datasets with datasets of properties calculated with quantum chemistry methods might be used to expand the AD of models. So far, there is no theoretical framework to rigorously predict the partitioning properties of ionic PFAS. An extension to PP-LFERs for ions has been proposed; however it currently includes no PFAS in the training data [79], which represents a potential area for further development.

Finally, characteristic fate processes of PFAS should be linked to the PFAS physicochemical properties to understand which molecular features specifically lead to an unusual occurrence. For instance, the air-water interfacial adsorption process has been related to the foam separation technique [80], sea spray transport [81], and subsurface vadose zone transport [82]. Interfacial adsorption is more important for PFAS than for other chemicals, particularly at the air/water interface, due to the unfavorable partitioning of PFAS into bulk water. Simple correlation models have been proposed to predict air/water adsorption coefficients (e.g. [82]), which facilitate the interpretation of the atypical PFAS behavior. Models applicable to a broader range of PFAS will help generalize the relationship between physicochemical properties and environmental fate.

### Concluding remarks

The PFAS of concern to date have been those with a relatively high degree of fluorination. However, the current OECD definition of PFAS includes chemicals with even one -CF_2_-. In contrast, highly fluorinated chemicals without a perfluorinated carbon are not classified as PFAS but could be of similar environmental concern. An alternative definition of PFAS from Gaines *et al* includes the fraction of fluorine atoms in a chemical as an additional criterion to classify chemicals as PFAS [83]. Research on compounds with different degrees and patterns of fluorination is warranted to elucidate the fate properties of the full spectrum of organofluorine compounds. In addition, two strong research needs for PFAS partitioning are a better understanding of the partitioning of ions and surfactants. These topics are also the research needs for organic chemicals in general, so research in these areas should include a mix of fluorinated and non-fluorinated chemicals.

## Acknowledgements

Trevor N. Brown acknowledges ARC Arnot Research & Consulting for financial support for this work.

## 7. Adsorption and applications

### Zhengyang Wang^1,2^, Size Zheng^3^ and Joseph J Pignatello^4^

^1^ The New York State Center for Clean Water Technology, Stony Brook University, Stony Brook, NY 11794, United States of America

^2^ School of Marine and Atmospheric Sciences, Stony Brook University, Stony Brook, NY 11794, United States of America

^3^ Department of Chemistry, Stony Brook University, Stony Brook, NY 11794, United States of America

^4^ Department of Environmental Science and Forestry, The Connecticut Agricultural Experiment Station, New Haven, CT 06511, United States of America

### Status

Sorption is an effective approach for removing PFAS from water and soil. Sorption serves as a means of concentrating PFAS to make their destruction more feasible and economical. In batch-mode sorption, PFAS distribution between liquid and solid phases at (quasi)equilibrium is characterized by classical sorption models (e.g. Langmuir and Freundlich). In flow-through treatment units (e.g. adsorber) and *in situ* remediation, PFAS transport is predicted with mathematical advection-dispersion models. Sorption reactions are labile in real-world applications when environmental conditions change and/or when biological activity commences [84]. Recently, atomistic molecular dynamics simulations have provided valuable insights into atomic-scale details of adsorption on solid surfaces, achieving time resolutions from sub-nanoseconds to milliseconds [85, 86].

The discussion here particularly focuses on AC and anion exchange resin (AER) because currently they are the most widely applied and recommended by the U.S. EPA for PFAS removal in drinking water [87, 88]. In addition, AC has a potential for use in soil [89] and groundwater [90] remediation. New carbonaceous materials such as biochar, AC felts, and colloidal AC share similar sorption mechanisms with AC. Hydrophobic effects drive PFAS molecules out of aqueous solution [91] and on or into carbonaceous materials where the microenvironment is less hydrated. Given that most carbonaceous materials have a much higher anion- than cation-exchange capacity under ordinary environmental conditions, Wang *et al* [92] proposed an ion pair sorption mechanism to preserve charge neutrality in the solid phase (symbolized by ■) (equation (1)):

(1) $${{\text{PFAS}}^{-}}_{\text{aq}}+{{\text{M}}^{+}}_{\text{aq}}+■⇄\left({\text{PFAS}}^{-}{\text{M}}^{+}\right)\left(■\right),$$

where M^+^_aq_ is a cation in solution.

While AERs have different composition (e.g. polystyrene, polyacrylic), their sorption of PFAS is generally governed by electrostatic, hydrophobic, and vdW interactions [93]. Most PFAS are anionic, so anion exchange sorption is an important interaction:

(2) $${{\text{PFAS}}^{-}}_{\text{aq}}+\left({\text{A}}^{-}\right){(}^{+}■)⇄\left({\text{PFAS}}^{-}\right){(}^{+}■)+{{\text{A}}^{-}}_{\text{aq}},$$

where A^−^ is a counterion of the adsorbent.

The counterion is associated with a positive charge (symbolized by ^+^■) in the adsorbent. The reaction is driven to the right because PFAS is more hydrophobic and capable of vdW interactions than A^−^. Molecular dynamics simulations reveal that the predominant driving force for PFAS sorption is initially electrostatic attraction between the charged head of PFAS and the surface operating over a relatively long distance of >1.5 nm. Once PFAS molecules approach the surface, hydrophobic interactions between the fluoroalkyl tail and the hydrophobic domains of the material become dominant [94, 95].

### Current and future challenges

New PFAS regulations by the U.S. EPA set levels of 4 ng L^−1^ for PFOA and PFOS in drinking water. European Union regulations set levels of 500 ng L^−1^ for all PFAS and 100 ng L^−1^ for 20 PFAS. Compliance is technically and economically challenging but will benefit residents, stakeholders, and society.

The urgency in lowering PFAS level below the MCL calls upon more efforts to (1) improve the efficiency of current benchmark adsorbents, (2) revisit fundamentals of the sorption process, and (3) develop new adsorbents. Tailoring porosity [96] and surface chemistry [97] of adsorbents improves their sorption selectivity with respect to PFAS. In addition, *PFAS do not occur alone in environmental media.* Some chemical substances in surface water and groundwater, such as inorganic ions and dissolved organic matter (DOM), have much higher molar or mass-based concentrations than PFAS and may inhibit PFAS sorption and result in earlier PFAS breakthrough in treatment units [98].

Many studies have tested the effects of inorganic ions or DOM on PFAS sorption. For AER, the trend is consistent, as anions and DOM compete for anion-exchange sites. For carbonaceous materials, the trends concerning the effects of ions are contradictory. One reasons is that carbonaceous materials are prepared with different feedstock and, therefore, have different anion-exchange and cation-exchange capacities; future studies are suggested to report both for carbonaceous materials [99] because sorbed ions affect surface chemistry and then PFAS sorption. Nevertheless, most inorganic ions are far less hydrophobic than PFAS and will have a limited impact. DOM, which has a hydrophobic core and an amphiphilic exterior [100], will be more competitive. It also blocks pores in GAC, indicating that the size of DOM aggregates is relevant. The molecular weight distribution of DOM changes in response to variations in pH or ionic strength [101]. Although some studies have tested the effects of DOM, very few have investigated how DOM aggregation affects PFAS sorption.

The chain length effect is another significant factor in PFAS sorption. Short-chain and ultra-shortchain PFAS are more mobile in aqueous solutions, and, therefore, are less effectively removed than the long-chain homologues [102]. Due to competitive interactions between PFAS of varying lengths and different adsorbents, developing a universal adsorbent capable of removing PFAS across all chain lengths remains a challenge. While molecular dynamics simulations offer a powerful tool for investigating these complex interactions, the current lack of specialized force fields for diverse PFAS species poses a limitation. This gap in computational tools makes theoretical studies both resource-intensive and time-consuming.

### Advances in science and technology to meet challenges

In addition to conventional AC and AER, numerous novel adsorbents have been developed or are under development to sorb PFAS, including mineral-based and polymeric materials [103]. Polymer-coated, positively charged montmorillonite clay can effectively bind PFOS in simulated stormwater [104]. A new polymeric material, *ß*-cyclodextrin polymer, consists of monomers with hydrophobic cavities that trap PFAS molecules through host-guest complexation and has mesopores that enable rapid intraparticle diffusion [105]. A polymer (i.e. polypyrrole) coating strategy substantially improved sorption rate and density of short-chain and ultra-short-chain PFAS on pyrogenic carbons [106]. Some metal-organic frameworks have superior sorption capacity compared to GAC and AER but have not been tested at larger scales [107]. To determine capacity, PFAS concentrations in isotherm studies are often unrealistically high (mg L^−1^ levels). Evaluating novel materials in a flow-through mode with much lower concentrations (i.e. ng L^−1^ to tens of *μ*g L^−1^) is crucial because not all sorption sites are occupied by PFAS molecules in the real world where their influent concentrations are low. Meanwhile, applications of decision-making tools, such as techno-economic assessment, life cycle assessment, and multi-criteria decision analysis, can help evaluate the economic viability and sustainability of new adsorbents prior to scale-up [108].

Intraparticle diffusion is a rate-limiting step for porous adsorbents. Tailoring the porosity of GAC, AER, and other new adsorbents could facilitate intraparticle diffusion to expedite sorption. Additionally, treatment units operate in a diurnal mode, and sorbed molecules may migrate to the deeper sorption sites located in the interior porous areas during off-peak periods, when flow rates are slower and retention times are longer. Opportunities exist to engineer sorbate migration to inner sorption sites to create a sharper concentration gradient on and within a sorbent, benefiting mass transfer.

If PFAS can migrate between two adsorbents, then a fast-sorbing material could be *in situ* regenerated by a slow-sorbing material. Sorbate migration was successful when two iron-based carbonaceous materials were used for arsenate removal in an on-off pump cycling mode [109]. In general, no covalent bonds form when PFAS sorbs to GAC or AER, so PFAS would not experience the highly energetically unfavorable desorption that arsenate does. Small, novel materials might be mixed with granular materials (e.g. GAC) in a treatment unit for PFAS removal, with the latter potentially regenerating the former and then reshaping the mass transfer zone by redistributing PFAS.

Incineration is a valid approach to managing adsorbed PFAS and spent adsorbents. Other than incineration, salt aqueous solution combined with organic solvent can efficiently regenerate AER. The disposal of waste regeneration solutions is much less researched than the removal and regeneration of AER, albeit an important aspect of sustainable PFAS management. Additionally, low-cost calcium-based minerals can catalyze the thermal mineralization of PFOS that is laden on GAC, with the main end product being CaF_2_ [110], opening a new avenue to handle PFAS in spent adsorbents.

### Concluding remarks

Collective efforts in academia, industry, non-governmental organizations, and governments to resolve PFAS issues generate new knowledge in sorption and proof of concept for applications. They are beneficial for removing PFAS from water and managing PFAS in the environment. In this section, we weigh in on the application edge with arguments originating from the fundamentals of sorption. The effects of aging of adsorbents and environmental conditions are also important in research and development. In addition to identifying and removing PFAS in anthropogenic water cycles, PFAS transport in the environment is equally important, and sorption is a key step in affecting PFAS transportation. Attention should also be paid to the disposal of spent adsorbents applied for PFAS removal, given the enormous, estimated amount of 200 000 wet tons per year of spent GAC and AER generated in the U.S. [111]. Efforts are needed to extract PFAS from spent adsorbents and/or find ways to destroy the sorbed PFAS directly, such as enhanced thermal mineralization. Taken together, sorption will become an invaluable component for successful PFAS management, and enhancing sorptive removal with GAC, AER, and other novel sorbents will be a prerequisite for the efficient destruction of PFAS.

## Acknowledgements

Support from the New York State Department of Health through a Grant (No. 1191215) to the New York State Center for Clean Water Technology (NYS CCWT) is acknowledged. Discussions on sorption mechanisms with Dr Yi Zhang and Dr Sheng Yin at NYS CCWT were helpful.

## 8. Enrichment techniques

### Sanne J Smith^1^, Marcel Riegel^2^ and Hans Peter H Arp^3,4^

^1^ Department of Water Management, Delft University of Technology, Stevinweg 1, 2628 CN Delft, The Netherlands

^2^ TZW: DVGW-Technologiezentrum Wasser, Karlsruher Str. 84, 76139, Karlsruhe, Germany

^3^ Environmental Technology, Norwegian Geotechnical Institute (NGI), Oslo NO-0806, Norway

^4^ Department of Chemistry, Norwegian University of Science and Technology (NTNU), Høgskoleringen 5, 7491 Trondheim, Norway

### Status

**Figure 9.**
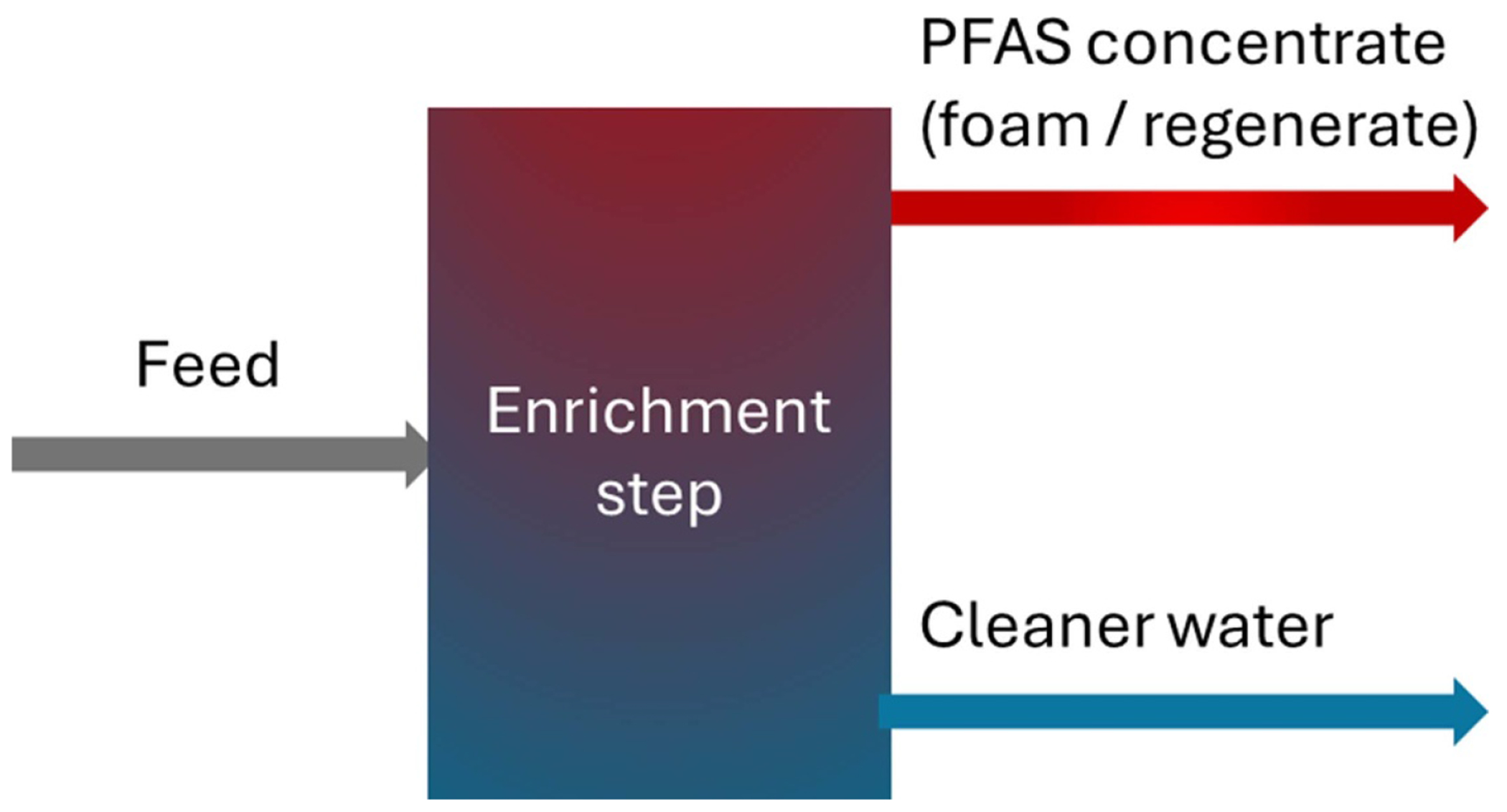
An enrichment step within a water treatment trains that isolates a PFAS concentrate from the feed water for separate handling or management.

Enrichment techniques can be utilized as part of treatment trains within advanced water treatment facilities to decrease the cost and energy consumption of PFAS remediation and removal methods (figure 9). As the name implies, enrichment techniques enrich—or up concentrate—PFAS into a separate aqueous phase from the bulk water, which can then be further managed separately as much smaller volume. In contrast to adsorption processes such as AC or single-use ion exchangers (see section 7), the PFAS are not enriched in a solid phase but in a liquid phase. A common way to characterize the effectiveness of an enrichment process is the VRF, which is defined as the ratio of the initial volume of water being treated divided by the volume of PFAS enriched solution that is retained. For instance, an enriched PFAS liquid phase with a higher VRF is more suitable for further processing by an energy-intensive destruction technology (incineration, EO, supercritical fluid oxidation, etc), either as a low-volume concentrate, or even as crystalline salts in the case of thermally evaporated brines.

### Current and future challenges

#### Membrane processes

A widely used enrichment method is filtration via high-pressure membranes, such as nanofiltration (NF) and reverse osmosis (RO). Here, feed water is pumped under high pressure through membranes with effective pore sizes between 1 and 10 nm (NF) or <1 nm (RO). Due to size exclusion and/or electrostatic repulsion, PFAS with a molecular weight above approximately 200–1000 Da (NF) or 100 Da (RO) are retained on the feed side of the membrane, and remain in the waste stream called ‘concentrate’ [112]. In contrast, the ‘permeate’ that has passed through the membrane is relatively PFAS-free, with PFAS rejections of >95% for NF and >99% for RO. Typically, high-pressure membranes achieve water recoveries of 70%–90%, i.e. VRFs between 3–10. Recovery is limited by the quality of the feed water, with cleaner feed waters enabling higher water recoveries and, equivalently, higher VRFs. Therefore, high-pressure membrane processes are mostly applied for the treatment of relatively clean water matrices, such as drinking water or groundwater. We estimate that the investment and operating costs for a membrane plant operating at full flow without a bypass would be at a total of 0.5–0.7 € m^−3^.

#### Foam fractionation

Foam fractionation is a separation technology that exploits the surface-active properties of many non-polymeric PFAS, particularly longer chain PFAAs and their precursors [113]. When air bubbles are introduced to water, a large air/water interfacial area is created, to which these surface-active PFAS adsorb. The adsorbed PFAS rise with the bubbles through the water and accumulate in a foam layer on top of the water. Depending on the water matrix, it may be necessary to dose additional surfactants, to create a sufficiently stable and high foam layer [114]. Separation of this foam results in separation of the PFAS from the water, thereby creating a relatively PFAS-free effluent. Unlike conventional flotation processes, including dissolved air flotation, foam fractionation removes dissolved molecules instead of suspended particles. The energy requirement of foam fractionation (<1 kWh m^−3^) is typically <10-fold lower than that of microbubble flotation, because the introduction of larger bubbles diminishes the energy use [115].

Foam fractionation is already applied in full scale for PFAS removal from a variety of water matrices, such as groundwater and landfill leachate [116, 117]. It can be applied in batch as well as continuous mode. Important process variables are the retention time of the water, the air flow rate and the height of the foam layer [114, 118]. It can be operated in multiple stages to further reduce the volume of PFAS-rich foam, with two to three stages being employed in most commercial systems [116, 117].

The main benefit of foam fractionation is its relative simplicity compared to other enrichment technologies: no chemicals are required except air (and sometimes surfactants), its performance does not diminish in heavy-matrix water, and it has a relatively low energy demand [113]. Additionally, the technology can achieve very high VRFs of >50 already in the first stage [119, 120], which can be increased even further in second and third stages of foam fractionation. These benefits make the technology highly suitable for the treatment of heavily contaminated matrices, such as landfill leachate, industrial effluents, AFFF-contaminated groundwaters or RO concentrates.

#### Regenerative IEX processes

Another technology for PFAS enrichment is the use of ion exchangers (IEX) if a regeneration step is included to remove the sorbed PFAS from the IEX resin into a brine. Ion exchangers are polymeric grains that contain a charged functional group to be able to bind different dissolved ions. For PFAS removal, AER are applied in filter columns similar to the use of GAC. PFAS adsorb to anion exchangers due to electrostatic interactions (perfluorocarboxylic acids and sulfonic acids are negatively charged at neutral pH) and vdW interactions of the hydrophobic alkyl chain [93, 121]. A certain operating time is observed before the breakthrough of the substance occurs. Similar to the use of GAC and foam fractionation, long-chain PFAS are also better adsorbed than short-chain PFAS when using ion exchangers [122]. Strong basic anion exchangers show better adsorption performance than weak basic ones [122]. In addition, perfluorinated alkyl sulfonates are better adsorbed than perfluorinated carboxylic acids [123]. A limitation of IEX is fouling or exhaustion of the sorption sites on the resin. Due to competitive sorption, increasing amounts of sulfate will result in reduced PFAS uptake and shorter operating times to breakthrough [124]. All anion exchangers show faster adsorption kinetics compared to AC filtration, resulting in smaller filter vessels and therefore lower investment costs (in relation to the treated water volume of approx. 0,05 € m^−3^ compared to approx. 0,1 € m^−3^) [125]. However, the material prices of IEX are significantly higher than those of GAC (by a factor 5–10). Therefore, many regeneration and loading cycles must be achieved to realize economic benefits.

During regeneration, the functional groups of the ion exchange resin are returned to their original form and another cycle of PFAS removal can be performed. However, due to the additional vdW interactions, regeneration does not work well for long-chain PFAS elution. With standard regenerants such as sodium hydroxide or sodium chloride solutions, only poorly adsorbable PFAS such as short-chain carboxylic acids can be eluted and, in general, the regeneration works better with weakly basic than with strongly basic anion exchangers. Elution can be greatly improved by using regenerates in combination with organic solvents such as methanol or ethanol [126]. However, the use of organic solvents is undesirable and impractical, especially in drinking water treatment.

### Advances in science and technology to meet challenges

#### Membrane processes

Fouling is one of the major challenges in the application of membrane technologies. Accumulation of organics, salts, or microbial growth on the membranes can cause a loss of water flux through the membranes, which needs to be addressed with adequate cleaning protocols. Other challenges include the relatively low water recoveries/VRFs, and a lower rejection of short-chain than long-chain PFAS. Advances in material science engineering of membrane materials can address all these challenges, and membranes designed specifically for the removal of PFAS are under development [127].

#### Foam fractionation

Foam fractionation at scale can currently only remove longer-chain PFAS, since short-chain PFAS adsorb less strong to air-water interfaces [118]. However, recent papers show promising results when cationic surfactants are used as additives, facilitating PFBA removal to below LOQ (50 ng L^−1^) in artificial solutions [119, 120]. Commercial suppliers of foam fractionation systems are starting to include these additives in their systems. Additionally, to overcome mass losses to the surrounding atmosphere, installation of appropriate air filters is becoming more common to prevent PFAS emissions via the air exiting the reactor [128]. Foam fractionation is not yet capable of achieving similarly low effluent concentrations (<ng L^−1^) as membranes, GAC or IEX processes, which may also be improved by using innovative additives.

#### IEX processes

Due to the poor regenerability of PFAS on IEX processes, a new approach is being tested in the EU ZeroPM project [129]. An AC filter is installed upstream of the ion exchanger to minimize the amount of long-chain PFAS entering the ion exchanger. A weakly basic anion exchanger is used as a second treatment step. This has shorter operating times until regeneration, but a higher proportion of the adsorbed PFAS is eluted during regeneration. With caustic soda, VRFs of 250 and 750 have been achieved for short-chain PFAS such as PFBA and PFPeA. Compared to the standalone use of GAC, economic advantages can be achieved here if the GAC has very short run times due to high raw water concentrations of short-chain PFAS such as PFBA (where operating times of 5,000 BV were achieved until PFBA could no longer be retained). Additionally, 15 loading cycles were run without any noticeable loss of capacity with the help of regenerations with caustic soda. However, these regenerable, weakly basic ion exchangers are very expensive at an estimated 17 € L^−1^. Therefore, considerable research and development is still required to make the technology more economical.

### Concluding remarks

A general limitation of enrichment technologies is that the shorter the PFAS, the less effective the methods. However, optimization can be done in this direction. A promising option is to install foam fractionation upstream from a regenerative IEX process, with the foam fractionation removing longer-chain PFAS (or potentially shorter ones by use of surfactants), which could then be followed by regenerative IEX to target the shorter chain PFAS. Filters or GAC could be added to such a treatment train to prevent IEX fouling.

A key consideration is what ultimately happens to the PFAS-rich concentrate produced by enrichment processes. It is highly recommended that enrichment techniques are designed alongside PFAS destruction methods that verify full PFAS mineralization using a fluorine mass balance [130], or alongside long-term storage options. sections 9–14 give an overview of promising destruction technologies that are currently under development.

## Acknowledgements

Funding is acknowledged from the European Union’s Horizon 2020 research and innovation programme under Grant Agreement No. 101036756 (ZeroPM)

## 9. Hazardous waste incineration

### Jens Blotevogel^1^, Robert J Giraud^2^ and Anthony K Rappé^3^

^1^ Commonwealth Scientific and Industrial Research Organisation (Waite Campus), Urrbrae, SA 5064, Australia

^2^ Department of Chemical and Biomolecular Engineering, University of Delaware, Newark, DE 19716, United States of America

^3^ Department of Chemistry, Colorado State University, Fort Collins, CO 80523, United States of America

### Status

Incineration of solid and liquid industrial wastes is a mature technology that can destroy organic compounds and reduce landfill disposal. A typical hazardous waste incinerator consists of:

- a rotary kiln primary combustion chamber, in which organic chemicals volatilize and start to degrade,
- a secondary combustion chamber (SCC) designed to fully mineralize volatilized organics,
- ash/slag collection, and
- an air pollution control system that cools the gas, removes particulates and acid gases, and releases the exhaust gas through a stack.

**Figure 10.**
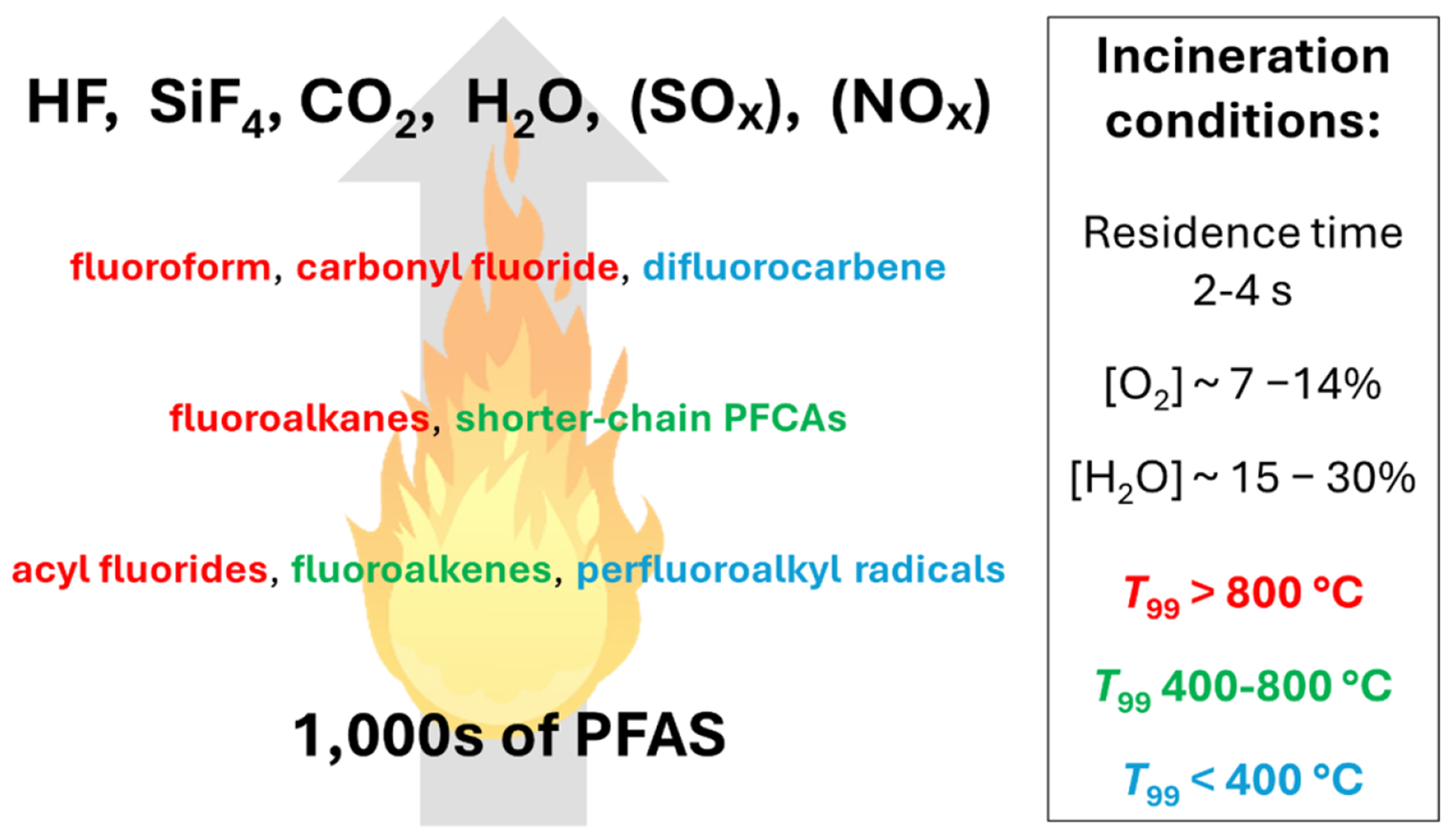
Conceptual overview of PFAS destruction pathways during hazardous waste incineration. Key intermediates and intermediate classes are color-coded by T_99_ range under typical incineration conditions in the secondary combustion chamber (a.k.a. afterburner) of a hazardous waste incinerator. T_99_ is a common laboratory benchmark for comparing the incinerability of organic compounds, indicative of the temperature needed for 99.99% destruction and removal efficiency at full scale [18]. While the family of PFAS possess great chemical diversity with thousands of individual species, their complexity during thermal destruction progressively decreases until mineralization is complete.

PFAS were originally designed to withstand high temperatures, such as in the application of AFFFs. Nevertheless, recent research has demonstrated that their complete mineralization to HF, CO_2_, and H_2_O is feasible at temperatures below those required for several other organic chemicals that are incinerated on a regular basis [131, 132]. Both computational and physical studies up to the pilot scale have shown that the great chemical diversity of PFAS is successively reduced to a few key intermediates during incineration (figure 10) [132, 133]. The destruction of fluoroalkane intermediates, such as 1*H*-perfluoroalkanes, was identified as the kinetically limiting step. It was estimated that during 2–4 s of gas residence time and temperatures of ≥1000 °C, which are typical for SCCs in hazardous waste incinerators [134], several orders of magnitude in PFAS mineralization can be achieved [132].

However, adverse process conditions such as poor mixing or high free fluorine concentrations have the potential to facilitate the generation of products of incomplete combustion (PICs). Some experimental studies have observed the formation of tetrafluoromethane [135, 136]. This potent GHG requires incineration temperatures >1400 °C [131]. Optimizing the incineration process to avoid CF_4_ formation should therefore be a primary goal.

Sampling and analytical methods for the identification of fluorinated PICs in the gas phase, such as U.S. EPA Other Test Method 55 (OTM-55), are only now being developed and are not widely available yet. Knowledge gaps around stack emissions have therefore led to a moratorium on PFAS incineration for the U.S. Department of Defense, which currently stockpiles many PFAS-containing materials, such as AFFFs [132]. Other countries such as Germany rely on incineration of PFAS-impacted wastes such as biosolids to minimize application to agricultural lands. Clearly, more research and guidance on the fate of PFAS during hazardous waste incineration are urgently needed.

### Current and future challenges

Much of the existing concern about PIC emissions from PFAS incineration is rooted in the lack of adequate analytical methods. For destructive technologies, closing the fluorine mass balance is the goal [130] but poses a significant challenge in both full- and bench-scale systems. Identification and quantification of every individual PFAS in all residues and phases is not only impractical but also impossible because calibration standards are not commercially available for many key intermediates. Currently available total fluorine methods suffer from insufficient sensitivity, lack of calibration standards, and other method-specific limitations [137]. In addition, HF interacts with the incinerator surface to produce volatile Si–F reaction products (e.g. SiF_4_) that may not be captured by conventional exhaust gas sampling and analysis techniques.

Even more challenging is the qualitative and quantitative analysis of radical intermediates. However, their evolution, nature, and fate are key to optimizing destruction efficiencies while minimizing recombination reactions that may lead to PIC formation. A first report of direct measurement of fluorocarbon radicals during thermal PFAS destruction has only just emerged [138]. Several decomposition reactions, such as H abstraction and OH addition, as well as recombination reactions, such as •CF_3_ + •F → CF_4_, are bimolecular and therefore follow concentration-dependent second-order kinetics [131]. The reaction atmosphere in full scale incinerators is poorly understood and concentrations of key reactive species, such as radicals, water, fuel and other fluorine acceptors, are largely unknown. A predictive understanding is further complicated by variable waste stream composition affecting chemical concentrations and imperfect mixing leading to inhomogeneous chemical, temperature, and residence time distributions [131].

Similarly, the role of surface reactions is poorly understood. It has long been recognized that surfaces may alter kinetics and decomposition pathways during thermal treatment of PFAS [139]. Recently, pilot-scale studies have measured SiF_4_ and other Si–F compounds quantifying the impact of fluoride reactions with incinerator refractory surfaces and raised questions about time-dependent deposition of thermal PFAS destruction products on these surfaces [133, 140]. Relevant surfaces are not limited to the incinerator walls but also include for instance fly ash and slag formers in the primary combustion chamber, for which additional experimental insights are needed. It is worth considering that there are other thermal treatment approaches that at least in part harness surface catalytic processes, such as reactivation of PFAS-laden AC, during which PFAS may start to decompose at temperatures as low as 200 °C [141]. It is yet unknown whether the generated highly volatile intermediates keep undergoing catalytic breakdown at the surface or volatilize, in which case they would require much higher temperatures for complete mineralization.

### Advances in science and technology to meet challenges

As advanced sampling and measurement methods are becoming available, such as OTM-50 and soon OTM-55, additional efforts in analyzing the intermediates of PFAS incineration are required. Non-targeted high-resolution MS will help identify stable PICs and inform synthesis of additional calibration standards. Synchrotron-based molecular-beam MS may be applied to detect short-lived radical intermediates [138]. Real-time analysis, for instance via inline Fourier-transform infrared spectroscopy [142] or fluorocarbon compound-specific (e.g. CF_4_) lasers, would be of tremendous value both at the bench and at the field scale. Note that MS-based methods such as OTM-45, –50, and –55 can detect analytes at parts-per-trillion or low parts-per-billion levels, while spectroscopic and synchrotron-based methods require higher parts-per-million or even % concentrations.

Improved chemical analyses can then support well-designed bench-scale experiments that properly simulate the essential high-temperature chemistry affecting transformations in the combustion zone of full scale incinerators. These small-scale experiments are critical to understanding the emission impact of specific materials without the influence of variable waste feed composition encountered at full scale [143]. Physical experiments should be supported by *ab initio* computational chemistry methods that assist in identifying underlying decomposition, recombination and catalytic mechanisms, driving a deeper understanding of kinetic bottleneck reactions in high-temperature incineration and other thermal PFAS treatment technologies [132].

Tetrafluoromethane is the most thermally stable fluorocarbon PIC because only cleavage of the strong C–F bond leads to its decomposition [144]. It is therefore necessary to thoroughly understand the mechanisms of CF_4_ formation and prevention in practical incineration systems, determining the role of key operating parameters related to the availability of constituents such as water, hydrocarbons (fuel), hydrogen, and fluorine acceptors (e.g. lime) under relevant thermal treatment conditions. Other stable PICs that require higher temperatures (>800 *^◦^*C) include, but may not be limited to, (hydro)fluoroalkanes and -alkenes, acyl fluorides, and carbonyl fluoride (figure 10) [132]. Processes need to be optimized to ensure complete mineralization of these kinetic bottlenecks as they are potent GHGs and may be toxic, such as carbonyl fluoride.

Ultimately, full scale testing at hazardous waste incinerators is inevitable to validate mechanistic findings at smaller scales. Trials should focus on incinerating wastes of different composition, such as PFAS-impacted soils, wastes and (dilute) AFFFs, determining the role of key operating parameters, such as fuel:oxygen:PFAS ratio and lime addition, and elucidating the effectiveness of air pollution control system in preventing dibenzodioxin and -furan formation and reducing PFAS air emissions [145].

Finally, the coupling of kinetic modeling with computational fluid dynamics will be a worthwhile undertaking to investigate the effects of local process conditions on PIC formation in order to optimize mixing and reduce fluorocarbon emissions [146].

### Concluding remarks

Contaminant destruction is preferred over landfilling to minimize future legacy pollution. Several destructive PFAS technologies are being developed but only few are available commercially [147]. To treat all the PFAS-containing wastes that are being generated globally, hazardous waste incineration is a critically needed tool. While more research is needed to optimize and safeguard incineration processes, research conducted thus far suggests that complete thermal destruction of PFAS should be viable as long as best practices are followed. The same may not be true for other thermal treatment technologies with lower temperatures and/or shorter residence times, for which our understanding of gas-phase reactions is still evolving. Significant investments into research as well as reestablishment of long-term training and research programs at academic institutions to prepare the next generation of engineers and scientists are critically needed to ensure the safe destruction of PFAS and other POPs in hazardous waste incinerators.

## Acknowledgements

Financial support for this research was provided by the U.S. Department of Defense’s Strategic Environmental Research and Development Program (SERDP) under Projects ER21–1019 and ER24–4073.

## 10. Pyrolysis and biochar for PFAS management

### Erlend Sørmo^1^, Gerard Cornelissen^1,4^ and Hans Peter H Arp^2,3^

^1^ Faculty of Environmental Sciences and Natural Resource Management (MINA), Norwegian University of Life Sciences (NMBU), Elizabeth Stephansensvei 31, 1433 Ås, Norway

^2^ Environmental Technology, Norwegian Geotechnical Institute (NGI), Sandakerveien 140, 0484 Oslo, Norway

^3^ Department of Chemistry, Norwegian University of Science and Technology (NTNU), Høgskoleringen 5, 7491 Trondheim, Norway

^4^ Norwegian Institute for Bioeconomy Research (NIBIO), Oluf Thesens vei 43, 1433 Ås, Norway

### Status

**Figure 11.**
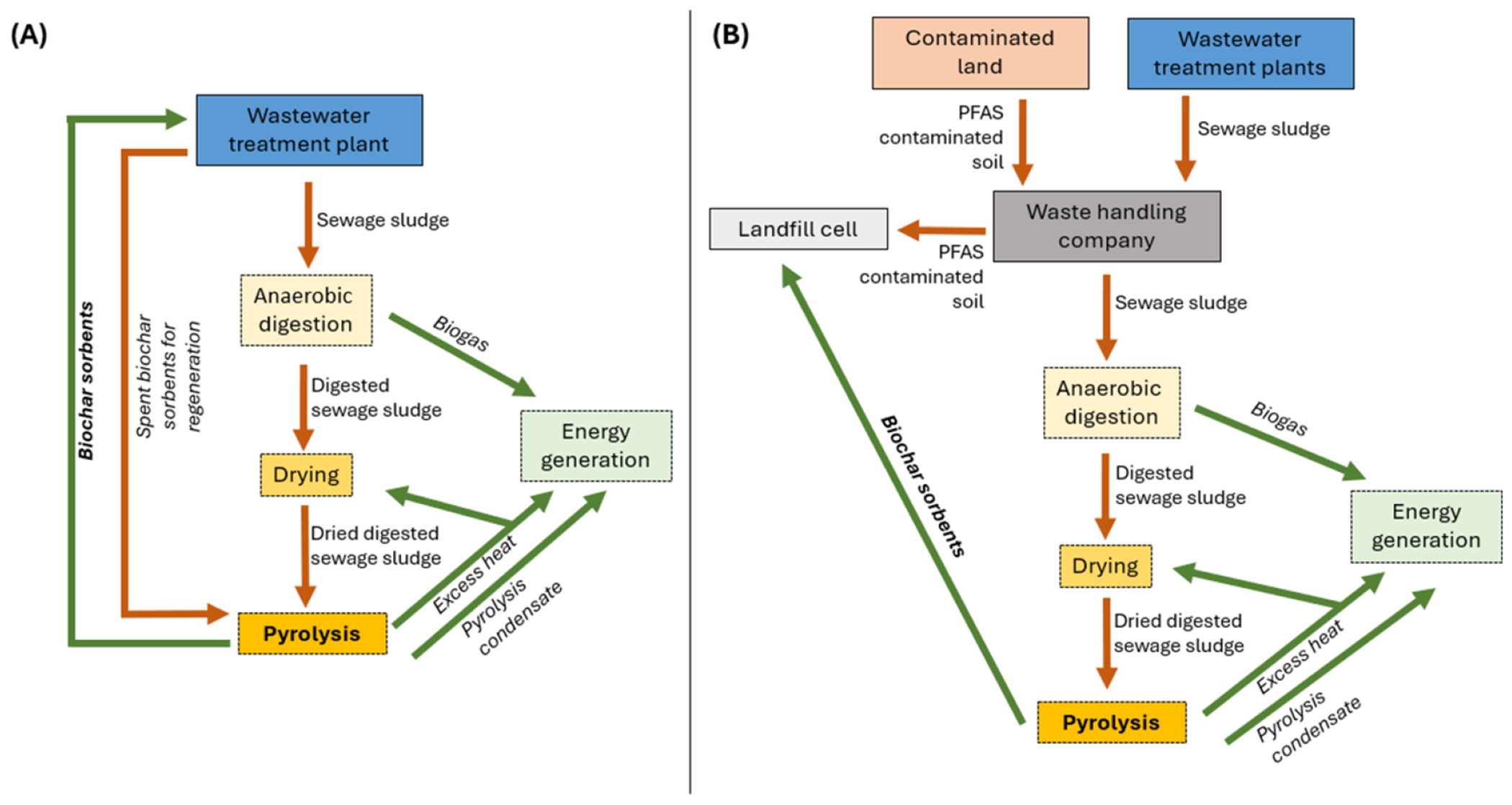
Potential options for integration of pyrolysis into organic waste handling at a wastewater treatment plant (A) and a waste handling company (B). Other integrations are also possible, such as PFAS contaminated paper sludge being pyrolyzed to make a biochar sorbent for effluent water.

Pyrolysis of PFAS-contaminated organic waste, combined with using the generated ‘waste biochar’ as sorbents for PFAS remediation, offers great potential for future environmental management in diverse sectors (figure 11).

Pyrolysis is a waste handling option that entails heating in an oxygen-depleted atmosphere. It is an emerging strategy for sustainable organic waste handling with value creation potential [148]. Pyrolysis produces energy-rich syngas for energy generation, and solid biochar with multiple applications in soil/water PFAS remediation and soil restoration [148, 149].

Pyrolysis can be contrasted with current waste incineration systems that often require expensive, large-scale infrastructure [150]. Pyrolysis can be done at smaller scales. Biochar, unlike sludge, is not readily mineralized into CO_2_ when placed in the environment, rendering it a sustainable, carbon-sequestering treatment alternative for contaminated organic wastes [150–153]. Pyrolysis and incineration temperatures ≥500 °C are sufficient to remove various persistent organic contaminants [150], though for PFAS higher temperatures are likely needed for full destruction [110, 151]. Nevertheless, Morales *et al* [150], considering CO_2_-emissions, contaminant removal and ecotoxicological impacts, showed that pyrolysis for sewage sludge treatment has a lower environmental life cycle impact than incineration.

Apart from carbon storage, biochar offers a range of benefits, such as soil quality improvement through alleviation of acidity and increased water and nutrient retention [148]. Biochar shares characteristics, such as a high carbon contents (>50%) and large specific surface area (SSA, 200–1000 m^2^ g^−1^), with the AC sorbents produced from lignite coal [149, 154]. Biochar has thus emerged as a sustainable sorbent option replacing AC [148, 149]. A decade of studying biochar as a PFAS sorbent in soil and water has recently been reviewed [154].

As biochar effect on PFAS sorption vary [154] while AC is readily available commercially with predictable effect, it is not expected that biochar can compete economically in the short term. Integrating pyrolysis in waste management for biochar production (figure 11) is, however, expected to reduce overall environmental impact and generate added value through it providing a waste-management strategy, thus making biochar a competitive alternative in the long term.

### Current and future challenges

#### Pyrolysis

Laboratory studies have demonstrated PFAS volatilization and varying degrees of thermal degradation upon pyrolysis between 200 °C–900 °C [110, 141, 152, 155]. Several larger-scale studies of PFAS fate during sewage sludge pyrolysis have reported biochar products with low to non-detectable PFAS [151–153]. Long chain PFAS are more likely to remain in the biochar compared to short chain PFAS, which are more easily volatilized into the pyrolysis gas [151, 152]. PFAS volatilized to the pyrolysis gas can accumulate in the pyrolysis condensate [152].

Besides volatilization, there is evidence of partial degradation of PFAS during pyrolysis. A net increase in the mass of short chain PFAS has been observed in pyrolysis condensate from sewage sludge [152]. A whole suite of volatile degradation products, such as perfluorinated alkanes, alkenes and alcohols, have been identified in pyrolysis gas from small lab-scale reactors [141, 155]. In full scale systems further degradation likely occurs, as the pyrolysis gas is combusted at 800 °C–1000 °C [150, 151]. PFAS emission data from such systems are, however, scarce. Sørmo *et al* [151] recorded emissions of 0.01–3.1 mg tonne^−1^ of biochar produced, dominated by short chain PFAS and accounting for <3% of the total target PFAS in the feedstocks. There is a strong need for more emission data, particularly nonexistent information on volatile decomposition products, such as perfluorinated GHGs like fluoroform.

#### Biochar

PFAS can sorb extremely strongly to biochar, with PFOS log *K*D values up to 6.3, which is 1000–5000 times higher than typical *K*_OC_ values [156]. PFAS mainly adsorb to biochar surfaces through hydrophobic interactions, as evidenced by positive correlations between sorbent performance and fluorocarbon chain length [156–159] and the degree of biochar hydrophobicity [160]. For ionic PFAS, electrostatic effects also play a significant role, as the head group can interact with charged sites on the biochar surface, but this role is more notable for ultra-short chain PFAS [157]. Biochar is alkaline and its surfaces carry a net negative charge [149]. This could potentially result in electrostatic repulsion between anionic head groups and the biochar surface. This weakens the net sorption and explains why biochars generally do not sorb short-chain anionic PFAS as well as other PFAS [154, 156, 157]. Positively charged functional groups or mineral surfaces integrated in the biochar structure can increase the sorption potential through electrostatic attractions [154].

Biochar sorption capacity is regulated by pore-filling capacity, for which SSA and pore volume (PV) are strong indicators [149]. However, pores need to be sufficiently large to accommodate PFAS molecules to avoid steric hinderances and restricted diffusion [156]. The ideal biochar pore size for PFAS immobilization is probably a minimum of twice the molecular diameter [156].

Uncertainties still exist regarding the longevity of biochar remediation of PFAS-contaminated soil [161]. Nevertheless, complete desorption is unlikely for PFAS [160], for several reasons: (i) the biochar is extremely stable in soil over thousands of years [148], (ii) over time the PFAS diffuse more deeply into the biochar matrix, and (iii) biochar particles get increasingly incorporated into soil aggregates over time [162]. Long-term field studies are, however, recommended, to validate these claims in diverse biocharsoil systems.

### Advances in science and technology to meet challenges

#### Pyrolysis

Closing the fluorine mass balance in full scale pyrolysis systems [141, 150–152]. can be achieved with more target analytes, non-target screening, and total EOF analyses. It also requires sampling of all relevant fractions: feedstock, biochar, condensate and pyrolysis gas. Standardized extraction methods are, furthermore, needed for strongly sorbing matrices such as biochars and pyrolysis condensates. Insufficient extraction protocols can result in false negatives and jeopardize mass balance estimates [151]. Improved methods are being developed for vapor-phase/flue gas sampling (target + non-target sampling, EPA OTM 45; volatile degradation products, EPA OTM 50) [163]. Currently, the state-of-the-art of pyrolysis technologies is a mass balance of organofluorine at 90% for the best preforming mineralization technologies, and lower than 60% for the poorer performing ones [110, 151].

Thoma *et al* [153] reported mostly non-detectable concentrations of target PFAS in scrubber water from full scale sewage sludge pyrolysis. The effect of flue gas cleaning on PFAS and degradation product emissions is, however, a significant knowledge gap. Likewise, the treatment of PFAS accumulated in condensate should be studied [152], with incineration and possibly catalytic oxidation being viable alternatives to simple condensate collection.

Another route to cleaner emissions could be catalytic mineralization of PFAS during the pyrolysis process. A promising and low cost option has been demonstrated by Abou-Khalil *et al* [110], heating PFOS-saturated GAC at 800 °C with Ca(OH)_2_, achieving a 98% mineralization of PFAS and resulting in CaF_2_ rather than HF formation.

#### Biochar

Biochar could be used in PFAS remediation products from diverse sectors, such as sewage-sludge biochar being used as filter material in a water treatment plant, biochar from plants grown on PFAS contaminated soil to be tilled back in the soil, etc. Biochar properties affecting sorption are highly dependent on feedstock material, pyrolysis temperature and retention time [148, 149, 154]. Biochar production should be tailored specifically to meet project sorbent requirements. High pyrolysis temperatures (≥700 °C) produce highly porous and aromatic biochars [149, 158, 160], apart from PFAS destruction during pyrolysis as described above. Sewage sludges produce good sorbents due to an abundance of mesopores (>1.5 nm) [156, 158]. Activation improves wood-based biochars by increasing mesopore PV and condensed aromatic carbon content [158, 160]. However, as biochars often fail to strongly sorb short-chain PFAS, modifications are needed. Iron doping could be a promising alternative, as it provides positively charged surfaces for interaction with anionic head groups [164].

Divalent cations can increase biochar affinity for anionic PFAS through bridging effects while monovalent cations can compress the electrical double layer, thus enhancing hydrophobic interactions [154, 157]. Low pH can also alter the net charge of biochar surfaces and hence increase sorption of anionic PFAS [154]. Dissolved organic matter can clog pores and compete with PFAS for available binding sites, weakening sorption [154, 158, 161]. Soil texture also matters, as biochar appears to work better in sandy soils compared to clay and organic soils, due to clays and organic soils being able to sorb PFAS stronger than sand [159].

While short pilot studies (<3 yrs) for biochar remediation exist [149, 154], long term filed trials (5–10 yrs) that track leaching, microbial ageing, and biochar migration are needed. Furthermore, the potential release of metals, i.e. from sludge biochars or contaminant residuals, impact the overall environmental footprint [150], and should also be studied *in situ*.

### Concluding remarks

Pyrolysis of PFAS-contaminated organic waste into biochar and its subsequent use as a PFAS sorbent could remove a significant amount of PFAS from environmental circulation. An estimate for sewage sludge treatment in Norway found that for every kg removed by pyrolysis, 10–20 kg of PFAS in soil could be have its leaching potential to groundwater reduced by soil application of sewage sludge biochars [165]. More full scale pyrolysis data and remediation field tests are needed to establish proper mass flow estimates. A focus towards this should be on integrating pyrolysis and sorbent production into current waste handling processes (figure 11), as the potential for added value/reduced costs could provide economic incentives needed for upscaling. Here, regeneration of spent biochar sorbents through pyrolysis should be explored further, as lab scale tests have shown promising results [141].

Synergies between agronomic effects/phytomanagement and sorbent stabilization of PFAS contaminated soils should also be explored. Of particular interest could be biochar stabilization of long-chain PFAS in soil in combination with phytoaccumulation of short-chain PFAS, where the contaminated biomass is pyrolyzed to produce biochar sorbents [154].

## Acknowledgements

The Norwegian Research Council (NFR; SLUDGEFFECT Project (NFR 302371); Horizon Europe Project ARAGORN (101112723).

## 11. Supercritical Water Oxidation (SCWO)

### Marc A Deshusses^1,2^, Igor V Novosselov^3^ and P Lee Ferguson^1^

^1^ Department of Civil and Environmental Engineering, Box 90287, Duke University, Durham, NC 27708, United States of America

^2^ 374Water Inc., 701 W. Main Street, Suite 410, Durham, NC 27701, United States of America

^3^ Mechanical Engineering Department, University of Washington, Seattle, WA 98195, United States of America

### Status

Destruction technologies for improved management of PFAS-contaminated wastes are urgently needed. This section presents the authors’ perspectives on the advancements necessary to mature SCWO technology for cost-effective and practical treatment applications. These developments, together with field demonstrations, will provide practitioners with an alternative for managing PFAS-contaminated liquids and solid waste streams, such as adsorbents/ion exchange resins, and could replace inadequate off-site incineration, unsustainable landfilling, and other non-destructive disposal practices.

**Figure 12.**
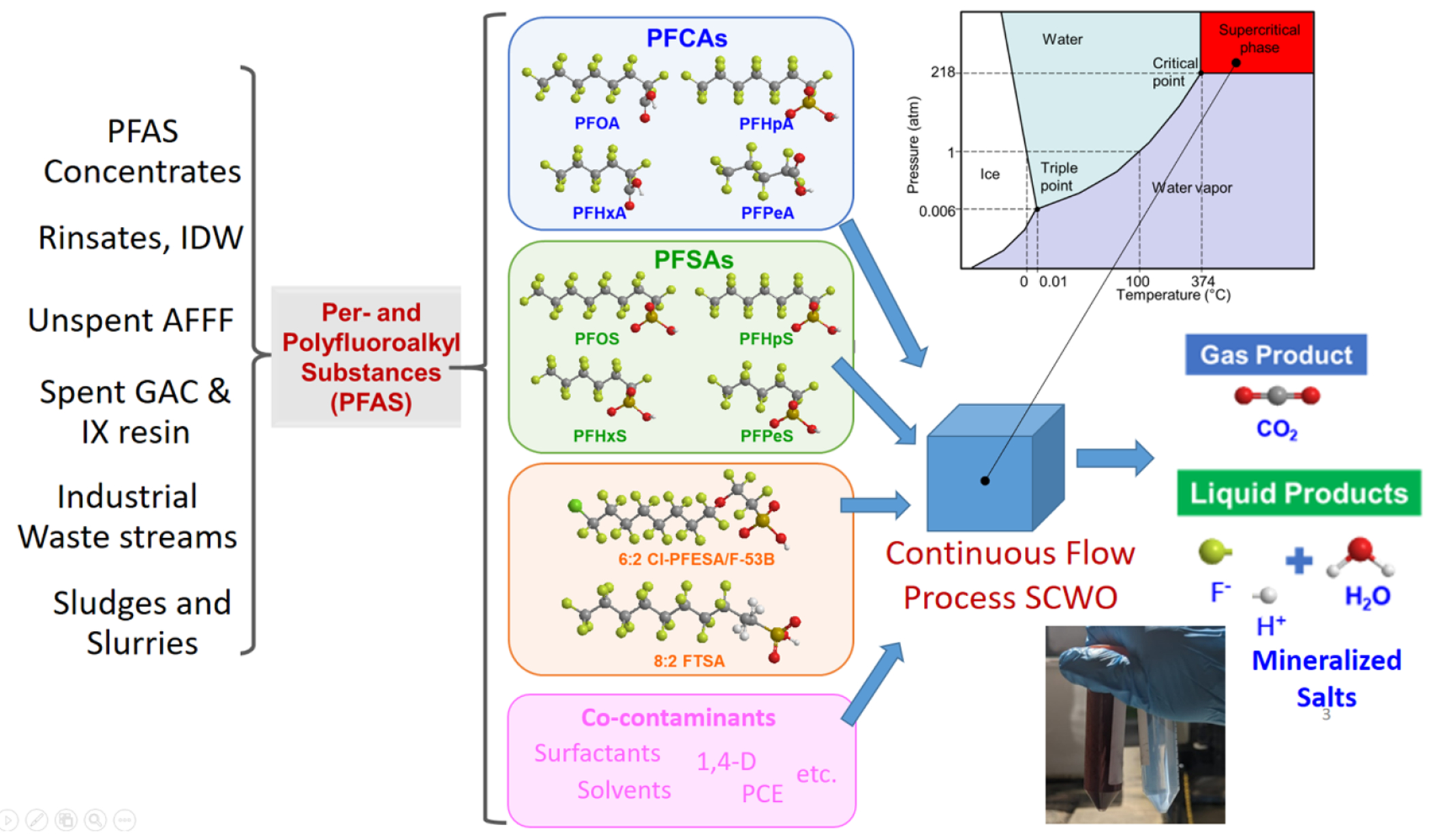
Supercritical water oxidation, a comprehensive treatment approach for mineralization of PFAS and co-contaminants to benign end-products.

SCWO is a destructive treatment technology that relies on the unique properties of water above its critical point (374 °C and 22.1 MPa). In supercritical water, in the presence of abundant oxygen, all organic contaminants (including PFAS) are rapidly oxidized and mineralized to inorganic species, including CO_2_, F^−^, and water [166]. Supercritical water is a semi-dense single phase with gas-like transport properties and solvent properties comparable to non-polar solvents (figure 12), allowing reactions to occur without mixing limitations. The chemistry of SCWO is similar to that of combustion: it is driven by free-radical reaction mechanisms involving OH•, O•, and H•. Abundant radicals, fast diffusion, and high temperatures combine to fully mineralize recalcitrant organic pollutants [167].

There are several advantages to SCWO as an end-of-life treatment for PFAS: it is very fast; it does not generate harmful byproducts such as NO_x_, SO_x_, CO, or odors; it yields fully mineralized endproducts; and it does not require catalysts or reagents other than the oxidant [168–172]. SCWO has been demonstrated in lab-scale and pilot-scale reactors for the treatment of several PFAS wastes [173, 174]. During treatment, organic fluorines are converted to fluoride ions, which may be collected as hydrofluoric acid, neutralized, or precipitated as inorganic fluoride salts with divalent cations [172]. SCWO is an exothermic process; in systems with large throughput (e.g. more than 1–2 tons/day), heat recovery can offset the substantial energy required to heat the waste above the critical point [175]. However, because of the high energy demands for heating and oxidant compression to supercritical pressures, SCWO is best suited for treating concentrated wastes or residues (e.g. foamates, AC, or ion exchange resins, other concentrates) from PFAS concentrating techniques rather than high flows of dilute waste.

### Current and future challenges

PFAS are known to resist oxidative attack by hydroxyl radicals, especially at ambient conditions, and are more susceptible to degradation via electron transfer mechanisms under non-ambient conditions [176]. Processes that facilitate electron transfer generally have low throughputs and high energy demands, making them difficult to scale. Therefore, SCWO, with its intense oxidation, is emerging as one of the most promising technologies for PFAS end-of-life treatment due to its ability to achieve rapid and complete mineralization. Organofluorine compounds are converted to HF and CO_2_ during SCWO. However, the reaction routes, elemental reaction rates, temperatures, and residence times required to achieve complete conversion are poorly understood [177]. Insights into the reaction kinetics and mechanisms, the effect of mixing rates (specific to reactor design), and the fate of fluorine, i.e. the mass balance among reactants, intermediates, and products, are needed to optimize conditions for robust remediation and treatment.

SCWO is well-suited to treat wet, hazardous, and PFAS wastes, with high destruction and removal efficiencies (DRE > 99%) and mineralization of all organic contaminants at a short residence time, ~10–100 s, and temperatures around 600 °C. Recent reports show that the SCWO process can effectively destroy neat PFAS [174, 178], simple PFAS matrices, e.g. diluted AFFF [169, 173], environmental matrices [171, 172], and PFAS immobilized on complex matrices such as spent AC and ion exchange resins [179]. There are also (non-peer-reviewed) reports of efficient destruction and mineralization of PFAS in concentrates, such as foamates from surface active foam fractionation, still bottom distillates, or biosolids. These findings underscore the potential of SCWO as a comprehensive method for PFAS destruction.

Current SCWO systems designs and scales vary considerably to meet the challenges of material strengths in high-temperature, high-pressure environments, corrosion, surface-scaling propensities, control of reaction temperature and stoichiometry, heat and energy recovery, the introduction of feedstock, and others. Several SCWO systems are under development by commercial interests, and some industrial-scale facilities have been built. The design and operation of these facilities for PFAS treatment have not been discussed in peer-reviewed literature, and there is very little guidance for SCWO operation related to PFAS treatment, especially in complex matrices or process optimization related to feedstock variability. To advance SCWO technology and reach the readiness level required for broad and robust deployment, the most urgent research needs include a better understanding of selected and interconnected aspects of treating PFAS-containing waste streams, specifically (i) temperature and reaction time requirements for specific PFAS species mineralization and desired treatment endpoints, (ii) impacts of oxidant concentration and feedstream composition on PFAS mineralization mechanisms and kinetics, (iii) understanding of conditions that lead to incomplete mineralization of PFAS and the possible formation of volatile or semi-volatile organofluorine compounds, and (iv) propensity for reactor fouling and plugging by non-soluble inorganic compounds that may precipitate in supercritical water, as a function of operating conditions and reactor design [180–182].

### Advances in science and technology to meet challenges

The temperature and reaction time requirements for complete mineralization of specific PFAS and organofluorine compounds require better definition. Some have shown that during SCWO, perfluoroalkyl sulfonates (PFSAs) react slower than PFCAs [178] and that longer-chain perfluoroalkyl homologues may react faster than short-chain ones. The few mechanistic studies that have been published suggest that PFSAs are first converted to PFCAs and 1H-perfluoroalkanes, which then undergo a successive chain scission reaction mechanism [174]. If the reaction does not proceed to completion due to low temperatures, insufficient oxidizer content, or insufficient reaction time, a release of 1H-perfluoroalkanes, which are hydrophobic and volatile, is possible. Another important question is the role of the water in SCWO-based reactions. Hydrolytic mechanisms may become significant at elevated temperatures, while the distributions of oxidative species may differ substantially from those expected at lower temperatures. Known primary oxidation/thermolysis products of PFAS, such as carbonyl fluoride, may rapidly hydrolyze to fluoride in SCWO’s ‘wet burn’ environment. A better understanding of the reaction mechanisms and, more importantly, the reaction kinetics of PFAS is essential for the effective implementation of SCWO. The complexity of this task lies in the multivariate conditions necessary for such studies, including variation in organofluorine species, temperature, pressure, oxidant availability, the concentration of co-contaminants or co-fuel, etc as well as the analytical difficulty of observing reactions directly under supercritical conditions. These studies should include the closure of the fluorine balance [174], including determining gaseous and volatile species using methods such as EPA OTM 45, 50, and 55.

There is substantial benefit in coupling experimental kinetic investigations with reaction and kinetic modeling. Reacting flow modeling in complex thermodynamic systems with phase transition presents a significant challenge, and scientific reports are limited. They typically address specific geometry or design aspects, which is appropriate in reactor design studies but may not be generalizable [183, 184]. Models have the potential to predict process design parameters for specific operational scenarios and treatment objectives, helping engineers design, operate, and control SCWO reactor systems more effectively to ensure complete PFAS mineralization.

There are also significant engineering challenges associated with scaling up the technology and achieving reliable, long-term operation in commercial settings. These include fouling of reactor surfaces and the possible plugging of the SCWO reactor by inorganic salts precipitating and depositing at supercritical conditions, as well as potential heat and mass transfer limitations that can occur when scaling up reactor systems. The former heavily depends on the waste undergoing treatment, its dissolved salt concentration, the organofluorine concentration, which will dictate pH control agent requirements, and the SCWO reactor configuration and operation. Few effective mitigation strategies have been implemented and openly disclosed. They generally rely on novel reactor designs and operation. Heat and mass transfer limitations can arise as SCWO reactors are scaled up from laboratory or small pilot-scale to industrial-scale systems. Such transfer limitations can result in cold spots or zones of subcritical fluid within the supercritical reactor. This can lead to unreacted wastes and hot spots caused by the exothermic reaction and failure to transfer heat to those cold spots. Similarly, mass transfer limitations can result in zones with limited oxidizer concentration, causing the buildup of a reactive mixture of free radical species and a local transition to much hotter hydrothermal flames, which should be avoided for safe reactor operation [185]. These challenges could be exacerbated when treating complex matrices, particle-rich feedstocks such as spent GAC or ion exchange resins, or when treating highly energetic wastes. Mitigation strategies for heat and mass transfer limitations remain limited and are still in the early stages of development, but can be broadly classified into two main categories: (1) reactor designs and means to improve feedstock and oxidant distribution and mixing in the reactor, and (2) reactor designs that are resistant to hot spots and can accommodate localized temperature or concentration gradients. Scaling up by numbering up (i.e. duplication) proven pilot reactors, instead of engineering new large reactors, may also be possible. Ultimately, it is unlikely that there will be a single, universal solution, and overcoming these challenges will probably require a combination of design innovations, advanced process monitoring and controls, and operating strategies.

### Concluding remarks

SCWO is a promising approach for effectively destroying PFAS, offering the potential to completely mineralize all PFAS and organofluorine compounds, as well as co-contaminants, into benign end-products. While the SCWO technology has demonstrated significant efficacy, further research and demonstrations are essential to optimize reaction conditions and reactor design, as well as to address the engineering challenges associated with reactor scale-up and process robustness. Additionally, techno-economic and life cycle assessments will inform the selection of technology for large-scale PFAS treatment. With continued advancements in these areas, SCWO has the potential to become a robust and sustainable solution for PFAS remediation, supporting both environmental and public health objectives.

## Acknowledgements

The funding for the University of Washington SCWO system was provided by the Defense Threat Reduction Agency (DTRA) - Grant HDTRA1-17-1-0001 and a Cooperative Research and Development Agreement (CRADA) with the Army Research Office (ARO) - Project Number CB10397.

## 12. Applications of Hydrothermal Alkaline Treatment (HALT) for PFAS-contaminated waste

### Igor V Novosselov^1^, Brian R Pinkard^1,2^ and Timothy J Strathmann^3^

^1^ Mechanical Engineering Department, University of Washington, Seattle, WA 98195, United States of America

^2^ Aquagga, Inc., 748 Market St, Tacoma, WA 98402, United States of America

^3^ Civil and Environmental Engineering Department, Colorado School of Mines, Golden, CO 80401, United States of America

### Status

**Figure 13.**
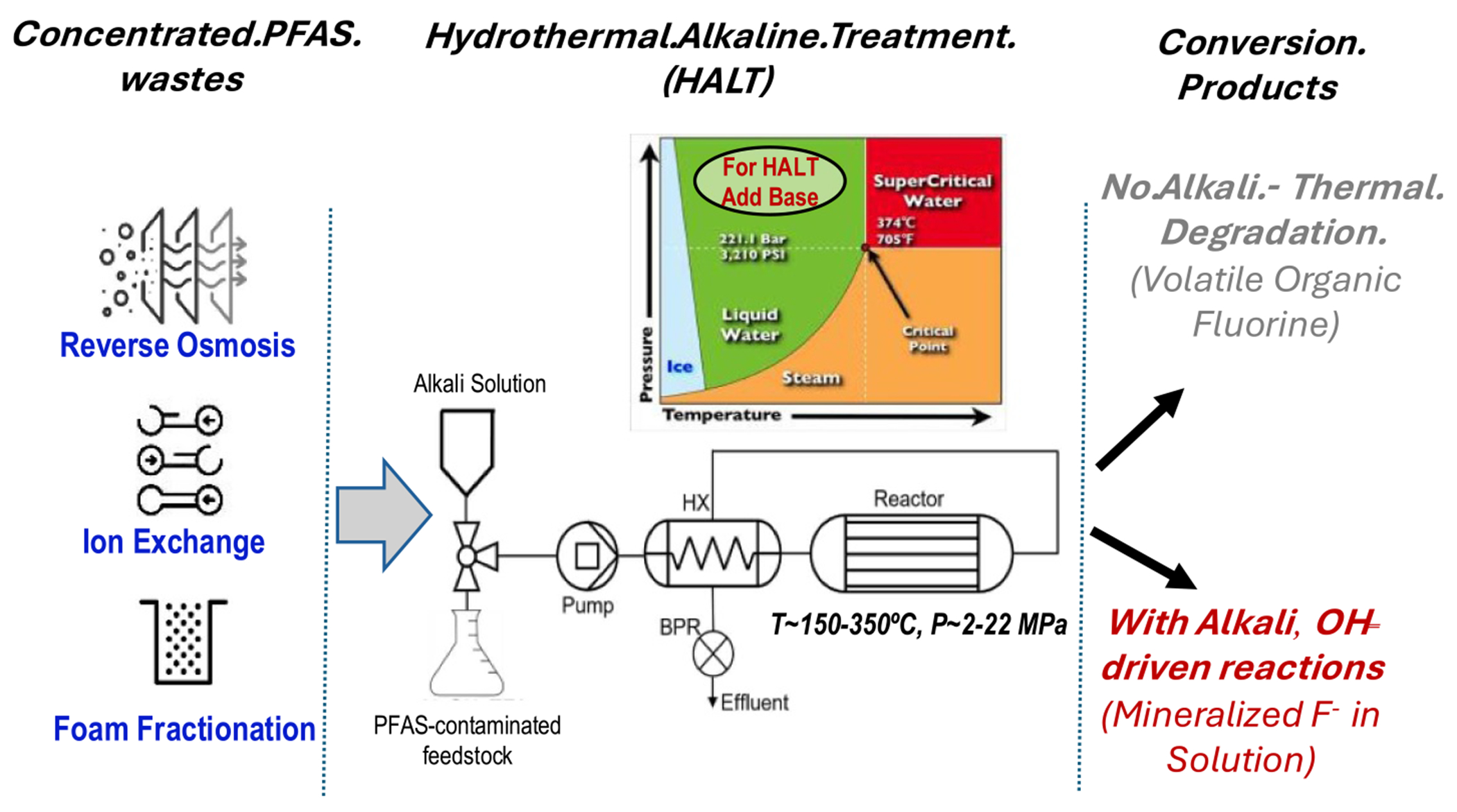
Applications of HALT processes for treatment of PFAS-contaminated waste.

Hydrothermal methods have been used to destroy PFAS in subcritical water under alkaline conditions and supercritical water under oxidative conditions. HALT has shown significant promise as a technology for the destruction of PFAS, and recent work has studied the underlying chemical processes and demonstrated application for a variety of PFAS-contaminated matrices at a variety of scales, ranging from small laboratory batch reactors to field-scale reactors capable of treating gallons per hour of throughput. This work summarizes the background and current state-of-the-art and shares the authors’ opinion on the maturation and practical application of HALT processes. Figure 13 illustrates the uses of HALT in the treatment of PFAS-contaminated wastes.

By its name, HALT affects PFAS destruction by combining subcritical hydrothermal conditions (liquid phase water, typically *T* ~ 200 °C–374 °C, *P* ~ 2–22.1 MPa), with strong alkali amendments that release elevated concentrations of the OH^−^ in the reaction medium. This combination was initially identified from an amendment-screening experiment conducted at the Colorado School of Mines (CSM), where fluoride ion release was measured after exposing PFOS to a series of hydrothermal solutions amended with potential reactive species, including strong oxidants, reductants, acids, bases, and metal nanoparticles [186]. The highest concentrations of F^−^ were observed for solutions amended with NaOH (strong base), sodium borohydride (strong reductant), and potassium permanganate (strong oxidant). However, measurements also showed that all three reagents acted to raise the solution pH, and subsequent tests showed that this was the causative reason for PFAS degradation and defluorination in the reaction solution.

Laboratory scale batch experiments have shown that the full suite of PFAS detected in AFFFs through targeted LC-MS/MS and LC-HRMS suspect screening analysis are degraded and defluorinated by HALT [187]. The extent of destruction for all PFAS is highly temperature dependent, but results show that some subclasses of PFAS degrade in the absence of alkali amendments (e.g. PFCAs) [188], whereas other subclasses require strong alkali in addition to near-critical reaction temperatures (e.g. PFSAs) [186, 187]. This is attributed to different mechanisms that initiate the destruction of the individual PFAS subclasses. Degradation of PFCAs is initiated by thermally driven decarboxylation reactions [188], whereas PFSA degradation, in the temperature range of HALT reactors, is proposed to be initiated via attack by the strong nucleophile OH– [186, 189].

### Current and future challenges

A mechanistic understanding of the HALT process for PFAS destruction needs further evaluation to optimize the process and reduce the consumption of chemicals and energy. While the studies of neat compounds are relatively straightforward, one of the major challenges is to address the effect of co-contaminations and apply the process to more operating scenarios. Recent laboratory studies with batch reactors conducted at the CSM have extended the application of HALT for the destruction of PFAS in a variety of contaminated matrices, including groundwater and soils [190] and foam fractionation-derived liquid concentrate [191]. Apparent rates for the transformation of individual PFAS have been found to be largely insensitive to the media [191], but there is a need to account for any reactions with the media that consume OH^−^ (e.g. OH– reactions with silica-containing soil minerals) [190]. Notably, while alkali is not required to degrade PFCAs, it is still necessary to convert the organically bound fluorine to inorganic F^−^. Austin and coworkers [188] showed that trifluoroacetate, a C1 PFCA, degrades at similar rates in the absence and presence of NaOH, but mineralization to F^−^ and CO_3_^2−^ only occurs when NaOH is added; fluoroform (CHF_3_) forms as the terminal product when no NaOH is added to the reaction solution. The thermal degradation step may be similar to SCWO systems; however, the OH^−^ route significantly differs from SCWO chemical kinetic routes.

Potential applications of HALT can also be used to destroy other fluorinated compounds. For example, hydrofluorocarbon (HFC) refrigerants are potent and long-lived GHG [192]. HFC refrigerants are also known to decompose into fluorocarbons such as trifluoroacetic acid (TFA) in the atmosphere [193–195], which now exist in concerning concentrations in rainwater. While existing thermal, chemical, and plasma destruction processes can achieve >99.99% destruction of HFCs, there is a need for more cost-effective HFC destruction technologies that are proven to mineralize legacy HFCs completely. By themselves, HFCs are resistant to thermal degradation, e.g. fluoroform CF_3_H is stable up to *T* ~ 900 °C [196] or *T* ~ 600 °C in the presence of water vapor [197]. However, in the presence of alkali (e.g. NaOH), alkaline hydrolysis can occur at *T* < 150 °C [188]. HALT can present an interesting opportunity for carbon capture application [198].

### Advances in science and technology to meet challenges

Continuous flow hydrothermal systems are advantageous in practical applications and enable real-time process monitoring and control. Continuous flow HALT (CF-HALT) was first tested at the University of Washington on a bench scale for single compound PFAS solutions [188] and PFAS-impacted fire training pit water [199]. Optimizing the process economics of a continuous HALT reactor is necessary for industrial applications, and heat recovery is critical to process economics, most commonly accomplished by a recuperative heat exchanger. Lab-scale and commercial-scale CF-HALT systems utilizing a recuperative heat exchanger have been recently demonstrated [188, 200]. High pH concentration during treatment is necessary for treating PFAS-contaminated waste, specifically for PFSAs like PFOS; previous reports indicate the need to add >1 M NaOH to achieve significant destruction rates. At high temperatures, the exposure may lead to reactor vessel and heat exchanger corrosion; thus, material selection and corrosion control are critical to the long-term operation of the systems [201]. These challenges are specific to treatment conditions such as required temperature and the levels of alkali. Material selection and reactor vessel design can extend the operation of the reactor. Another practical challenge is the potential precipitation of fluoride species such as CaF_2_ and MgF_2_ when treating feedstocks with hard minerals present. Mineral precipitation may require periodic cleaning and maintenance of commercial-scale systems. These and other long-term operational challenges are the focus of ongoing research and development efforts.

**Figure 14.**
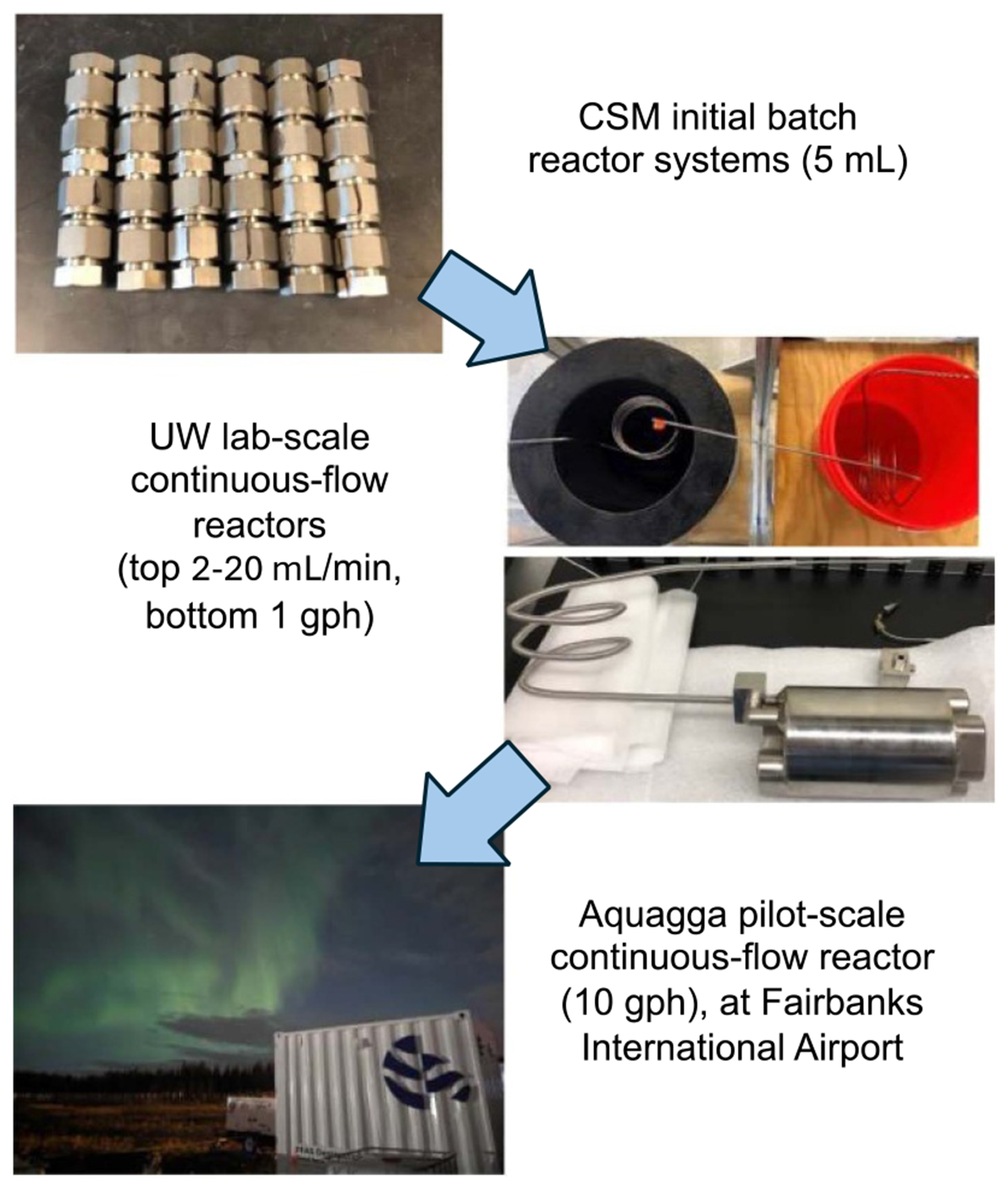
HALT studies of PFAS treatment and destruction have progressed from lab-scale batch reactors (left) to lab-scale continuous-flow reactors (middle) to demonstration-scale pilot systems (right).

Process scale-up, selection of ancillary components, and fabrication of robust pressure vessels are not typically a good fit for academic research. These challenges need to be addressed during technology commercialization. Figure 14 shows the research reactors used for the development of the technology. Recently, several field demonstrations of pilot-scale reactors were performed by Aquagga, Inc. (table 3). These have focused on treating PFAS-rich liquids, including industrial wastewater, foam fractionates, AFFF, and sorbent regeneration brines. For all field demonstrations, a containerized HALT system was mobilized to a site and operated for up to several weeks. The systems were typically operated at a throughput between 5 and 10 gph. In the last 5 years (since 2019), the HALT process has progressed from small-scale batch reactors (CSM) to bench-scale continuous flow reactors (UW) to successful field demonstration of pilot systems (Aquagga); this rapid technology maturation attests to the potential of the HALT for treating PFAS-containing waste streams. Significant effort is needed to demonstrate the technology at scale and in complex treatment scenarios. Long-term operation of the systems will allow for further optimization of the systems and provide data on the applicability of the HALT for the treatment of industrial and environmental PFAS-contaminated waste streams.

### Concluding remarks

HALT shows the most unique value in treating PFASs in liquid or brine matrices. HALT can destroy ultra-short-chain PFASs and can process feedstock with high total dissolved solids, which enables PFAS destruction in industrial wastewaters (*e.g.* chemical producers, semiconductor fabs, etc). HALT can handle very high starting PFAS concentrations (>1000 mg L^−1^), which can be advantageous when coupled with appropriate PFAS separation and concentration technologies, such as regenerable sorbents or foam fractionation [191, 202, 203]. Current efforts explore HALT for on-site regeneration of sorbent media [121] and mineralizing PFAS in biosolids while simultaneously recovering valorizable products (e.g. biofuel, fertilizers) [204]. These applications will likely require modified reactor systems to introduce and process wet solid-containing feeds.

While the disposal of fluorinated compounds via HALT is still in the pilot demonstration scale, technology maturation and industrial-scale deployment require additional fundamental studies, applied research, and demonstration of HALT reactors at scale in relevant environments. The CF-HALT offers several tangible benefits, namely:

- Rapid mineralization of organic fluorine, facilitating the high throughput systems
- No release of volatile organic fluorine compounds of dissolved organic fluorine
- The electrically heated thermochemical process with integrated heat recovery requires very low energy consumption for the destruction reaction.

## Acknowledgements

SERDP (ER18-1501) and ESTCP (ER21-7591 and ER23-8400) funded this work. The US Army Corps of Engineers provided financial support for TJS through cooperative Agreement W912HZ-23-2-0009. The fabrication and demonstration of Aquagga’s scaled-up HALT systems has been supported by EPA SBIR Phase II contract 68HERC22C0038, NSF SBIR Phase II Grant 2232969, DARPA SBIR Phase II Contract W912CF-23-C-0006-P00001, and contracts with the Alaska DOT&PF, the 3M Company, Brice Environmental, and the US Army Corps of Engineers Engineering Research and Development Center.

## 13. Ball milling

### Kapish Gobindlal^1,2^ and Jonathan Sperry^2^

^1^ Environmental Decontamination (NZ) Limited. 1/22 Highgate Parkway, Auckland 0932, New Zealand

^2^ Centre for Green Chemical Science, University of Auckland. 23 Symonds Street, Auckland 1010, New Zealand

### Status

Ball milling, also known as mechanochemical destruction (MCD), is a solid-state mechanical process that has gained significant attention in recent years as a technique for the destructive treatment of hazardous organic substances. As a subset of mechanochemistry (mechanically induced chemical reactions), ball milling utilizes the mechanical energy from intense ball-to-ball and ball-to-surface collisions to initiate and promote the mineralization of organic pollutants in contaminated matrices or outdated pure products [205].

For the remediation of contaminated media and the treatment of chemical stockpiles, the benefits of MCD include the absence of a need for solvents, lower energy requirements compared to conventional destruction processes, and the fact that mechanochemical reactions typically occur under ambient conditions (e.g. temperature, pressure, humidity) [206–208]. The inherent efficiency of these reactions has led to mechanochemistry being recognized as aligning with the principles of Green Chemistry, which focuses on minimizing the environmental impact of industrial processes by promoting sustainable scientific concepts and implementing innovative technological solutions [209]. This is particularly significant, as conventional treatment technologies often struggle to effectively destroy PFAS due to the persistent nature of these compounds. Furthermore, several published studies on the use of ball milling for PFAS-impacted matrices were cited in a recent review on mechanochemistry and the UN Sustainable Development Goals [210]. This review emphasized the alignment of mechanochemistry with global policy frameworks and its potential to address real-world waste management challenges, including PFAS pollution and legacy chemical stockpiles.

**Figure 15.**
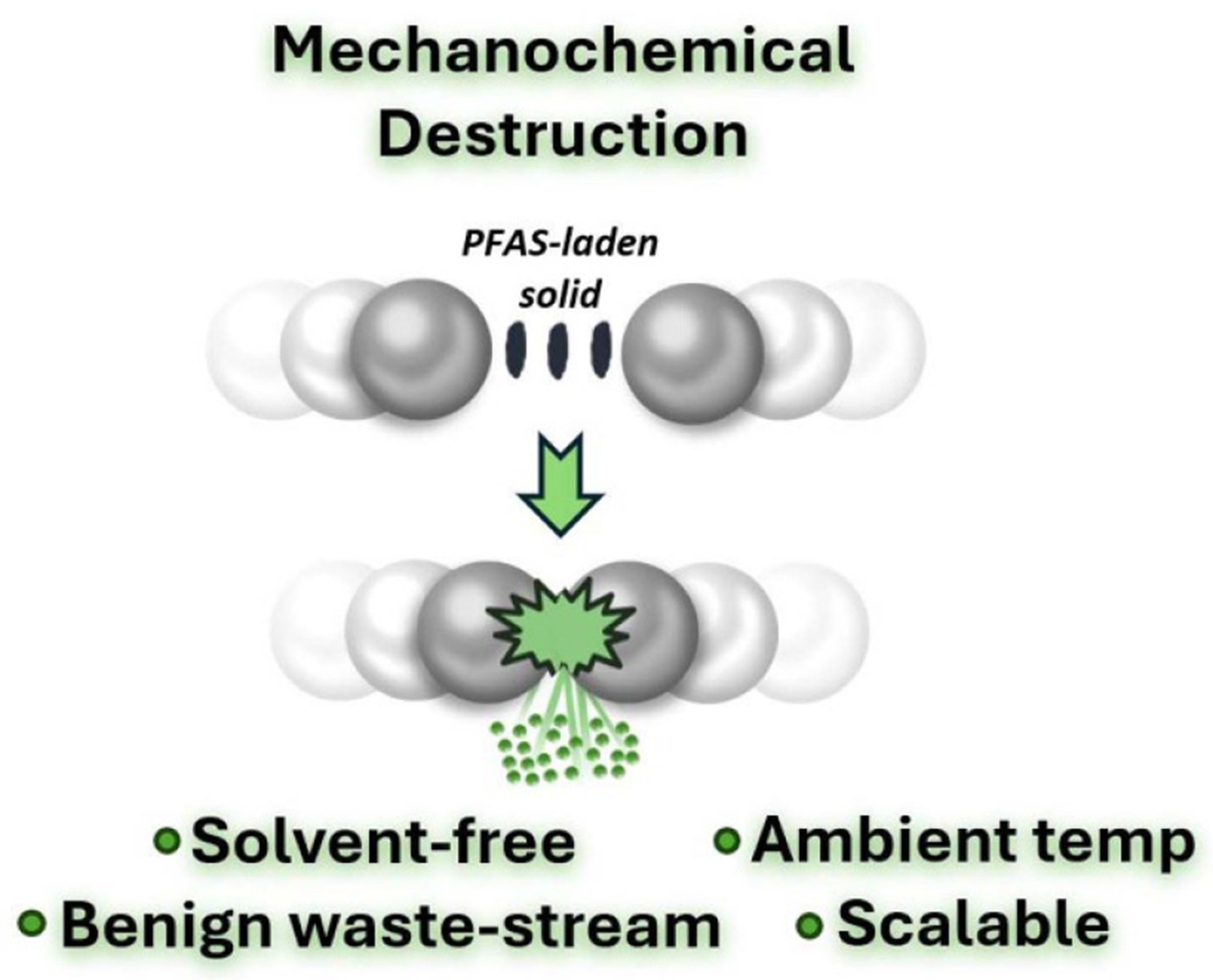
Ball milling collision profile, leading to particle fracture and PFAS destruction.

In the mid-1990s, Rowlands and colleagues were the first to demonstrate the degradation of the obsolete pesticide dichlorodiphenyltrichloroethane under mechanochemical conditions [211]. Since then, this specialized function of mechanochemistry has grown exponentially, with several research groups worldwide conducting MCD studies primarily targeting compounds listed in the Stockholm Convention on POPs. More recently, ball milling has demonstrated compelling results for the destruction of PFAS. In 2013, Zhang and colleagues showed that PFAS can be effectively destroyed through ball milling, revealing that 100% of PFOA was reduced to below the limit of detection after a milling time of 3 h and 99.88% of PFOS after milling for 6 h [212]. These promising initial results and the validation of PFAS destruction efficacy have led to a significant increase in the use of ball milling for the destruction of PFAS in obsolete products and PFAS-laden solid media, as highlighted in figure 15 [213]. Also, MCD of PFAS has recently been coupled with fluorine recovery by utilizing potassium phosphate salts as co-milling agents to produce KF and K_2_PO_3_F, thereby demonstrating a potential circular economic pathway for transforming PFAS into high value fluorochemicals [214]. Similarly, fluoropolymers (e.g. polyvinylidene fluoride, polytetrafluoroethylene) have been mechanochemically milled in the presence of potassium bases to generate KF black that acts as a fluorinating agent [215].

The advancement of ball milling as a destruction technology will ultimately provide the waste management sector with a novel tool to tackle real-world challenges related to PFAS-contaminated media (e.g. soil) and PFAS-containing obsolete products (e.g. AFFF concentrates). However, despite the promising results achieved so far, several technical and commercial hurdles must be addressed before ball milling systems can be effectively deployed for PFAS destruction at an industrial scale.

### Current and future challenges

Despite the effectiveness of ball milling in destroying PFAS and the overall advantageous properties of the technique, there are still unresolved questions regarding the fundamental chemistry of the process. Additionally, the commercial feasibility of designing, engineering, building, and validating deployable MCD systems has received little attention to date. Addressing these aspects is essential for advancing ball milling from benchtop laboratory-scale trials to technology implementation for PFAS destruction at larger scales. Although it may seem premature, researchers should consider both fundamental technology aspects and implementation challenges simultaneously; otherwise, research avenues may be pursued that cannot effectively address real-world challenges.

Recent studies have shown high destruction rates by ball milling for a wide variety of PFAS subgroups (e.g. perfluorosulfonic acids, perfluorocarboxylic acids, fluorotelomer sulfonates), with destruction efficiencies ranging from 99% to 100% achieved in laboratory-spiked solid samples, authentic contaminated soils, and AFFF concentrates [212, 213, 216–219]. While demonstrating process efficacy is important, very few studies have adequately evaluated the underlying reaction mechanisms, assessed the degradation products present in the solid matrix and gaseous phase, explored reaction kinetics, or determined the fate of PFAS constituents—particularly fluorine—following the completion of the destruction process. The question of whether ball milling can destroy PFAS has been sufficiently addressed; the more pressing inquiries now relate to *how* and *why* these reactions occur, as well as the importance of the fluorine mass balance [130].

Focusing on the uncertainties related to the underlying science of PFAS destruction by ball milling will directly inform technology implementation and optimization. However, scaling up any treatment process is a complex task, especially for an emerging technology like MCD. Currently, almost all research on PFAS destruction by ball milling has utilized benchtop devices and various co-milling agents to promote or enhance PFAS destruction. The selection of suitable reagents, when necessary, requires careful consideration of secondary waste management. For instance, while achieving nearly complete conversion of C–F bonds to inorganic fluoride using potassium hydroxide as a co-milling reagent has been well demonstrated, this approach results in an undesirable end product with a high pH and generates toxic potassium fluoride. Additionally, the commercial availability of suitably designed and engineered pilot-scale and large-scale ball milling systems is a significant barrier that hinders the verification of scalability and the field testing required to validate the MCD process for PFAS destruction.

### Advances in science and technology to meet challenges

Addressing the issues and challenges outlined above requires a holistic approach that considers the structural transformations of solids subjected to mechanochemical conditions, the changes in reactivity of mechanically activated substances, and the subsequent degradation of PFAS in these reactive environments. Researchers should employ a comprehensive suite of conventional and novel analytical techniques to gain an in-depth understanding of mechanochemically induced destruction processes for PFAS treatment, ensuring that reaction mechanisms are well characterized and treatment goals are met by the end of the MCD process. Utilizing this overall strategy will accelerate and streamline the validation of progressive technology, moving from benchtop-scale research to development at pilot and demonstration scales, ultimately leading to full scale technology deployment.

A thorough examination of solid and gaseous samples collected before, during, and after the milling process is necessary to elucidate destruction efficiencies, intermediate degradation products, matrix effects, and reaction kinetics. The analytical techniques listed in table 4 provide examples of methods for evaluating PFAS destruction and structural changes to the matrix. It is imperative that both aspects are investigated concurrently due to the dynamic relationship between the structural transformations of PFAS-laden solids during mechanochemical milling and the subsequent interactions at solid interfaces that lead to PFAS destruction. Advances in *in situ* real-time monitoring of PFAS concentrations (in both solid and gaseous phases) and structural changes would significantly enhance our understanding of the underlying destruction mechanisms, directly informing optimization, scaling up, and operational procedures.

The development of operational large-scale ball milling systems is essential to validate the scalability of the MCD technique for treating PFAS-laden solids (e.g. soils and sediments) and PFAS-containing products (e.g. AFFF concentrates) [220]. Significant innovations in the design and fabrication of MCD systems are needed to facilitate pilot-scale trials, field demonstrations, and ultimately the full scale implementation of continuous processing systems. Systematic and targeted validation of these systems is essential for advancing ball milling from a primarily laboratory-based PFAS destruction technique to an effective and deployable technology. Scale-up trials utilizing a 267 l horizontal tumbling ball mill to treat 50 kg batches of PFAS-impacted soil provided promising initial results for target PFAS [221]. Additionally, active MCD demonstration projects targeting PFAS-laden environmental media (e.g. soil, sediment) were underway at the time this Roadmap was prepared [222, 223].

In addition to the necessary R&D-focused advancements, it is crucial to consider other influencing factors that significantly impact the progressive development of ball milling for PFAS destruction. These factors include PFAS regulations, capital requirements for developing emerging technologies, intellectual property considerations, market demands, and the highly specialized expertise needed to execute a targeted strategy for complex waste treatment applications.

### Concluding remarks

The inherent stability and environmental persistence of PFAS make effective destruction a complex challenge for any technology, whether novel or conventional. The continued development of ball milling as a destruction technique for PFAS requires a multifaceted approach that encompasses fundamental and applied research, scale-up efforts, and demonstration projects.

For MCD to be recognized as an effective destruction method for PFAS-laden solids and PFAS-containing products, robust lines of evidence must be established, particularly focusing on identifying mechanistic degradation pathways, the kinetics of reaction progression, and the fate of mineralized substances. Additionally, benchtop and scale-up case studies addressing challenges faced by the waste management and environmental sectors are needed to demonstrate the viability of ball milling as a treatment technology. As the knowledge base expands and data are published, MCD will advance toward technology implementation for addressing complex PFAS issues.

## 14. Electrochemical Oxidation (EO)

### Elisabeth Cuervo Lumbaque^1^, Nick Duinslaeger^1^ and Jelena Radjenovic^1,2^

^1^ Catalan Institute for Water Research (ICRA), Emili Grahit 101, 17003 Girona, Spain

^2^ Catalan Institution for Research and Advanced Studies (ICREA), Passeig Lluís Companys 23, 08010 Barcelona, Spain

### Status

**Figure 16.**
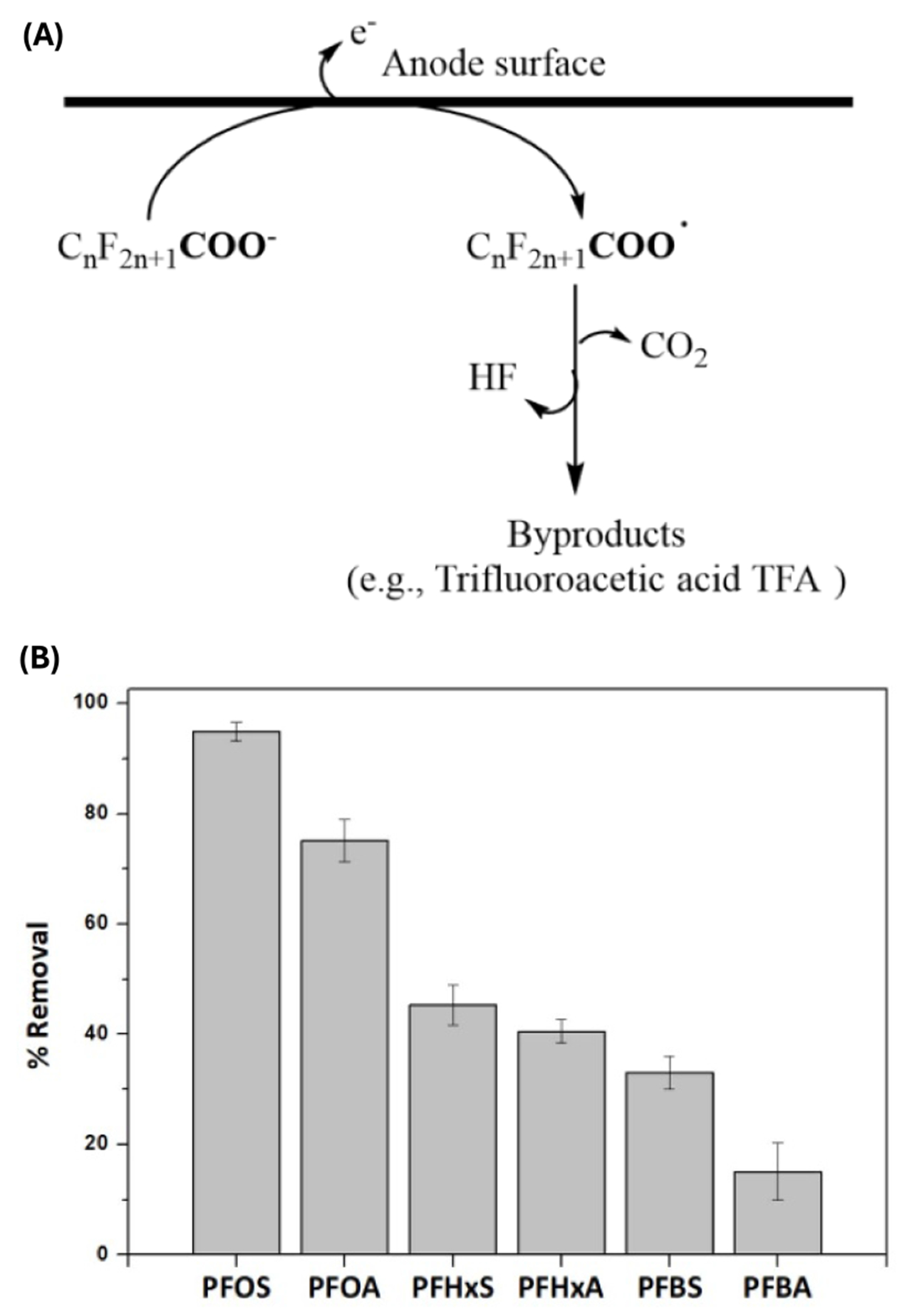
Schematic overview of (A) potential-driven electron transfer reaction for the oxidation of PFAS and (B) removal of PFAS (C4–C8) at 575 A m^−2^ anodic current density using leachate in one-pass mode.

Electrochemistry, the very technology behind the industrial production of PFAS, has the potential to solve the issue of ‘forever chemicals’ in our water cycle [224]. EO is an advanced oxidation process that uses catalytic electrodes and an electric current or potential difference between anode and cathode to drive a variety of reactions, generating hydroxyl radicals (HO^•^) and/or other oxidizing species depending on the anode material and the type of supporting electrolyte used. In the case of PFAS, oxidation begins with direct electron transfer from the PFAS molecule to the anode at high anodic potentials (typically >3 V vs. Standard Hydrogen Electrode, SHE). This is followed by the cleavage of functional groups and subsequent radical reactions with oxidant species, leading to the formation of shorter-chain perfluoroalkyl compounds (⩽C6), CO_2_ and HF through a CF_2_-unzipping cycle (figure 16(a)). Key advantages of EO include reagent-free operation, no residual waste, ambient conditions processing, robustness, and energy-efficiency [225]. The energy consumption for EO ranges from 4–132 kWh m^−3^ [226] in contrast with 310–1600 kWh m^−3^ SCWO [168] and 1–300 kWh m^−3^ (plasma) [227].

Several studies have demonstrated the exceptional performance of mixed metal oxide (MMO) (e.g. SnO_2_ and PbO_2_ coatings), boron-doped diamond (BDD), and Magnéli phase Ti_4_O_7_ anodes in PFAS removal (>90% for long-chain PFAS) [228]. However, electrode material development is being redirected to prioritize non-toxic, earth-abundant elements, and PFAS-selective materials that avoid the use of precious metals and have a scalable and low-cost manufacturing method.

In this regard, materials based on carbon nanostructures (e.g. MXene-based, carbon nanotubes, and graphene-based electrodes) are being explored as alternatives. Their high surface area, electrical conductivity, and tuneable surface chemistry enable efficient removal of PFAS. Among these, reduced graphene oxide (RGO) electrodes stand out for their cost (less than €50/m^2^ vs. ~€5000/m^2^ for BDD [229]), moderate energy consumption (~10 kWh m^3^ compared to 5–49 kWh m^3^ using Ti_4_O_7_ anode [226, 230]), antifouling properties and, have shown promising PFAS removal in real effluents [229] (figure 16(b)). Importantly, they do not form toxic chlorinated byproducts, a major limitation of EO in (waste)water treatment [231]. Meanwhile, MXenes, with their rich surface chemistry and layered structure, offer excellent electrosorption capabilities and have been studied for integration with conducting polymers [232].

### Current and future challenges

Electrochemical systems are very well-suited for decentralized and distributed water treatment, for example as point-of-entry (POE) and point-of-use (POU) devices and can also be designed to treat varying types of industrial, PFAS-contaminated water [233]. Historically, EO systems struggle to be applied at a wider-scale for (waste)water treatment due to major limitations: elevated energy demand due to limited surface area of high-cost commercial electrodes; mass transfer limits at trace levels; formation of chlorinated organic byproducts; and in the case of PFAS: inefficient removal of short-chain homologues and incomplete fluorine mass balance.

**Figure 17.**
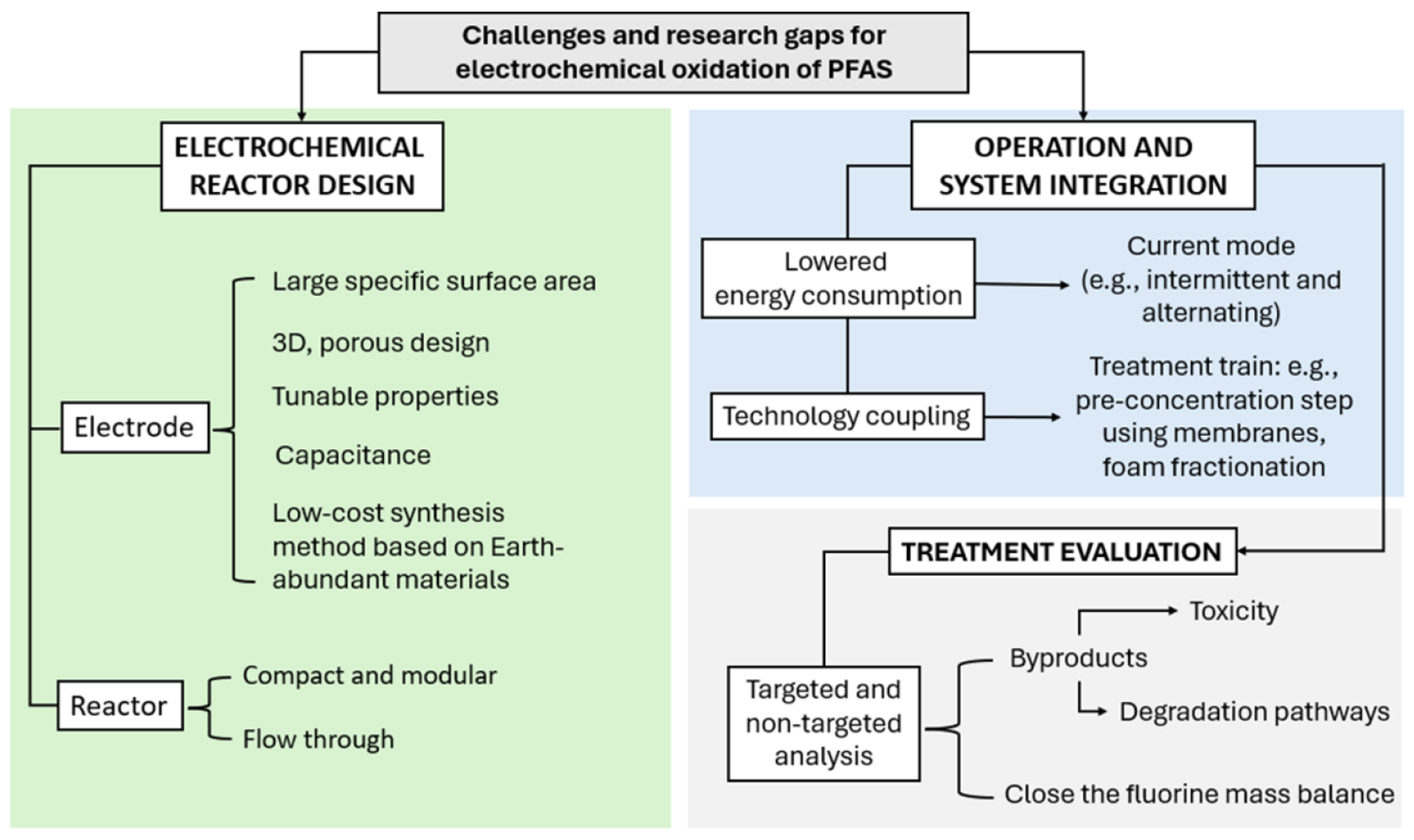
Challenges and research gaps to achieve optimal electrochemical water treatment for PFAS removal.

Addressing these challenges requires innovation in electrode design, particularly the development of materials that are both cost-effective and high performing for widespread adoption (figure 17). In parallel, green and scalable synthesis approaches also merit further investigation to minimize secondary pollution. To exploit the full potential of electrode materials, careful reactor engineering is necessary to avoid the accumulation of gas bubbles (O_2_, H_2_), which can hinder electrode contact and reduce long-term performance. Switching from two-dimensional (2D) to three-dimensional (3D) electrochemical cells in flow-through mode can significantly increase the reaction rates. To improve efficiency and meet industry demands, further optimization of hydrodynamic conditions, electrode geometry and operational modes is needed.

Regarding the operation system, high-salinity waters demand the use of materials electrochemically inert to chloride, thereby preventing side reactions and minimizing the formation of toxic byproducts. Additionally, strategies such as cathode modification for increased generation of H_2_O_2_, or it is controlled dosing can be employed to mitigate the impact of side reaction. Energy consumption must be further reduced (e.g. via current modulation, intermittent operation leveraging the electrode’s capacitance to improve cost-competitiveness or alternative current mode to mitigate fouling and aging).

In terms of fluorine mass balance, monitoring PFAS degradation via fluoride release can become challenging in real matrices due to low PFAS levels and complex chemistry [130]. Therefore, both targeted and nontargeted analyses are essential to understand degradation pathways. It is also crucial to assess the impact of HF as a corrosive byproduct that reacts with the anode surface.

Regulations in Europe are becoming more rigorous, yet no current water treatment system can efficiently handle a broad range of PFAS. In the conservative field of (waste)water treatment, new technologies typically take decades to mature and reach real-life application. Recently, the need for decentralized water management, public concern about PFAS contamination, and growing awareness of toxic chemicals are accelerating this process. Therefore, it is important that breakthrough advances in EO find ways to break the barrier to the market so they can provide solutions to the pressing and pervasive environmental challenges of our time.

### Advances in science and technology to meet challenges

Advances in nanotechnology have provided greater control over electrode material properties, and optimizing the electrode surface area allows more reactant molecules to meet the electrode reducing mass transfer limitations, thereby directly improving the oxidation efficiency [234]. For instance, the use of 2D nanomaterials offers promising opportunities due to their unique electronic properties, high surface areas, and tunable surface chemistries [235]. Selective coordination of PFAS functional groups can enable a ‘concentrate-and-destroy’ approach, i.e. localization of PFAS molecules on a surface by using adsorption/electrosorption, and destruction through targeted delivery of reactive agents at that surface (e.g. UV light and/or electrical current). By going down to the nanoscale the reactivity per atom increases, and nanomaterials employed for contaminant removal from water display accelerated reaction kinetics, self-regeneration properties and high selectivity. For example, doping of the graphene coating with 2D borophene instead of atomic boron can tailor the activity towards the production of oxidant species e.g. enhancing the amount of HO· radicals electrogenerated at the electrode by an order of magnitude [236].

At this point, the use of nanomaterials does not significantly increase the cost of electrodes, as they are typically added in trace amounts that do not greatly impact production costs. In addition to the exploration of new base materials, more innovative strategies for electrode preparation are being researched to improve catalytic performance, scalability, and lower production cost. These strategies include the use of lasers, microwave heating, pulsed electrodeposition and 3D printing. The growing accessibility of 3D printing has improved electrode architecture and reactor design by precisely controlling electrode geometry [237]. Numerous 3D electrode materials are available, including carbon-based structures, metal composites, and conductive polymer composites, all of which can be enhanced with tailored catalytic coatings using deposition methods [238]. This has also led to the development of electroactive membranes, which significantly reduce electrofiltration energy consumption by delivering electrical energy directly to the membrane-water interface, where the separation occurs [239]. To support and accelerate such advancements, mathematical models based on experimental parameters are developed to optimize electrode geometries and reactor designs, reducing trials and operational costs [240].

### Concluding remarks

Currently, the environmental challenges are insurmountable to the implemented treatment technologies and innovative solutions are needed to help address them. By improving electrode configuration, reactor geometry, and utilizing simple and scalable synthesis methods, an unprecedented performance in removing ‘forever chemicals’ from water can be achieved. Interdisciplinary research at the interface of electrochemistry, nanotechnology, environmental science, and analytical chemistry is improving the interaction and selectivity of PFAS at the electrode surface. These advances are empowering the next generation of water treatment systems, capable of removing PFAS even from the most complex waste streams. The complete destruction of PFAS is crucial due to their widespread presence in water and associated health risks, making PFAS contamination a major social issue demanding effective solutions from scientists and policymakers. The industry, traditionally conservative, is now being induced to adopt innovative approaches and EO systems will play a key role in this transformation, offering unprecedented performance in removing persistent chemicals from water.

## Acknowledgements

The project FOCUS4PFAS has received funding from the European Union’s Horizon Europe research and innovation programme under Grant Agreement No 101062078.

## 15. *In Situ* remediation

### James Hatton^1^ and Karl Bowles^2^

^1^ Jacobs, Greenwood Village, CO 80111, United States of America

^2^ Jacobs Group, North Sydney, 2060, Australia

### Status

**Figure 18.**
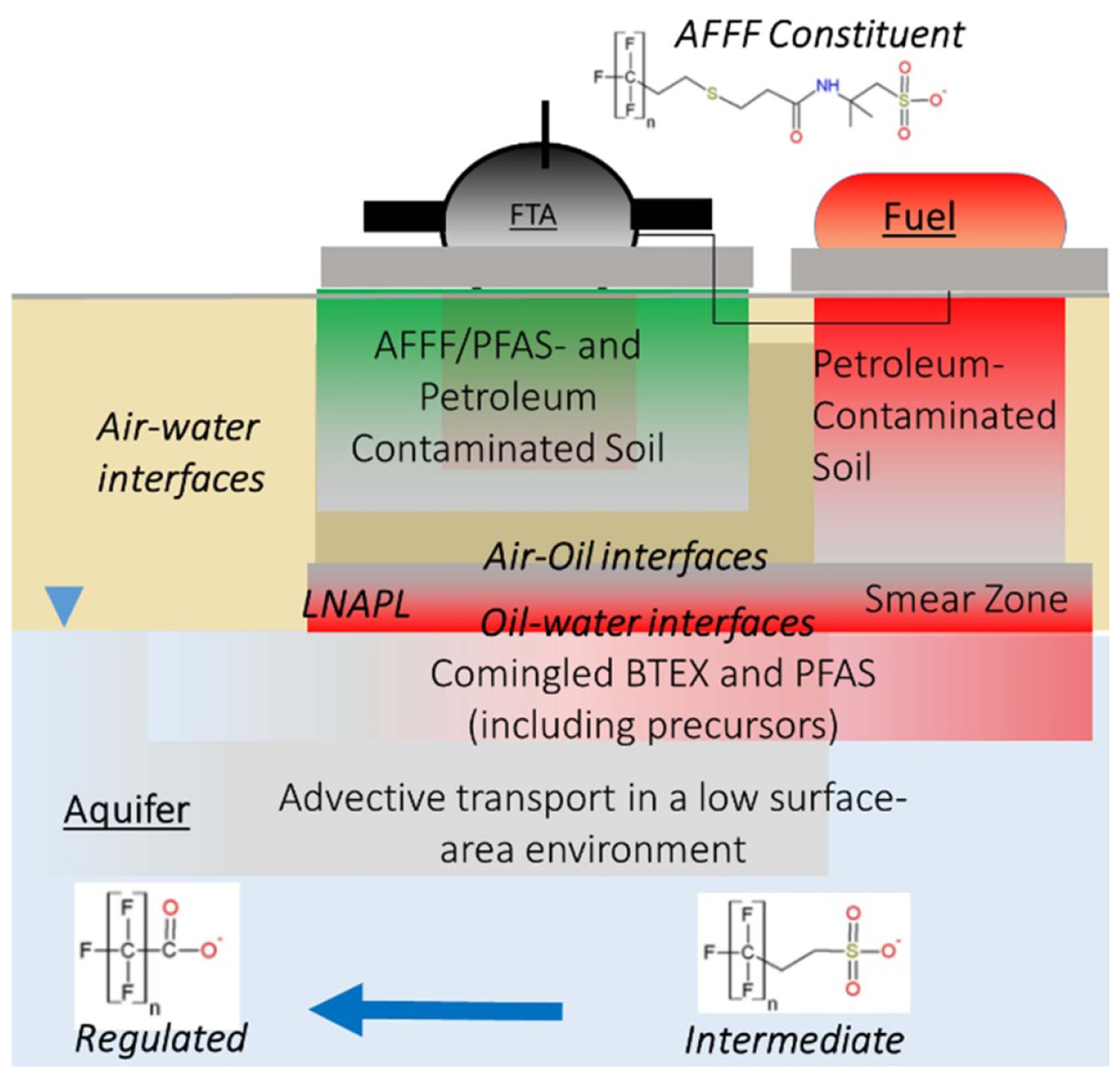
Conceptual representation capturing some of the complexities of PFAS occurrence in the sub-surface at an AFFF impacted fire training area.

Remediation of contaminated sites is a process that implements corrective actions to mitigate human health and environmental impacts. Site remediation typically includes investigation; interim actions such as providing bottled water; setting remediation goals; planning, designing and implementing remedial actions; monitoring progress and effectiveness; and closure. PFAS present unique challenges to effective remediation, including emerging regulation and low guidance values, resistance to degradation, and their unique environmental behavior (figure 18) [12].

*In situ* remediation involves treatment without first having to remove soil, groundwater or surface water from the environment. *In situ* remediation is particularly desirable for minimizing resources and financial costs required otherwise to excavate, pump, or transport affected materials, and the need for disposal at appropriately licensed on- or off-site treatment facilities. Organic contaminants, for example hydrocarbons and chlorinated hydrocarbons, have been successfully remediated *in situ* by a range of methods such as microbial degradation, chemical oxidation and soil smouldering, or removed by vapor extraction and thermal desorption. Metals (as cations and oxyanions) cannot be destroyed and are generally not volatile, but risk from metal contamination has been mitigated by immobilization with chemical amendments.

Perfluorinated carbon chains are extremely persistent in the environment due to strong carbonfluorine bonds and the tight sheath of electron-dense fluorine atoms shielding the alkyl chain core [241]. This means biodegradation is not a feasible option and chemical degradation is difficult. Volatilization can occur but requires intense energy inputs to remove less volatile longer chain PFAS, and risks degrading some PFAS to products that are difficult to characterize. For these reasons, *in situ* treatment of PFAS in both soil and groundwater has been largely limited to immobilization with chemical amendments. Addition of Portland cement has been used to stabilize soils for other contaminants but appears to have little direct benefit for PFAS [242].

Apart from the inherent properties of PFAS, regulatory uncertainty adds to the difficulties in confidently adopting *in situ* remediation approaches. While regulation of PFAS tends to vary by country, PFAS are often regulated at low nanograms per litre (ng L^−1^) in drinking water (e.g. US EPA MCLs of 4 ng L^−1^ for PFOA and PFOS [243]). Drinking water regulations often carry over to groundwater and soil. Such low regulatory values, and the mobility of many PFAS in water, mean that the volume of impacted soil and groundwater can be much larger than other contaminants and present serious challenges for engineering required to remediate to such levels [16].

For the reasons above, immobilization remains the only *in situ* approach widely applied for PFAS and further technological developments are needed, as discussed below.

### Current and future challenges

Technology for PFAS groundwater and soil remediation is rapidly developing, but there are serious limitations to available technologies for *in situ* destruction of PFAS.

Destructive technologies receive much attention in the literature, but for management of PFAS-contaminated sites, they play a relatively minor part, given that the masses of PFAS are small (due to the low concentration limits). Chemical oxidation or reduction are capable of *ex situ* destruction of PFAS [244]. However, given that contact between reagents and the contaminants is retarded in the subsurface, and interference of the aquifer matrix with chemical processes, and the inherent persistence of PFAAs, none appear viable for *in situ* treatment. Biological processes currently appear incapable of sufficiently defluorinating PFAS in timescales appropriate for remediation.

Current approaches therefore largely rely on breaking pathways to receptors. Capping and isolation to physically separate the contaminated soil from the surrounding environment, can work as well for PFAS as they do for other contaminants. Based on information provided by the Australian Department of Defence [245], capping has been implemented in combination with other technologies such as immobilization with AC, off-site treatment and disposal of the most contaminated soil. Adsorption onto amendments (AC, modified clays, ion exchange resins, etc) has been developed for *in situ* application in soil and groundwater [244, 246]. This technology has been used to treat soil at nine Australian Department of Defence facilities [245]. A soil column study indicated treatment using injectable AC increased retention of PFAS 8-fold [247] while a field study indicated initial concentrations of PFOS (280 ng L^−1^ to 1,450 ng L^−1^) and PFOA (490 ng L^−1^ to 3,260 ng L^−1^) were reduced to less than 40 ng L^−1^ in 6 wells [248] after a single application. Information provided by Regenesis, a purveyor of CAC for groundwater remediation, claims it has been used on more than 60 sites [249].

For groundwater treatment, permeable adsorptive barriers will eventually saturate if the source of PFAS is not mitigated, requiring ongoing reinjection. Immobilization has received some criticism for leaving PFAS in the environment, albeit with health and ecological risks managed, but the lack of viable destructive alternatives means *in situ* sorption is adopted widely. In the worst case, immobilization is no worse than pump and treat, which also leaves the vast majority of contaminant in the groundwater system in place. Immobilization can be less effective for short-chain PFAAs, depending on the adsorbent (e.g. [161]). Short-chain PFAAs are more mobile and may be increasing in the environment due to increasing use as replacements or from environmental degradation. Therefore, the ability to immobilize short-chain PFAS is an increasingly desirable objective.

Pumping and treatment is also available for PFAS remediation, with all its inherent limitations [250]. This can be implemented as part of hydraulic containment of a plume or treatment of water to manage a specific risk such as providing potable water. Foam fractionation has emerged for pump-and-treat and is particularly effective for removing long-chain PFAS which partition favorably to the air: water interface. In-well versions of this technology have been adapted for *in situ* flushing of soil or shallow groundwater to remove PFAS [251]. However, these systems impact a relatively small volume and have limited ability to treat short-chain PFAAs. For example, an in-well demonstration removed 19 grams of total PFAS with an estimated radius of influence of 1.5 meters, and reducing groundwater concentrations 50% [251].

Most remediation methods for PFAS, whether *in situ* or *ex situ*, have been principally applied to source areas with high concentrations of PFAS in soil or groundwater. For soil, due to the mobility of PFAS, and low health-based guidance values, the areas requiring remediation are frequently too great to responsibly remove and dispose or destroy the soil. This places pressure on the need for technologies that retain the soil matrix. Currently immobilization achieves this, but even that is limited to areas where amendment can feasibly be worked into soil without destroying ecological values, infrastructure, etc.

For groundwater, the only feasible technologies for PFAS remediation are pumping and treating the contaminated groundwater and immobilizations using AC or proprietary products. While both technologies are implementable, they leave most of the contamination in the ground and rely on controlling migration to mitigate risk. Other technologies have not demonstrated adequate effectiveness for consideration.

### Advances in science and technology to meet challenges

As discussed above, fundamental challenges require further advances for *in situ* PFAS remediation. There is still a need for technologies that destroy, remove or permanently immobilize PFAS *in situ*.

#### Ability to achieve very low treatment goals

Immobilization of PFAS in soils can achieve low leachability of long-chain PFAS to meet most treatment goals. *In situ* soil flushing/washing has potential for removing PFAS from soil source areas but is more useful as a ‘grab for mass’ to remove PFAS gross contamination from source areas, and not able to achieve very low treatment goals. There are no commercially mature methods for *in situ* destruction of PFAS in soil or groundwater. Research is underway to develop soil smouldering [252], which has been used *in situ* for less recalcitrant organic contaminants, but not to date for *in situ* PFAS treatment. There is need for considerable development of *in situ* technologies for separation or destruction of PFAS from soil and groundwater.

#### Ability to treat a wide range of PFAS with a large range of physicochemical properties

Advanced sorbents are being developed aiming to overcome the limitation of current sorbents for immobilizing or removing short-chain PFAAs. These include polymers, cyclodextrins and functionalized minerals and biochars. However, both cyclodextrins and functionalized media may lack environmental persistence, and therefore are more useful for *ex situ* separation techniques, rather than *in situ* approaches.

Much development of remediation technologies has been funded due to contamination from AFFF. As focus moves to PFAS in other products, a wider range of PFAS become remediation targets, especially with the development of advanced non-targeted analytical techniques which can detect a wide range of compounds. Therefore, research is needed to develop approaches to treat PFAS with more diverse structures and properties than the PFAAs and their precursors.

#### Ability to be applied and effective over large spatial scales

Current technologies are more effective or financially and sustainably viable for treating source areas with high PFAS concentrations, although for groundwater, treating the less contaminated toe of plumes can sometimes have financial and operational advantages. Immobilization using chemical amendments is one approach that is achieving success in treating larger areas, noting the limitations stated above. Other approaches currently being investigated include phytoremediation for soils and surface water. Phytoremediation has demonstrated some ability to remove PFAS from soil and water (e.g. [253]), although the length of time needed to reduce PFAS concentrations to acceptable levels by phytoremediation is not known. Plants preferentially take up short-chain PFAS. Some researchers are combining floating wetland plants with sorbents to manage both short-chain and long-chain PFAS, but the overall effectiveness is still unknown.

### Commercialization

Injectable *in situ* adsorbents, primarily ACs, were developed for other contaminants and then adapted to PFAS and are readily available and in widespread use. Pumping and treatment technologies are also widely available. Advanced adsorbents, especially those aiming to manage short chain PFAS, require further development and field demonstration before they can be commercialized. Soil smouldering has been demonstrated *ex situ*; but remains to be demonstrated *in situ*. Adding a source of combustible material *in situ* to achieve required temperatures for PFAS destruction remains a challenge. Other *in situ* technologies must be demonstrated in the natural environment, which can be challenging, to provide confidence to environmental regulators. This is more difficult than demonstrating *ex situ* technologies, which are demonstrated under more controlled conditions.

### Concluding remarks

The discussion above has highlighted some important challenge to be overcome for effective *in situ* remediation of PFAS impacted soil and groundwater. Other challenges remain such as a changing landscape of regulatory approaches. Health-based guidance values and values for ecological protection have changed regularly over the last two decades and often differ between jurisdictions. Recently there has been a growing awareness of volatile PFAS. This may have relevance for requirements for pollution controls in wastewater treatment plants and landfills. Formation of volatile PFAS may also have relevance if destructive *in situ* technologies are developed.

These changes place additional pressures on remediation technologies and may favor those better able to treat a range of PFAS with diverse properties. Further development is greatly needed to shift the balance for *in situ* remediation from immobilizations to removal, while still considering overall sustainability of remedial options. *In situ* destruction remains a great challenge, and even where effective may be limited in scope to source areas and immediate surrounds. Thus there may be an enduring need for institutional controls (e.g. dietary advice, fisheries advice) and point of use treatment of water.

## Acknowledgements

This contribution represents the views of the authors based on our experiences, engagement with the literature and many conversations with colleagues, clients and contacts around the world. No funding was received for authoring this contribution.

## 16. Roadmap summary and concluding remarks

### Lokesh P Padhye^1,2,3,4^, Melanie Kah^5^ and Erin M Leitao^6,7^

^1^ The New York State Center for Clean Water Technology, Stony Brook University, Stony Brook, NY 11794, United States of America

^2^ School of Marine and Atmospheric Sciences, Stony Brook University, Stony Brook, NY 11794, United States of America

^3^ Department of Civil Engineering, Stony Brook University, Stony Brook, NY 11794, United States of America

^4^ Department of Civil and Environmental Engineering, The University of Auckland, Auckland 1010, New Zealand

^5^ School of Environment, The University of Auckland, 23 Symonds Street, 1010 Auckland, New Zealand

^6^ School of Chemical Sciences, The University of Auckland, 23 Symonds Street, 1010 Auckland, New Zealand

^7^ The MacDiarmid Institute for Advanced Materials and Nanotechnology, Victoria University of Wellington, Wellington, New Zealand

This PFAS Roadmap synthesizes knowledge from regulatory frameworks to destructive technologies, drawing on the detailed analyses in Sections 2–15. Collectively, this roadmap shows that PFAS management is inherently multi faceted, requiring coordinated advances in regulation, monitoring, fate understanding, separation, and destruction. No single intervention is sufficient; instead, integrated approaches tailored to matrix, PFAS profile, and scale are essential.

Regulations (section 2) highlight the fundamental challenge: the diversity of PFAS properties and extremely low regulatory/guidance values create tension between what can be measured and what can be remediated to the required level. While historical contamination drives much current regulation, achieving meaningful risk reduction will require advances in analytical chemistry, fate and toxicity assessment, and treatment.

Alternatives (section 3) show that phase-outs are underway for non-essential uses, with categorization based on chemical function, end-use, or service. Databases exist to capture knowledge on alternatives, but information sharing is limited. Only a small fraction of applications currently has suitable alternatives, and challenges remain in managing blanket bans, ensuring viable substitutes, and aligning phase-out timelines with market readiness. Gaps include developing safe, sustainable substitutes for essential uses, assessing all substitutions for safety and environmental fate, and building a better understanding of why PFAS are used in particular sectors through improved stakeholder communication.

Analytical (section 4) capabilities are central to progress. Complementary techniques are being developed, yet standardized and automated extraction methods, critical for improving sensitivity, reproducibility, accuracy, precision, and fluorine mass balance, remain a gap.

Monitoring (section 5) is still an emerging field but is vital for quantifying PFAS exposure sources across diverse populations. Key learnings include the value of air monitoring and prioritizing PFAS classes such as sulfonamide precursors based on serum measurements. PFAS mixture signatures can help identify exposure sources and test toxicity. The field would benefit from broader inclusion of underrepresented PFAS classes and improved linkage between environmental monitoring and human biomonitoring.

Partitioning and Fate (section 6) challenges classical paradigms and models, which fail to capture PFAS behavior. Current datasets are limited and not representative of PFAS diversity. Key gaps include building broader, more representative data to support model development, and advancing the emerging field of ion partitioning as well as interfacial adsorption, especially at air–water interfaces, for chemicals with varying degree and patterns of fluorination.

Adsorption and Applications (section 7) remain central to soil and groundwater remediation, but a universal sorbent is unlikely given PFAS diversity. Performance is strongly influenced by the water matrix. More sustainable approaches are needed for managing spent sorbents. Evaluation of new materials under realistic field conditions, particularly in the presence of complex DOM, remains an important research priority.

Enrichment Techniques (section 8), including foam fractionation, membranes, and ion exchange, offer effective pre-concentration. Foam fractionation and membranes are already applied at full scale but struggle with short-chain PFAS, while ion exchange resins perform better for short chains only in the absence of long chains, an issue solvable by placing AC upstream. Key needs include technology to prevent atmospheric PFAS losses, improving the regenerability of ion exchange resins, and developing these approaches alongside destruction techniques.

Hazardous Waste Incineration (section 9) can mineralize PFAS to HF, CO_2_, and H_2_O and is currently the best option for treating large PFAS-contaminated waste volumes, reducing future legacy pollution from stockpiling. Challenges include poor mixing, incomplete combustion, CF_4_ formation, and the need to better understand and avoid volatile reaction products. Optimization, improved sampling and analysis, predictive tools, and mechanistic studies, particularly at bench scale, would strengthen the technology.

Pyrolysis (section 10) provides a lower-energy alternative to incineration, partially destroying PFAS while producing biochars that can serve as sorbents. Gaps include closing the fluorine mass balance in full-scale systems, generating complete emissions data, identifying optimal operational parameters, tailoring biochar properties for PFAS sorption, verifying long-term stability, and assessing scale-up via lifecycle and techno-economic studies.

Supercritical Water Oxidation (section 11) mineralizes PFAS rapidly with no NO*_x_*/SO*_x_* by-products and is adaptable to various wastes. Remaining gaps include closing the fluorine budget by measuring volatile/semi-volatile organofluorines, understanding the influence of water chemistry and co-contaminants on reaction performance, addressing precipitation, corrosion, and hot spots, and developing predictive models for different reactor designs.

Hydrothermal Alkaline Treatment (section 12) is effective for the full PFAS spectrum, including ultra-short chains, and can process high-TDS liquids. Research needs include quantifying reaction windows and stoichiometry for each subclass, resolving pathways and mass balances in complex matrices, understanding co-contaminant effects, evaluating HALT as a polishing step or for sorbent regeneration, and conducting life cycle and safety analyses to guide field deployment.

Ball Milling (section 13) is effective for solid matrices, having shown feasibility for PFAS-laden solids contaminated with AFFF. Gaps include assessing scalability, testing across more PFAS types, and advancing mechanistic understanding with analytical tools.

Electrochemical Oxidation (section 14) uses catalytic anodes for reagent-free PFAS destruction at ambient conditions, with a lower energy demand than many destructive options. Advances in low-cost, high-surface-area electrodes could expand use from POU to full scale systems. Gaps include designing affordable, durable electrodes resistant to HF corrosion, developing selective ‘concentrate-and-destroy’ surfaces, minimizing unwanted by-products, defining optimal operating parameters, and generating pilot-scale cost and performance benchmarks.

*In Situ* remediation (section 15) addresses PFAS in soils and groundwater, with immobilization as the most widely applied method. Challenges include co-contaminants, low PFAS concentrations, and the difficulty of removing PFAS without disturbing the matrix. Research needs to focus on treating diverse PFAS, especially mobile short chains, over large spatial scales, and achieving treatment goals in shorter timeframes.

From these sections, several overarching conclusions emerge. First, the diversity of PFAS, both structurally and in their environmental behavior, is a major challenge in all three pillars: monitoring, regulating and remediating. For instance, remediation of PFAS demands treatment trains and tailored solutions rather than one-size-fits-all approaches. Second, nearly all technologies face greater difficulty with short-chain and ultra-short-chain PFAS, which remain a persistent gap across separation and destruction methods. Third, achieving and verifying complete destruction requires rigorous fluorine mass balance, integrating targeted, non-targeted, and total fluorine analyses across all phases. Fourth, integration is key: enrichment techniques paired with effective destruction methods can overcome many energy, cost, and efficiency barriers. Finally, deployment at scale will require optimization not just of chemistry but of engineering, operations, and monitoring, alongside regulatory acceptance grounded in proven performance data.

**Figure 19.**
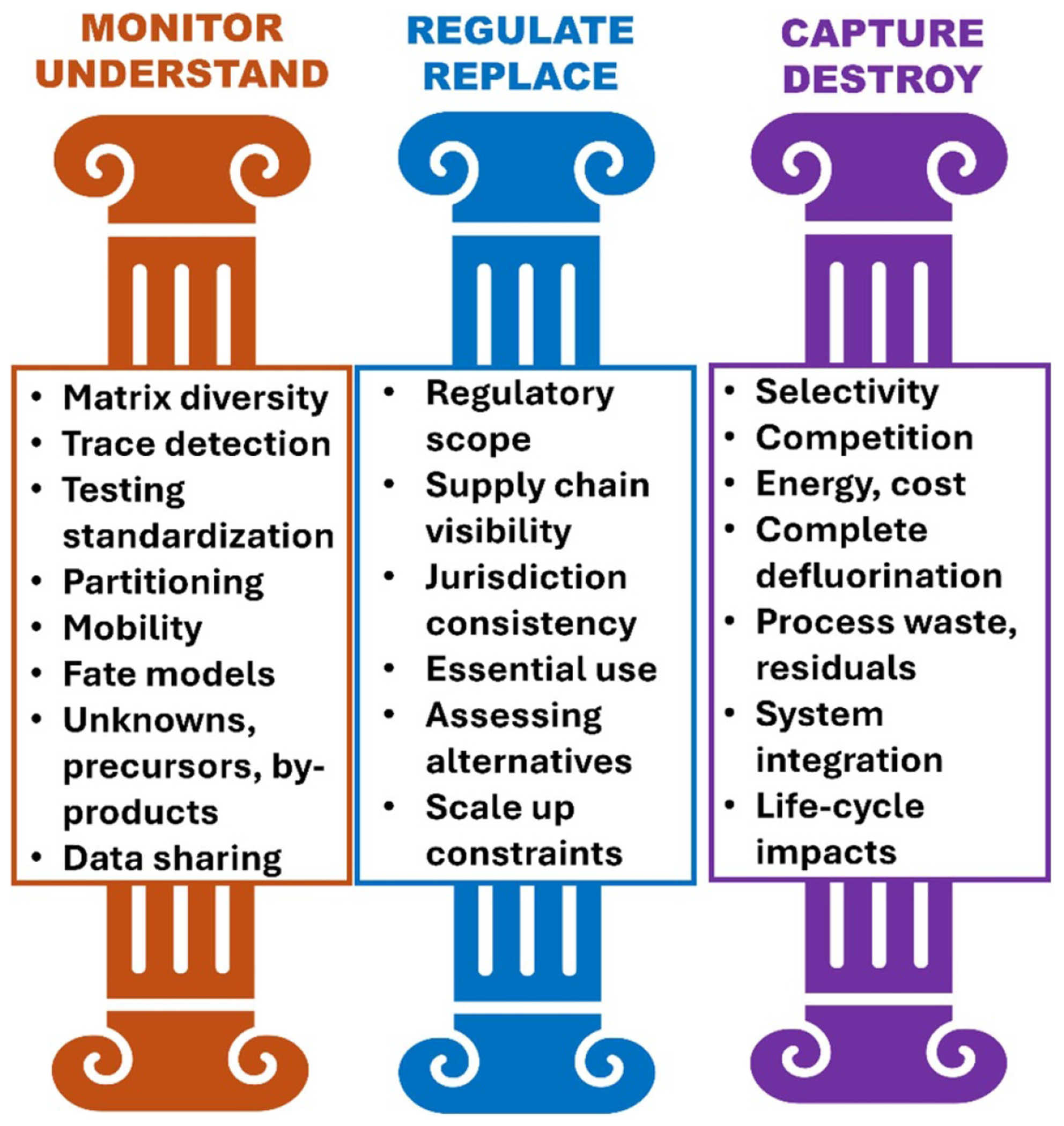
Future challenges to address in the management of PFAS in each of the three essential pillars.

By addressing the specific gaps identified in each section (figure 19) and building on the key learnings documented in this roadmap, the PFAS community can accelerate the transition from long-term containment to PFAS destruction, providing solutions that meet both environmental and societal expectations. Achieving this vision will require more than incremental technical advances; it will demand the coordinated efforts of scientists, engineers, regulators, industry leaders, and community stakeholders. Only through the sustained collaboration of transdisciplinary teams, integrating expertise from chemistry, toxicology, materials science, environmental engineering, policy, and public health, can we ‘together’ develop, validate, and deploy the integrated strategies needed to overcome one of the most complex and pressing environmental challenges of the 21^st^ century.

## Figures and Tables

**Table 1.** Overview numbers on uses of PFAS included in the database, and the functions they provide (bolded numbers are the grand totals for each column).

Use categories	Sub-uses	Applications	Chemical functions	End-use functions	Services
Active pharmaceutical ingredients (API)	2	14	1	24	21
Biocides (BP)	1	4	1	4	4
Building and construction products (Build.)	9	17	14	18	30
Consumer mixtures (Consu.)	7	15	7	15	12
Cosmetic products (Cosm.)	5	32	9	12	6
Electronics and semiconductors sector (Elec.)	3	29	17	22	37
Energy sector (Energy)	9	19	17	19	24
Firefighting foams (FFF)	1	5	1	1	3
Fluorinated gases (F-gases)	7	29	8	14	27
Food contact materials (FCM)	2	4	4	4	9
Industrial production (Indust.)	8	28	12	18	11
Lubricants (Lubr.)	3	42	11	13	19
Medical products (Med.)	6	21	14	18	29
Metal plating and metal products manufacture (Metal)	2	4	8	10	14
Petroleum and mining (Mining)	2	9	10	13	15
Plant protection products (PPP)	1	6	3	7	5
Textile, upholstery, leather, apparel, and carpets (TULAC)	7	20	11	15	21
Transport sector (Transp.)	10	27	16	24	45
Grand Total	**85**	**325**	**39**	**131**	**201**

**Table 2.** Analytical toolbox for PFAS environmental monitoring.

Analytical method	Strengths	Weaknesses
Targeted analysis *liquid and gas chromatography tandem mass spectrometry*	• Quantitative • Low detection limits • Complete structural information • Standardized methods • Formal reproducibility criteria	• Highly limited by available chemical standards • Underestimates total magnitude of PFAS
Total oxidizable precursor (TOP) assay	• Quantitative • Low detection limits • Formal reproducibility criteria • Not limited by availability of chemical standards	• Underestimates total magnitude of PFAS • Does not provide complete chemical structure • Non-standardized method
Bulk organofluorine measurements *combustion ion chromatography; ^19^F nuclear magnetic resonance; inductively coupled plasma mass spectrometry*	• Quantitative • Estimates total magnitude of contamination • Formal reproducibility criteria • Not limited by availability of chemical standards	• High detection limits • Sample preparation does not retain all PFAS • Potential interferences from non-PFAS organofluorine • Does not provide complete chemical structures • Non-standardized methods
High resolution mass spectroscopy (HRMS)	• Highly sensitive • Complete structural information • Not limited by availability of chemical standards • Non-targeted compound discovery	• Qualitative to semi-quantitative • Suspect screening libraries not inclusive of all PFAS • Non-standardized methods • Limited reproducibility criteria

**Table 3.** Summary of HALT field demonstrations performed at the time of writing.

	Alaska DOT&PF	3M Company	Brice Environmental	US Army Corps of Engineers	Washington Department of Ecology	Env. Security Technology Certification Program (ESTCP)
Site	Fairbanks International Airport	Confidential	Beale Air Force Base	Beale Air Force Base	City of Tacoma Wastewater Treatment Plant	Clean Earth Facility, Charlotte, NC
Timing	August, 2023	April, 2024	May, 2024	May, 2024	Feb, 2025	May, 2025
Water Matrix	Foam Fractionate (Fire Training Pit)	High TDS Wastewater	Foam Fractionate (Groundwater)	AFFF	Foam Fractionate (Municipal Wastewater)	Foam Fractionate (Groundwater), Sorbent Regeneration Brines (Groundwater), AFFF
Volume Treated	~1350 gallons	~1100 gallons	~330 gallons	~110 gallons	~150 gallons	~650 gallons

**Table 4.** Example list of analytical techniques to characterize PFAS destruction by ball milling.

	Technique	Output
PFAS Analysis	Liquid chromatography tandem mass spectrometry Combustion ion chromatography High resolution mass spectrometry	Quantitation of target PFAS present in samples. Total PFAS (indiscernible) present in samples. Identification and semi-quantitation of non-target PFAS.
Structural Dynamics	Solid-State Nuclear Magnetic Resonance Electron Paramagnetic Resonance Powder x-ray Diffraction Scanning Electron Microscopy X-ray Photoelectron Spectroscopy BET Surface Area Analysis	Fluorine bonding environments and structural transformation. Identification of mechanoradicals and structural transformation. Structural characterization. Surface topography, morphology, and size. Chemical composition properties and binding energies. Determination of available ‘reactive’ surfaces.

## Data Availability

All data that support the findings of this study are included within the article (and any supplementary information files).
